# Genomic and pathogenicity analyses to identify the causative agent from multiple serogroups of non-O1, non-O139 Vibrio cholerae in foodborne outbreaks

**DOI:** 10.1099/mgen.0.001364

**Published:** 2025-02-26

**Authors:** Masatomo Morita, Hirotaka Hiyoshi, Eiji Arakawa, Hidemasa Izumiya, Makoto Ohnishi, Kikuyo Ogata, Mari Sasaki, Hiroshi Narimatsu, Emiko Kitagawa, Yukihiro Akeda, Toshio Kodama

**Affiliations:** 1 Department of Bacteriology I, National Institute of Infectious Diseases, Tokyo, Japan; 2 Department of Bacteriology, Institute of Tropical Medicine, Nagasaki University, Nagasaki, Japan; 3 Food and Environment Division, Oita Pharmaceutical Association Inspection Center, Oita, Japan; 4 Section in Charge of Microbiology, Oita Prefectural Institute of Health and Environment, Oita, Japan; 5 Ishikawa Prefectural Institute of Public Health and Environmental Science, Kanazawa, Japan

**Keywords:** foodborne outbreak, non-agglutinable vibrios, type III secretion system, *Vibrio cholerae*

## Abstract

In 2013, foodborne outbreaks in Japan were linked to non-O1, non-O139 *Vibrio cholerae*. However, laboratory tests have detected several serogroups, making it difficult to determine the causative agent. Therefore, whole-genome analyses revealed that only serogroup O144 *V. cholerae* possesses a genomic island with a type III secretion system (T3SS). A T3SS-deficient mutant was subsequently generated, and its pathogenicity was assessed using a rabbit ileal loop test. This led to the conclusion that serogroup O144 *V. cholerae* with T3SS was the causative agent of foodborne outbreaks. This study provides an illustrative example of the utilization of whole-genome data for pathogenicity and molecular epidemiological analyses in outbreak investigations.

­

**Outcome**

Multiple serogroups of non-O1, non-O139 *Vibrio cholerae* strains have been isolated from foodborne outbreaks in Japan. The core genome phylogeny of isolates from patients and food enabled the estimation of the causative serogroup, in which pan-genome analyses identified virulence factors with experimental evidence.

Determining the causative agent is important for appropriate public health treatment. However, contamination with mixed bacterial populations presents significant challenges. Here, we demonstrate that a genomic approach can support conventional microbiological methods, facilitating the identification of pathogens with exhaustive gene profiles.

## Data Summary

Short-read sequence data were submitted to the DDBJ Sequenced Read Archive, with accession numbers listed in Table S1. The high-quality finished sequences of *Vibrio cholerae* O144 strain V130003 and their annotations are available in GenBank/EMBL/DDBJ under the accession numbers AP024967 for chromosome 1 and AP024968 for chromosome 2.

## Introduction

*Vibrio cholerae* has ~200 serogroups based on the O-antigen, which is the outermost portion of the bacterial lipopolysaccharide, and only two serogroups, O1 and O139, are usually associated with cholera epidemics [1,2]. However, isolates of other serogroups, collectively referred to as non-O1, non-O139 *V. cholerae* (or non-agglutinable vibrios), have raised public health concerns due to their associations with sporadic diarrhoea [3,6]. Notably, virulence determinants are diverse within non-O1, non-O139 *V. cholerae* because of their heterogeneous population structure, including the MARTX toxin, heat-stable enterotoxin, haemolysin, hemagglutinin/protease, type III secretion system (T3SS), type VI secretion system and others that are detected and considered to be virulence factors [7,12]. Nevertheless, while reports on the gene profiles of these factors exist, the extent to which these factors contribute to diarrhoea has rarely been investigated [13]. Consequently, the mechanism of diarrhoea induction in the majority of non-O1, non-O139 *V. cholerae* remains unclear, and their diversity in aquatic environments makes it difficult to determine the pathogenic strains.

In 2013, three foodborne outbreaks caused by non-O1, non-O139 *V. cholerae* were reported from two prefectures in Japan, and epidemiological investigations suggested the consumption of veined rapa whelk as the source of these outbreaks. However, isolates from patients and food were classified into multiple serogroups, and the causative serogroups of non-O1, non-O139 *V. cholerae* were not determined in the preliminary investigation. In this study, we performed the analyses based on whole-genome sequencing of isolates to identify the serogroups and virulence factors of non-O1, non-O139 *V. cholerae* causing foodborne outbreaks and to elucidate virulence determinants. As a result, pathogenic strains can be identified from diverse populations, which could be applied to methods for assessing the risk of infection in the environment.

## Methods

### Isolates from foodborne outbreaks

A total of 64 non-O1, non-O139 *V. cholerae* isolates from 3 foodborne outbreaks were sent to the Department of Bacteriology I, National Institute of Infectious Diseases (NIID), for serogroup and molecular epidemiological analyses; 33 isolates were from 10 faecal samples of patients, and 31 isolates were from 6 food samples (Table S1, available in the online Supplementary Material). The serogroups were determined using a slide agglutination test with rabbit antisera prepared at the NIID. For strains isolated from ten faecal and six food samples, one strain from each sample was subjected to whole-genome sequencing. When multiple serotypes were detected from one sample, one strain was selected for all serotypes. In total, 25 strains were used for whole-genome sequencing.

### Whole-genome sequencing and genome assembly

Genomic DNA was extracted using a DNeasy Blood and Tissue Kit (QIAGEN) for short-read sequencing, and the concentrations were determined using a Qubit dsDNA HS assay kit (Thermo Fisher Scientific). The genomic library was prepared using the Nextera XT DNA Library Preparation Kit (Illumina), and paired-end short reads were sequenced using a HiSeq 2500 or MiSeq (Illumina). Genome assembly was performed using SPAdes v.3.13.1 with the ‘--careful’ option and a read coverage cutoff value of 10 [14].

Additionally, the genomic DNA of *V. cholerae* O144 strain V130003 was extracted using a Genomic-tip 100 G^−1^ (QIAGEN) and used for long-read sequencing on PacBio RS II (Pacific Biosciences). A genomic library for P6-C4 chemistry was prepared using an RS II SMRTbell template preparation kit (version 1.0; Pacific Biosciences). Sequencing reads were assembled using the Hierarchical Genome Assembly Process 3 [15]. To complete the genome sequence of strain V130003, PCR primer sets were designed to cover the gaps between the contigs, and the amplicon sequences were confirmed by Sanger sequencing. The complete genome and genome assemblies were annotated using the DDBJ Fast Annotation and Submission Tool (https://dfast.ddbj.nig.ac.jp/) [16].

### Pan-genome and phylogenetic analysis

Pan-genome profile of 25 strains sequenced in this study was constructed using Roary v.3.13.0 with the ‘-s’ option [17]. To estimate virulence factors, only the unique coding sequences (CDSs) across all strains of serogroup O144 were selected from the pan-genome profile and manually characterized using an National Center for Biotechnology Information (NCBI) blastp search. The genomic locations were determined according to the annotation of the V130003 complete genome.

Phylogenetic analysis was carried out on 27 strains, of which 25 strains were sequenced in this study and 2 serogroup O144 strains from the public database (BioSample: SAMD00180521 and SAMN02693885). Single nucleotide variants (SNVs) were extracted by BactSNP v.1.1.037, with the genome of *V. cholerae* O144 strain V130003 as a reference, and were subsequently used for phylogenetic relationships by reconstructing a phylogenetic tree using IQ-TREE v.2.1.2 with 1000 ultrafast bootstrap replicates [18,20]. The phylogenetic tree was visualized using iTOL [21]. We calculated the pairwise SNV differences among ten O144 isolates sequenced in this study using core genome SNVs extracted by BactSNP v.1.1.037, with the genome of *V. cholerae* O144 strain V130003 as a reference [18]. The position of SNVs on the V130003 genome and amino acid substitutions is listed in Table S4.

### Construction of deletion mutants

Mutant strains of non-O1, non-O139 *V. cholerae* isolates were generated using the double-crossover homologous recombination method [22]. We selected the food-derived strain V130055 for the parent of deletion mutants, which was considered to be the causative agent. Upstream and downstream regions of the *hlyA* gene were amplified by PCR using two primer sets: NAG-dhlyA-1 [5′-CAC ACG CAA ACT CAA GGA TGA CG-3′] and NAG-dhlyA-FRT-2 [5′-GAA GCA GCT CCA GCC TAC ACA ATC GAT GAA TGC CGG CTC CCG TGC-3′] and NAG-dhlyA-FRT-3 [5′-GGA ATA GGA ACT AAG GAG GAT GTG GCG CTG ACA TTC ACC TAC C-3′] and NAG-dhly-4 [5′-GGT CGG TGC GTA TTT ATC GCG-3′]. Furthermore, a chloramphenicol-resistant cassette containing the flippase recognition target (FRT) at both sequence ends was cloned from pKD3 by PCR using the primers pKD-FRT-Fw [5′-GAT TGT GTA GGC TGG AGC TGC TTC-3′] and pKD-FRT-Rv [5′-ATC CTC CTT AGT TCC TAT TCC-3′]. The three fragments were subsequently combined by a second PCR using primers NAG-dhlyA-1 and NAG-dhlyA-4. The combined fragments were transformed using a previously described natural transformation method [23]. Briefly, *V. cholerae* isolates were cultured overnight, resuspended in 1 ml of defined artificial seawater medium [40 g l^−1^ sea salt (Sigma-Aldrich), 25 mM HEPES (Nacalai Tesque), pH 7.4], and pieces of sterilized shrimp shells were added to the bacterial suspension. After 24 h of static incubation at 37 °C, the bacteria adhered to the shrimp shells were dislodged by vigorous vortexing and spread onto Luria-Bertani (LB) agar plates containing the appropriate antibiotic. The following day, colonies on the plates were confirmed as *hlyA* deletion mutants by colony PCR and their haemolytic activity on TSA 5% sheep blood agar (Becton, Dickinson and Company). Similarly, the *vcsV2* deletion mutant was generated using primer sets NAG-dvcsV2-1 [5′-CTT GAA TCA AGC TTA CCC TTG ATG G-3′] and NAG-dvcsV2-FRT-2 [5′-GAA GCA GCT CCA GCC TAC ACA ATC CGC AAT CAT GAC ACT ACT GGG-3′] and NAG-dvcsV2-FRT-3 [5′-GGA ATA GGA ACT AAG GAG GAT TAC GGA GAC TAT CCA TAT TTA ACG-3′] and NAG-dvcsV2-4 [5′-CTC ACC CTG CTT CAT CTT GC-3′]. A kanamycin-resistance cassette containing FRT at both sequence ends was also cloned from pKD4 by PCR using the primers pKD-FRT-Fw and pKD-FRT-Rv. The deletion of *vcsV2* was confirmed by a defect in T3SS2 translocon protein secretion in a Western blotting assay.

### Western blotting assay

To confirm the production and secretion of VspB2 (*Vibrio parahaemolyticus* VopD2 homolog) on T3SS, bacterial pellets and culture supernatants were collected and subjected to SDS-PAGE as previously described [24]. Proteins were transferred onto membranes (Millipore), probed with an anti-VopD2 polyclonal antibody and subsequently with a horseradish peroxidase-conjugated goat anti-rabbit IgG antibody (Invitrogen). Blots were developed using the ECL Prime Western Blotting Detection Reagent (Amersham Biosciences).

### Rabbit ileal loop test

The rabbit ileal loop test was conducted as previously described [25]. Isogenic mutant strains of O144 *V. cholerae* strain V130055 (10^9^ c.f.u./loop in PBS) were injected into ligated rabbit ileal loops, and fluid accumulation in each loop was measured 18 h post-injection. The fluid accumulation ratios were calculated as the amount of accumulated fluid (ml) per length (cm) of the ligated rabbit small intestine. The graph represents the mean values from at least five independent experiments. For statistical analysis of the animal experiments, one-way ANOVA was used and *P*<0.05 was considered significant.

## Results

### Epidemiological investigation

From September to October 2013, three outbreaks caused by non-O1, non-O139 *V. cholerae* were reported from two prefectures in food poisoning statistics managed by the Ministry of Health, Labour and Welfare. Because non-O1, non-O139 *V. cholerae* are rare causative agents of food poisoning in Japan, an unrecognized epidemiological link potentially exists between these outbreaks. Prefectural public health institutes isolated non-O1, non-O139 *V. cholerae* strains from foods using veined rapa whelk processed by a manufacturer as a common ingredient. It is believed that non-O1, non-O139 *V. cholerae* could not be sterilized because of inadequate treatment by the manufacturer. Consequently, 446 of 918 individuals who consumed contaminated food exhibited symptoms of diarrhoea and abdominal pain.

### Serogroups and phylogenetic analysis

**Fig. 1.**
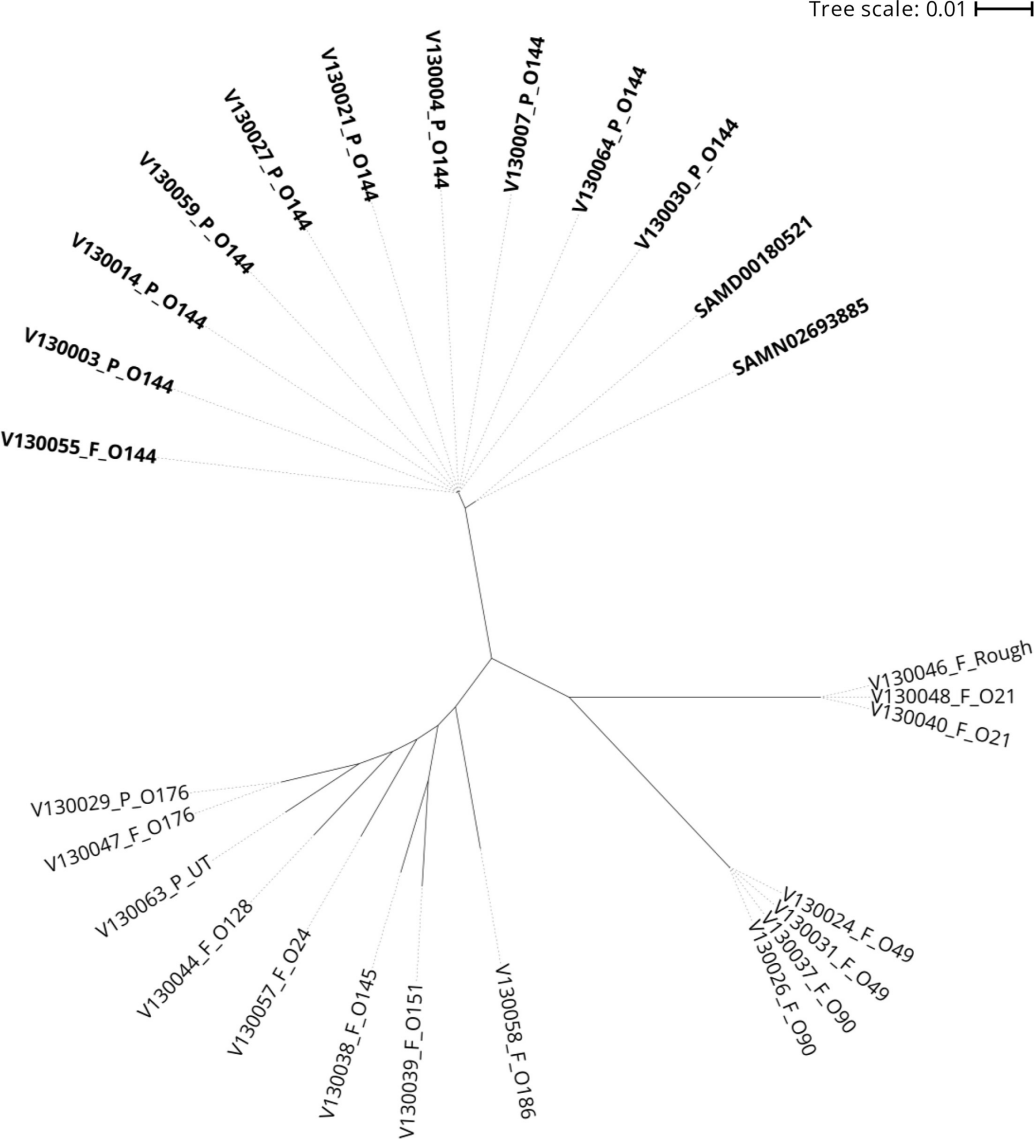
Phylogenetic tree based on core genome SNVs of non-O1, non-O139 *V. cholerae* isolates from the foodborne outbreaks. Source [patient (P) or food (F)] and serogroup are indicated after the strain name. The serogroup O144 strains are indicated in bold. Two O144 strains from the public database were included in the analysis.

Serogroups of the non-O1, non-O139 *V. cholerae* isolates (*n*=64; 33 isolates from patients and 31 isolates from food) were determined using slide agglutination tests. Eleven serogroups were identified among 61 isolates, and the other 3 isolates were classified as R, which lost the production of the O-antigen or were untypeable (UT). The serotyping results were as follows: O21 (*n*=7), O24 (*n*=1), O49 (*n*=9), O90 (*n*=4), O128 (*n*=1), O144 (*n*=32), O145 (*n*=2), O151 (*n*=1), O176 (*n*=2), O186 (*n*=1), O192 (*n*=1), R (*n*=1) and UT (*n*=2) (Table S1). The majority of the 33 isolates from the patients were O144 (30/33), whereas the others included O176 (1/33) and UT (2/33). However, the serogroups of isolates from foods were diverse, and the serogroups common to patients and foods were O144 and O176. These results suggested that foods were contaminated with multiple populations of non-O1, non-O139 *V. cholerae*, of which serogroup O144 was most likely to be pathogenic. However, O176 and UT may also be pathogenic if derived from O144 via serogroup conversion. To confirm this possibility, we performed a phylogenetic analysis using SNVs in the core genome of 27 strains, which included 25 strains from the present study and two O144 strains from the public database. As a result, strains of O49 and O90 formed clusters, and the other isolates were clustered based on serogroups, regardless of their source. Both the O176 and UT isolates were distinct from the O144 isolates, indicating that these isolates were not derived from O144 strains with serogroup conversion. The two O144 strains from the public database were isolated in India and clustered, with a different cluster from the other O144 strains (Fig. 1).

**Fig. 2.**
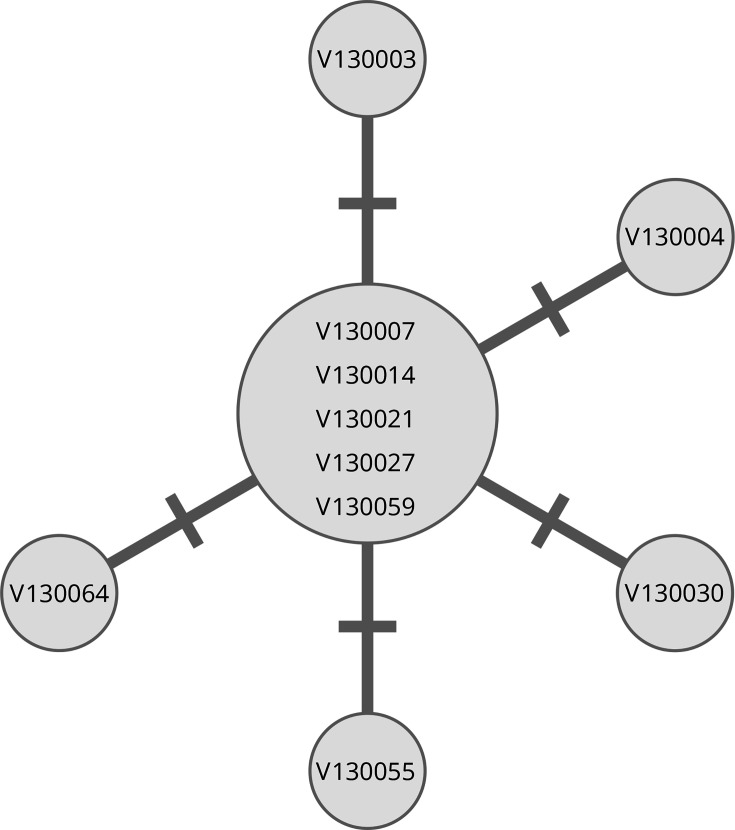
Median-joining tree of the O144 *V. cholerae* isolates. The number of short lines on connecting lines between circles represents the number of SNVs.

The pairwise SNV differences among the ten O144 strains, which were nine isolates from patients and one isolate from food, were calculated using strain V130003 as a reference. A total of five SNVs were detected on chromosome 1. Of these, four SNVs were located on the CDS and one SNV was located in a non-coding region; however, these mutations were not considered to be associated with pathogenicity (Table S4). The median pairwise SNV distance was 1 (range 0–2), and the five strains showed identical SNV patterns (Fig. 2), suggesting that foodborne outbreaks were considered to be caused by a single *V. cholerae* serogroup O144 clone contaminated in food.

### Genes associated with virulence in *V. cholerae* O144 strains

Of the multiple serogroups detected, the O144 strains were thought to be the causative agents. However, the virulence factors have not yet been identified. To identify virulence factors, specific CDSs possessed by all O144 strains isolated in this study were extracted from the pan-genomic profile (Table S2). A total of 228 CDSs were found only in the O144 strains, of which only the genes for the T3SS were identified as candidate virulence factors from the NCBI blastp search. The T3SS gene cluster is widely distributed among *Vibrio* spp., and it is phylogenetically classified into T3SS1 and T3SS2. Further, the T3SS2 is categorized into two phylotypes, namely T3SS2α and T3SS2β [26,27]. The gene organization and annotation of the V130003 strain revealed that O144 strains harbour the *V. cholerae* T3SS2 core region, which was categorized as the T3SS2α clade based on sequence similarity. This T3SS2α was found to be inserted in the same genomic location as that observed in another T3SS2α-positive *V. cholerae* strain AM-19226 (Fig. S1) [28]. As *V. cholerae* strains that are positive for T3SS2 encode thermostable direct haemolysin (TDH) and/or TDH-related haemolysin (TRH) on the T3SS2 genomic island, we also identified *trh* in the region surrounding T3SS2α in the V130003 genome [29]. However, because TRH in *V. cholerae* has been reported as an accessory virulence factor, T3SS2 is considered the major virulence factor in the O144 strains [30].

### Enterotoxicity of T3SS2 on *V. cholerae* O144 strains

**Fig. 3.**
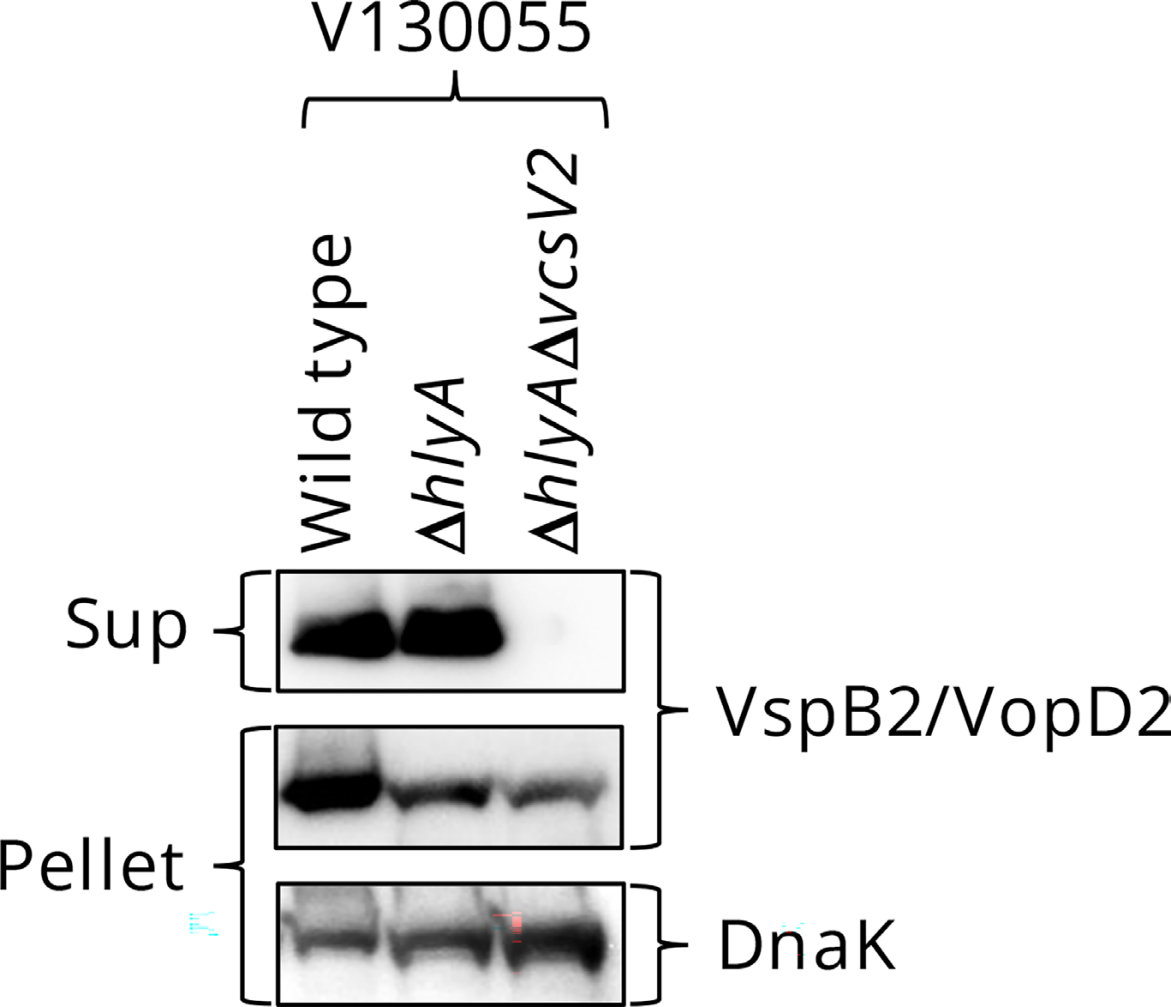
Immunoblot analysis of VspB2/VopD2 in culture supernatants (Sup) and bacterial pellets (Pellet) of the O144 *V. cholerae* strain V130055 and its derivatives. DnaK was used as a loading control.

**Fig. 4.**
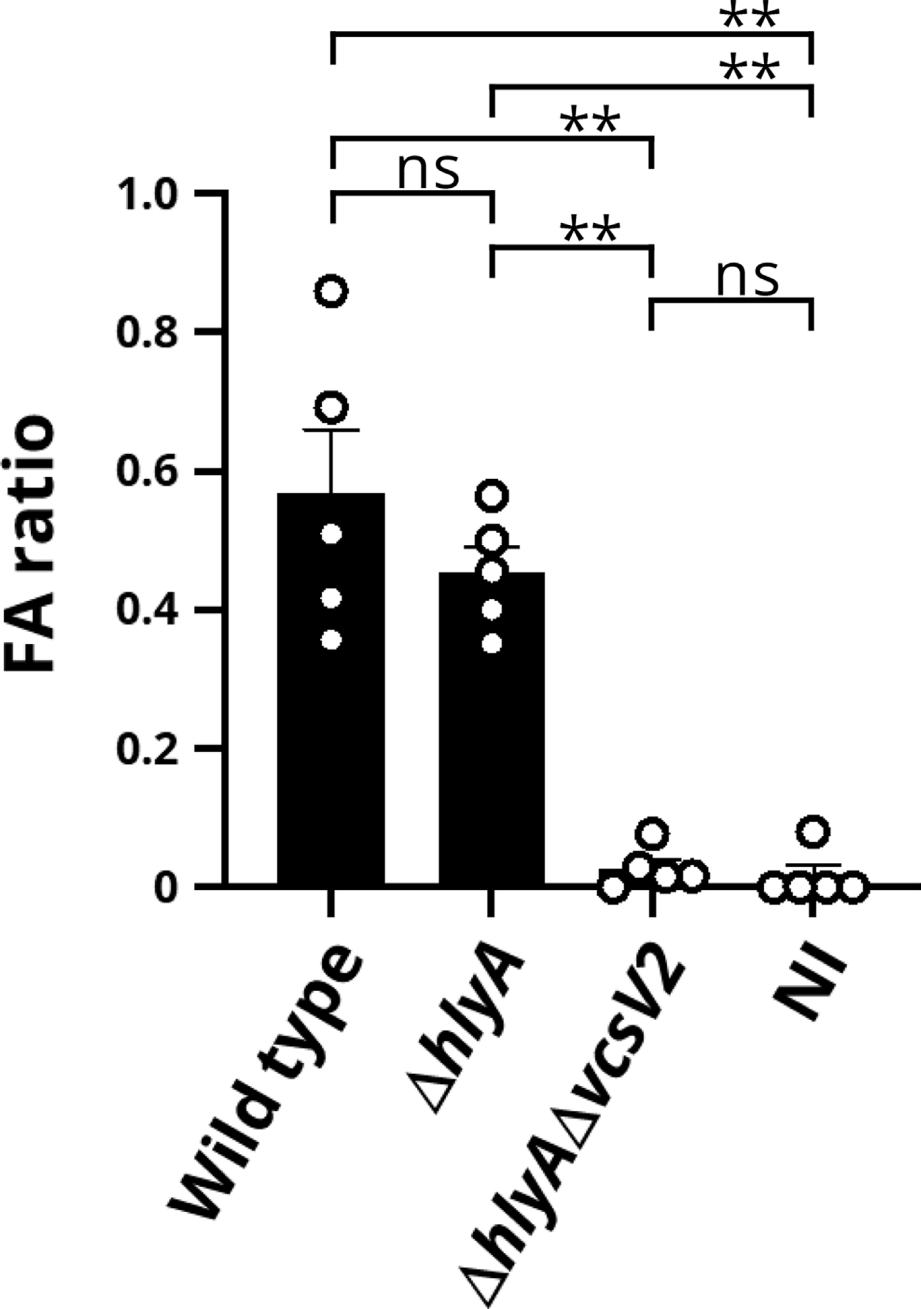
Enterotoxic activities of serogroup O144 *V. cholerae* strain V130055 and its derivatives were assessed using the rabbit ileal loop test. LB broth was used as a non-infected control (NI). The fluid accumulation (FA) ratio is the amount of accumulated fluid (ml) per length (cm) of the ligated rabbit small intestine. Statistical significance was determined by one-way ANOVA. **P<0.01; ns, *P>*0.05.

We investigated whether T3SS2 found in the O144 strains is functional and contributed to enterotoxicity. A previous study using a rabbit ileal loop test reported that haemolysin (a cytolysin encoded by the *hlyA* gene) of *V. cholerae* biotype El Tor also contributes to enterotoxicity independent of T3SS2 [25]. To exclude the effect of cytolysin on enterotoxicity, we constructed a double gene-deficient strain lacking both *hlyA* and *vscV2* (the structural gene of T3SS2) (*∆hlyA∆vscV2*) from the food-derived strain V130055 and examined the effect of this deletion on the secretion of VspB2, a translocator of T3SS2, by immunoblotting (Fig. 3). The VspB2 protein in the supernatant was not affected by the deletion of *hlyA* (*∆hlyA*) but was abolished in the *∆hlyA∆vscV2* strain, indicating that T3SS2 in the strain V130055 has secretory activity for VspB2. The secretion of VspB2 from strain V130003 isolated from the patient was also confirmed (Fig. S2). We evaluated the enterotoxicity of these strains using the rabbit ileal loop test (Fig. 4). The WT strain showed significant enterotoxicity. This enterotoxicity was significantly reduced in the *∆hlyA∆vscV2* strain to a level similar to that in non-infected controls. These results indicate that the food-derived strain V130055 is capable of inducing diarrhoea and that T3SS2 plays a central role in this enterotoxicity.

## Discussion

Whole-genome sequencing has become increasingly used for molecular epidemiological analysis because of its high discrimination capacity compared to other methods, such as pulsed-field gel electrophoresis and multilocus variable-number tandem-repeat analysis [31,32]. Once the causative organism is identified, epidemiological links between sporadic cases and between isolates from foods and patients can be established with whole-genome information. However, the isolation and identification of the agent from non-sterile samples are time-consuming and labour-intensive. Furthermore, when multiple populations of bacteria are detected, the identification of the etiological agent becomes more difficult. In this study, several serogroups of *V. cholerae* were isolated from contaminated food and patients; however, the causative agent of the food poisoning could not be determined using conventional laboratory tests. Therefore, the analyses based on whole-genome sequencing have been used to support the identification of causative agents in addition to laboratory tests. In this study, T3SS that were present only in *V. cholerae* serogroup O144 were identified, and the enterotoxicity of O144 *V. cholerae* was confirmed. Several *V. cholerae* serogroup O144 strains were isolated in association with an unusual increase in the incidence of cholera-like diseases in Kolkata, India [33]. Culture supernatants from these strains evoked a cytotoxic effect on CHO and HeLa cells but were negative for genes encoding virulence factors such as *ctxA*, *zot*, ace and *tcpA*; genes representing the heat-labile toxin, heat-stable toxin and verotoxin of *Escherichia coli*; and various variants of these genes using PCR. Therefore, the virulence factors that contribute to cholera-like diseases remain unknown. The T3SS2 gene cluster identified in this study was also found in two Indian O144 strains from the public database. It is conserved among these strains and may be involved in their enteropathogenicity. The *Vibrio* T3SS2 was first discovered in *V. parahaemolyticus,* and it was later identified in non-O1, non-O139 *V. cholerae* [28]. Both these T3SS2 gene clusters are found on the genomic island and identified in bacteria genera other than *Vibrio*, which implies the T3SS2 gene cluster would be acquired through horizontal gene transfer events [27,29]. We previously reported that the non-O1, non-O139 *V. cholerae* strain RIMD2214243 (serogroup O5) has a T3SS2 gene cluster and that T3SS2 and its effector, VopM, are involved in enterotoxicity [25]. The V130055 strain also had VopM (identity: 97.4%) encoded within the T3SS2 gene cluster, indicating that VopM may be an effector protein responsible for T3SS2-mediated enterotoxicity of the V130055 strain.

The identification of the causative agents of foodborne diseases is important for public health. However, it should be noted that laboratory tests and molecular epidemiological analyses may not be the most effective methods for achieving this. The utilization of whole-genome data in a multifaceted manner, for example, in the search for virulence factors, can enhance the probability of identifying the causative agent. Furthermore, it has been proposed to establish a genomic database of pathogenic bacteria. This database would store the whole-genome data of various pathogens and be used to develop new methods for the rapid and accurate detection of the causative agents in foodborne outbreaks and the pathogens in the environment. This approach would help control the outbreak and assess the risk of infection in the future.

## supplementary material

10.1099/mgen.0.001364 Uncited Supplementary Material 1.

10.1099/mgen.0.001364 Uncited Supplementary Material 2.

## Abbreviations

CDSs: coding sequences
FRT: flippase recognition target
LB: Luria-Bertani
NCBI: National Center for Biotechnology Information
NIID: National Institute of Infectious Diseases
SNVs: single nucleotide variants
TDH: thermostable direct haemolysin
TRH: TDH-related haemolysin
T3SS: type III secretion system
UT: untypeable
