# Supplementary material for: Genomic and pathogenicity analyses to identify the causative agent from multiple serogroups of non-O1, non-O139 Vibrio cholerae in foodborne outbreaks
Source: Microb Genom. 2025 Feb 26;11(2):001364. doi: 10.1099/mgen.0.001364 (PMC11865499; doi:10.1099/mgen.0.001364)
Supplement: Uncited Supplementary Material 1. [file mgen-11-01364-s001.pdf]

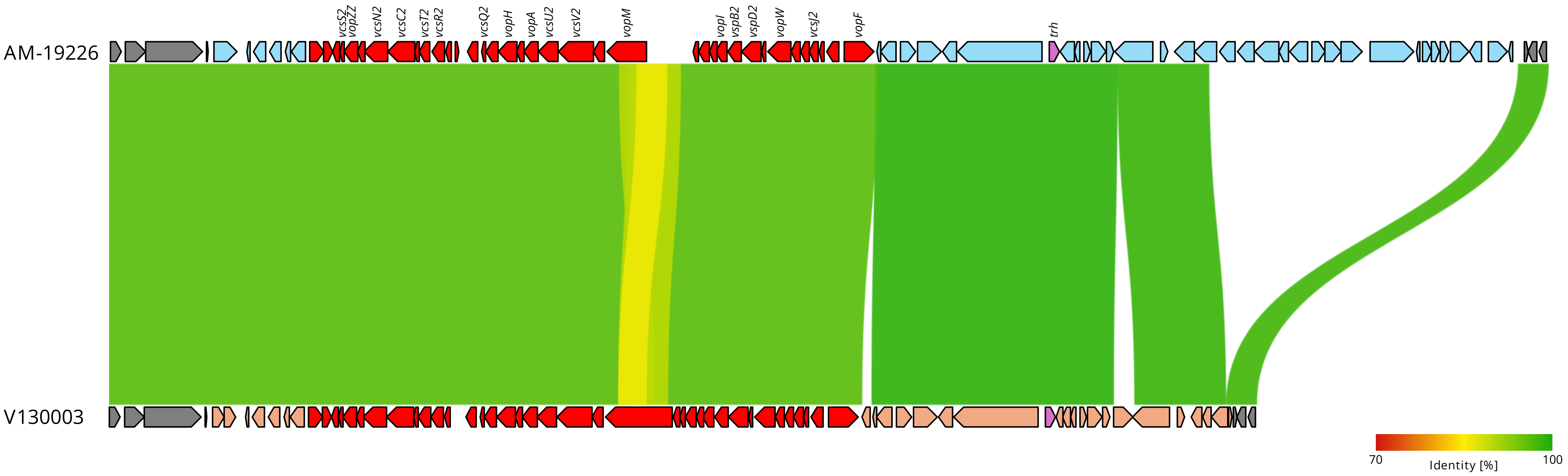


**Fig. S1.** Schematic diagrams of *V. cholerae* T3SS2α gene cluster. Alignment of genomic island with T3SS2 gene cluster was performed and visualized using AliTV v.1.0.6 (Ankenbrand MJ, Hohlfeld S, Hackl T, Förster F. AliTV—interactive visualization of whole genome comparisons. PeerJ Comput Sci 2017;3:e116). T3SS2 genes and *trh* gene were listed at the top. Genes on T3SS2 core region are shown in red. Genes conserved in *V. cholerae* are shown in grey.

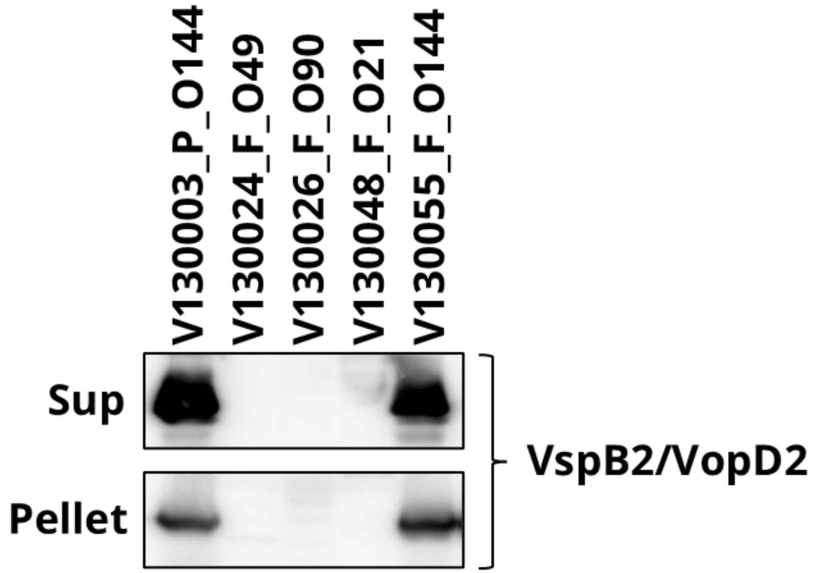


**Fig. S2.** Immunoblot analysis of VspB2/VopD2 in culture supernatants (Sup) and bacterial pellets (Pellet). The production and secretion of effector protein was observed in patient- and food-derived O144 strains, but not in strains of other serogroup strains without T3SS gene cluster.
