# Supplementary material for: Genomic and pathogenicity analyses to identify the causative agent from multiple serogroups of non-O1, non-O139 Vibrio cholerae in foodborne outbreaks
Source: Microb Genom. 2025 Feb 26;11(2):001364. doi: 10.1099/mgen.0.001364 (PMC11865499; doi:10.1099/mgen.0.001364)
Supplement: Uncited Supplementary Material 2. [file mgen-11-01364-s002.pdf]

Table S1. Non-O1, non-O139 *Vibrio cholerae* isolates from the foodborne outbreaks and summary statistics of genomic data

Sample ID	Prefecture	Source	Isolate name	Serogroup	Accession number	Number of contigs	Estimated genome size (bp)	Longest contig (bp)	N50 (bp)	GC contents (%)
P1	Oita	Patient	V130003	O144	DRR294493, DRR294518, AP024967, AP024968	2	3,070,742 and 1,072,820	-	-	47.58
P2	Oita	Patient	V130004	O144	DRR294494	100	4,035,798	227,521	109,171	47.55
P2	Oita	Patient	V130005	O144	-	-		-	-	-
P2	Oita	Patient	V130006	O144	-	-	-	-	-	-
P3	Oita	Patient	V130007	O144	DRR294495	97	4,035,270	210,468	106,270	47.55
P3	Oita	Patient	V130008	O144	-	-	-	-	-	-
P3	Oita	Patient	V130009	O144	-	-	-	-	-	-
P4	Oita	Patient	V130010	O144	-	-	-	-	-	-
P4	Oita	Patient	V130011	O144	-	-	-	-	-	-
P4	Oita	Patient	V130012	O144	-	-	-	-	-	-
P4	Oita	Patient	V130013	O144	-	-	-	-	-	-
P5	Oita	Patient	V130014	O144	DRR294496	120	4,036,087	210,392	106,270	47.55
P5	Oita	Patient	V130015	O144	-	-	-	-	-	-
P5	Oita	Patient	V130016	O144	-	-	-	-	-	-
P1	Oita	Patient	V130017	O144	-	-	-	-	-	-
P2	Oita	Patient	V130018	O144	-	-	-	-	-	-
P3	Oita	Patient	V130019	O144	-	-	-	-	-	-
P4	Oita	Patient	V130020	O144	-	-	-	-	-	-
P6	Oita	Patient	V130021	O144	DRR294497	120	4,038,471	210,392	104,009	47.55
P6	Oita	Patient	V130022	O144	-	-	-	-	-	-
P5	Oita	Patient	V130023	O144	-	-	-	-	-	-
F1	Oita	Food	V130024	O49	DRR294498	57	3,850,948	533,472	181,601	47.71
F1	Oita	Food	V130025	O49	-	-	-	-	-	-
F1	Oita	Food	V130026	O90	DRR294499	65	3,852,357	342,666	154,523	47.71
P7	Ishikawa	Patient	V130027	O144	DRR294500	121	4,044,947	377,685	102,990	47.58
P7	Ishikawa	Patient	V130028	O144	-	-	-	-	-	-
P8	Ishikawa	Patient	V130029	O176	DRR294501	79	3,835,869	402,437	159,064	47.65
P8	Ishikawa	Patient	V130030	O144	DRR294502	104	4,048,547	272,678	106,272	47.55
F2	Ishikawa	Food	V130031	O49	DRR294503	43	3,863,166	520,010	311,460	47.71
F1	Oita	Food	V130032	O49	-	-	-	-	-	-
F1	Oita	Food	V130033	O49	-	-	-	-	-	-
F1	Oita	Food	V130034	O49	-	-	-	-	-	-
F1	Oita	Food	V130035	O49	-	-	-	-	-	-
F3	Oita	Food	V130036	O192	-	-	-	-	-	-
F3	Oita	Food	V130037	O90	DRR294504	73	3,851,600	511,364	168,465	47.71
F3	Oita	Food	V130038	O145	DRR294505	103	4,040,522	375,157	144,405	47.56
F4	Oita	Food	V130039	O151	DRR294506	120	4,173,867	539,035	127,953	47.42
F5	Oita	Food	V130040	O21	DRR294507	88	4,189,503	354,573	142,315	47.38
F5	Oita	Food	V130041	O21	-	-	-	-	-	-
F5	Oita	Food	V130042	O21	-	-	-	-	-	-
F5	Oita	Food	V130043	O21	-	-	-	-	-	-
F5	Oita	Food	V130044	O128	DRR294508	83	3,858,569	236,985	148,506	47.77
F5	Oita	Food	V130045	O21	-	-	-	-	-	-
F5	Oita	Food	V130046	R	DRR294509	96	4,190,605	374,221	139,020	47.39
F6	Oita	Food	V130047	O176	DRR294510	78	3,819,434	402,779	150,050	47.66
F6	Oita	Food	V130048	O21	DRR294511	94	4,158,086	408,067	140,973	47.32
F6	Oita	Food	V130049	O21	-	-	-	-	-	-
F3	Oita	Food	V130050	O90	-	-	-	-	-	-
F3	Oita	Food	V130051	O90	-	-	-	-	-	-
F4	Oita	Food	V130052	O49	-	-	-	-	-	-

F5	Oita	Food	V130053	O49	-	-	-	-	-	-	
F5	Oita	Food	V130054	O145	-	-	-	-	-	-	
F6	Oita	Food	V130055	O144	DRR294512		98	4,039,169	210,348	106,270	47.55
F6	Oita	Food	V130056	O144	-	-	-	-	-	-	
F3	Oita	Food	V130057	O24	DRR294513		83	3,896,126	403,703	148,205	47.69
F6	Oita	Food	V130058	O186	DRR294514		72	3,875,914	514,675	165,915	47.74
P9	Oita	Patient	V130059	O144	DRR294515		140	4,039,576	201,725	70,952	47.63
P9	Oita	Patient	V130060	O144	-	-	-	-	-	-	
P9	Oita	Patient	V130061	O144	-	-	-	-	-	-	
P9	Oita	Patient	V130062	UT	-	-	-	-	-	-	
P9	Oita	Patient	V130063	UT	DRR294516		79	3,891,687	476,313	158,608	47.65
P10	Oita	Patient	V130064	O144	DRR294517		112	4,004,518	337,114	99,148	47.57
P10	Oita	Patient	V130065	O144	-	-	-	-	-	-	
P10	Oita	Patient	V130066	O144	-	-	-	-	-	-	

Table S2. List of core and accessory genes from the pan-genome profile

Gene	Annotation	No. isolates	Category
group_10	hybrid sensor histidine kinase/response regulator	25	core
group_100	methyl-accepting chemotaxis protein	25	core
group_1001	DEAD/DEAH box helicase	25	core
group_1003	ABC transporter ATP-binding protein	25	core
group_1004	TetR family transcriptional regulator	25	core
group_1005	AraC family transcriptional regulator	25	core
group_1006	branched-chain amino acid ABC transporter permease	25	core
group_1007	MarR family transcriptional regulator	25	core
group_1008	outer membrane protein	25	core
group_1009	glycogen operon protein GlgX homolog	25	core
cyaA	adenylate cyclase	25	core
group_1016	hypothetical protein	25	core
group_1017	HlyD family type I secretion periplasmic adaptor subunit	25	core
group_1018	purine-binding chemotaxis protein CheW	25	core
group_1019	Fis family transcriptional regulator	25	core
group_102	DTW domain-containing protein	25	core
group_1020	ABC transporter permease	25	core
group_1021	ABC transporter ATP-binding protein	25	core
group_1022	DNA-binding response regulator	25	core
group_1024	hypothetical protein	25	core
group_1025	phage shock protein G	25	core
group_1027	AraC family transcriptional regulator	25	core
lpxH	UDP-2,3-diacylglucosamine hydrolase	25	core
group_103	cell division ATP-binding protein FtsE	25	core
group_1034	agglutination protein	25	core
group_1037	molybdopterin-synthase adenylyltransferase MoeB	25	core
group_1039	sensor domain-containing phosphodiesterase	25	core
group_104	alkaline serine protease	25	core
nspS	Norspermidine sensor	25	core
group_1049	chitinase	25	core
bioH	pimeloyl-[acyl-carrier protein] methyl ester esterase	25	core
group_1062	thiazole biosynthesis adenylyltransferase ThiF	25	core
group_1069	peptidase	25	core
group_1070	peptidylprolyl isomerase	25	core
group_108	glutathione-regulated potassium-efflux system protein KefB	25	core
group_109	multidrug transporter	25	core
group_11	3-deoxy-D-manno-octulosonic acid transferase	25	core

group_110	hypothetical protein	25	core
thiP	thiamine/thiamine pyrophosphate ABC transporter permease ThiP	25	core
group_112	UPF0307 protein	25	core
group_1124	MSHA biogenesis protein MshG	25	core
group_1125	acetyltransferase	25	core
group_113	hypothetical protein	25	core
coaE	dephospho-CoA kinase	25	core
group_1155	long-chain-fatty-acid--CoA ligase	25	core
group_116	deoxyribonuclease	25	core
group_1166	hypothetical protein	25	core
copA	copper-exporting P-type ATPase A	25	core
vibF	nonribosomal peptide synthetase VibF	25	core
group_119	protease	25	core
group_12	methyl-accepting chemotaxis protein	25	core
group_123	hypothetical protein	25	core
group_124	SDR family oxidoreductase	25	core
group_125	paraquat-inducible protein A	25	core
group_126	amino acid transporter LysE	25	core
vcc	microbial collagenase	25	core
galM	aldose 1-epimerase	25	core
group_129	hypothetical protein	25	core
panE	2-dehydropantoate 2-reductase	25	core
group_131	TorD family cytoplasmic chaperone	25	core
group_134	GGDEF domain-containing protein	25	core
group_135	ABC transporter substrate-binding protein	25	core
group_136	2-keto-4-pentenoate hydratase	25	core
group_137	putative methylaconitate Delta-isomerase PrpF	25	core
group_138	3'-5' exonuclease	25	core
group_139	dehydrogenase	25	core
mnmC	tRNA 5-methylaminomethyl-2-thiouridine biosynthesis bifunctional protein MnmC	25	core
group_140	serine protease	25	core
group_141	phosphate ABC transporter substrate-binding protein	25	core
group_142	sensor histidine kinase	25	core
group_143	transcriptional regulator	25	core
group_144	CAAX amino protease	25	core
group_145	chemotaxis protein	25	core
group_148	hypothetical protein	25	core
group_15	hypothetical protein	25	core
truC	tRNA pseudouridine synthase C	25	core

group_151	hypothetical protein	25	core
group_154	Fe2+-enterobactin ABC transporter substrate-binding protein	25	core
group_155	hypothetical protein	25	core
group_156	MarR family transcriptional regulator	25	core
group_157	A/G-specific adenine glycosylase	25	core
dusA	tRNA-dihydrouridine(20/20a) synthase	25	core
group_16	type VI secretion protein VgrG	25	core
group_162	histidine kinase	25	core
group_163	N-acetyltransferase	25	core
group_164	oxidoreductase	25	core
group_165	frnE protein	25	core
group_166	hypothetical protein	25	core
group_167	L-xylulose 5-phosphate 3-epimerase	25	core
group_168	hypothetical protein	25	core
group_17	nicotinamide mononucleotide transporter	25	core
group_172	phosphofructokinase	25	core
group_173	ABC transporter substrate-binding protein	25	core
group_175	sulfurtransferase	25	core
group_176	hemolysin	25	core
group_177	hypothetical protein	25	core
group_178	hypothetical protein	25	core
group_179	membrane protein	25	core
group_18	hypothetical protein	25	core
glgC2	glucose-1-phosphate adenylyltransferase 2	25	core
group_181	peptidase	25	core
group_185	MBL fold metallo-hydrolase	25	core
group_186	3-methyladenine DNA glycosylase	25	core
group_187	sugar ABC transporter substrate-binding protein	25	core
group_188	methyl-accepting chemotaxis protein	25	core
group_189	two-component system sensor histidine kinase/response regulator	25	core
cheB1_2	chemotaxis response regulator protein-glutamate methylesterase of group 1 operon	25	core
group_191	chemotaxis protein CheA	25	core
group_192	hydrolase	25	core
group_2	C4-dicarboxylate ABC transporter	25	core
group_20	thiamine-phosphate synthase	25	core
gyrB	DNA gyrase subunit B	25	core
group_203	transcriptional regulator	25	core
group_204	MATE family efflux transporter	25	core
group_205	O-methyltransferase	25	core

engB	putative GTP-binding protein EngB	25	core
epsL	type II secretion system protein L	25	core
group_21	lactate dehydrogenase	25	core
ompR	DNA-binding response regulator	25	core
envZ	two-component sensor histidine kinase	25	core
group_212	hypothetical protein	25	core
argC	N-acetyl-gamma-glutamyl-phosphate reductase	25	core
group_215	hypothetical protein	25	core
thiQ	thiamine import ATP-binding protein ThiQ	25	core
group_217	DUF547 domain-containing protein	25	core
group_218	hypothetical protein	25	core
cca	multifunctional CCA protein	25	core
group_22	short-chain dehydrogenase	25	core
group_220	general secretion pathway protein GspB	25	core
tolC	outer membrane protein TolC	25	core
group_222	7,8-dihydro-8-oxoguanine-triphosphatase	25	core
dapB	4-hydroxy-tetrahydrodipicolinate reductase	25	core
group_225	aerobic respiration control sensor protein	25	core
group_226	hypothetical protein	25	core
group_227	hypothetical protein	25	core
group_228	phosphohydrolase	25	core
recD	RecBCD enzyme subunit RecD	25	core
group_23	cysteine desulfurase, sulfur acceptor subunit CsdE	25	core
proB	glutamate 5-kinase	25	core
group_231	hypothetical protein	25	core
group_232	hypothetical protein	25	core
prmC	release factor glutamine methyltransferase	25	core
group_234	deferrochelatase/peroxidase YfeX	25	core
fliP	flagellar biosynthetic protein FliP	25	core
group_236	elongation factor P hydroxylase	25	core
sucC	succinate--CoA ligase [ADP-forming] subunit beta	25	core
group_239	heme exporter protein B	25	core
group_24	4-methyl-5(B-hydroxyethyl)-thiazole monophosphate biosynthesis protein	25	core
group_240	histone deacetylase	25	core
group_241	hypothetical protein	25	core
group_242	thioesterase	25	core
group_243	GGDEF-domain containing protein	25	core
group_244	hypothetical protein	25	core
mfd	transcription-repair-coupling factor	25	core

group_248	methyl-accepting chemotaxis protein	25	core
group_249	methyl-accepting chemotaxis protein	25	core
group_251	hypothetical protein	25	core
group_256	hypothetical protein	25	core
group_257	hypothetical protein	25	core
mukB	chromosome partition protein MukB	25	core
group_259	zinc protease	25	core
rlmF	ribosomal RNA large subunit methyltransferase F	25	core
group_260	ABC transporter ATP-binding protein	25	core
group_261	multidrug transporter AcrB	25	core
group_263	RNA pseudouridine synthase	25	core
group_264	ABC transporter ATP-binding protein	25	core
group_265	sensory box sensor histidine kinase/response regulator VieS	25	core
group_266	methyl-accepting chemotaxis protein	25	core
group_267	MATE family efflux transporter	25	core
group_268	hydroxyneurosporene synthase	25	core
group_269	hypothetical protein	25	core
group_27	bifunctional molybdopterin-guanine dinucleotide biosynthesis protein MobB/molybdopterin molybdotransferase MoeA	25	core
metA	homoserine O-succinyltransferase	25	core
group_271	chemotaxis protein CheV	25	core
galK	galactokinase	25	core
group_273	UPF0753 protein	25	core
group_274	ATP-dependent DNA ligase	25	core
group_275	hypothetical protein	25	core
nagK	N-acetyl-D-glucosamine kinase	25	core
mobA	molybdenum cofactor guanylyltransferase	25	core
cobB	NAD-dependent protein deacylase	25	core
group_279	hypothetical protein	25	core
tppB	dipeptide/tripeptide permease A	25	core
group_281	hypothetical protein	25	core
cry2	cryptochrome-like protein cry2	25	core
maiA	putative maleylacetoacetate isomerase	25	core
group_29	polymerase	25	core
alr	alanine racemase	25	core
group_292	L-serine ammonia-lyase	25	core
group_293	sensor histidine kinase	25	core
group_294	hypothetical protein	25	core
btuD	vitamin B12 import ATP-binding protein BtuD	25	core
group_296	peptide-methionine (R)-S-oxide reductase	25	core

group_297	UPF0299 membrane protein	25	core
group_298	GGDEF domain-containing protein	25	core
hutG	formimidoylglutamase	25	core
volA	lysophospholipase VolA	25	core
group_300	molecular chaperone DnaJ	25	core
trpG	anthranilate synthase component 2	25	core
group_302	hypothetical protein	25	core
group_303	prepilin peptidase	25	core
group_304	hypothetical protein	25	core
group_305	phosphatase	25	core
group_306	two-component system response regulator	25	core
group_307	iron-sulfur cluster carrier protein	25	core
moaC	cyclic pyranopterin monophosphate synthase accessory protein	25	core
uvrB	excinuclease ABC subunit B	25	core
asnB	asparagine synthase B	25	core
lnt	apolipoprotein N-acyltransferase	25	core
group_313	hypothetical protein	25	core
group_318	hypothetical protein	25	core
tadA	tRNA-specific adenosine deaminase	25	core
group_32	serine-type D-Ala-D-Ala carboxypeptidase	25	core
dnaJ	chaperone protein DnaJ	25	core
oadA-2	oxaloacetate decarboxylase	25	core
queA	S-adenosylmethionine:tRNA ribosyltransferase-isomerase	25	core
clpB	chaperone protein ClpB	25	core
murJ	putative lipid II flippase MurJ	25	core
group_328	tRNA1(Val) (adenine(37)-N6)-methyltransferase	25	core
group_329	sensory box sensor histidine kinase/response regulator	25	core
rpoS	RNA polymerase sigma factor RpoS	25	core
mrcB	penicillin-binding protein 1B	25	core
group_331	LuxR family transcriptional regulator	25	core
rimM	ribosome maturation factor RimM	25	core
group_335	MSHA biogenesis protein MshN	25	core
group_336	alanine--glyoxylate aminotransferase	25	core
group_337	hypothetical protein	25	core
group_338	acyltransferase	25	core
dusB	tRNA-dihydrouridine synthase B	25	core
group_340	phosphogluconate dehydratase	25	core
group_347	glycosyl transferase	25	core
group_348	glycosyl transferase	25	core

group_349	ADP-heptose--LPS heptosyltransferase II	25	core
group_35	iron(III) ABC transporter substrate-binding protein	25	core
pyrE	orotate phosphoribosyltransferase	25	core
murP	PTS system N-acetylmuramic acid-specific EIIBC component	25	core
group_353	hypothetical protein	25	core
group_354	aminopeptidase	25	core
group_355	ATP-dependent DNA helicase RecQ	25	core
mnmG	tRNA uridine 5-carboxymethylaminomethyl modification enzyme MnmG	25	core
group_357	hypothetical protein	25	core
group_358	NadC family protein	25	core
group_359	tricorn protease	25	core
group_36	TagA-like protein	25	core
group_360	hypothetical protein	25	core
phrA	deoxyribodipyrimidine photo-lyase	25	core
group_362	oxidoreductase	25	core
group_363	type VI secretion-associated protein	25	core
group_364	N-ribosylnicotinamide CRP regulator	25	core
group_365	hydroxyglutarate oxidase	25	core
group_366	NADH dehydrogenase	25	core
group_367	hypothetical protein	25	core
group_369	hypothetical protein	25	core
group_37	hypothetical protein	25	core
group_370	methyl-accepting chemotaxis protein	25	core
group_371	nicotinate-nicotinamide nucleotide adenylyltransferase	25	core
lifO	lipase chaperone	25	core
group_373	hypothetical protein	25	core
group_374	ligand-gated channel	25	core
group_375	hypothetical protein	25	core
group_377	two-component sensor histidine kinase	25	core
ulaA_2	PTS ascorbate transporter subunit IIBC	25	core
group_379	methyl-accepting chemotaxis protein	25	core
group_380	integron integrase	25	core
group_388	bifunctional PTS fructose transporter subunit IIA/HPr protein	25	core
sbcC	nuclease SbcCD subunit C	25	core
group_39	D-3-phosphoglycerate dehydrogenase	25	core
cqsA	CAI-1 autoinducer synthase	25	core
group_392	9-hexadecenoic acid cis-trans isomerase	25	core
group_394	hypothetical protein	25	core
group_395	peptide ABC transporter ATP-binding protein	25	core

group_3984	TIGR03899 family protein	25	core
group_3985	RNA polymerase sigma factor RpoE	25	core
hemA	glutamyl-tRNA reductase	25	core
group_3987	chromosome partitioning protein ParA	25	core
group_3988	putative Fe(2+)-trafficking protein	25	core
atpF	ATP synthase subunit b	25	core
phnX	phosphonoacetaldehyde hydrolase	25	core
oadG1	putative oxaloacetate decarboxylase gamma chain 1	25	core
group_3991	uridine phosphorylase	25	core
pdhR	transcriptional regulator PdhR	25	core
group_3993	hypothetical protein	25	core
group_3994	hypothetical protein	25	core
group_3995	site-determining protein	25	core
group_3996	DNA-binding response regulator	25	core
group_3997	hypothetical protein	25	core
group_3998	hypothetical protein	25	core
group_3999	putative 4-hydroxy-4-methyl-2-oxoglutarate aldolase	25	core
group_400	nucleoside diphosphate kinase regulator	25	core
group_4000	flagellar motor switch protein FliN	25	core
accA	acetyl-coenzyme A carboxylase carboxyl transferase subunit alpha	25	core
group_4002	membrane protein	25	core
group_4003	hypothetical protein	25	core
group_4004	hypothetical protein	25	core
group_4005	hypothetical protein	25	core
rplB	50S ribosomal protein L2	25	core
group_4007	hypothetical protein	25	core
cspV	cold shock protein CspV	25	core
yajC	preprotein translocase subunit YajC	25	core
group_4010	glutaredoxin	25	core
group_4011	UPF0250 protein	25	core
group_4012	flagellar basal-body rod protein FlgG	25	core
group_4013	tRNA-Leu	25	core
group_4014	membrane protein	25	core
group_4015	nitrogen regulatory protein P-II	25	core
group_4016	NrdH-redoxin	25	core
ftsL	cell division protein FtsL	25	core
group_4018	flagellar protein	25	core
group_4019	1,4-dihydroxy-2-naphthoyl-CoA synthase	25	core
fhs	formate--tetrahydrofolate ligase	25	core

rplQ	50S ribosomal protein L17	25	core
group_4021	flagellar basal body rod protein FlgB	25	core
group_4022	tRNA-Trp	25	core
group_4023	aldehyde-alcohol dehydrogenase	25	core
group_4024	hypothetical protein	25	core
aroK	shikimate kinase	25	core
malG	maltose transport system permease protein MalG	25	core
group_4027	hypothetical protein	25	core
group_4028	hypothetical protein	25	core
group_4029	sodium:proton antiporter	25	core
group_403	glyoxalase	25	core
group_4030	hypothetical protein	25	core
rplJ	50S ribosomal protein L10	25	core
nqrD	Na(+)-translocating NADH-quinone reductase subunit D	25	core
group_4033	hypothetical protein	25	core
group_4034	hypothetical protein	25	core
rpsE	30S ribosomal protein S5	25	core
group_4036	hypothetical protein	25	core
seqA	negative modulator of initiation of replication	25	core
nagB	glucosamine-6-phosphate deaminase	25	core
rplD	50S ribosomal protein L4	25	core
group_404	ligand-gated channel	25	core
fadR	fatty acid metabolism regulator protein	25	core
group_4041	peptidyl-prolyl cis-trans isomerase	25	core
group_4042	hypothetical protein	25	core
group_4043	type VI secretion protein	25	core
secE	protein translocase subunit SecE	25	core
rplR	50S ribosomal protein L18	25	core
group_4046	flavodoxin	25	core
rpmA	50S ribosomal protein L27	25	core
prs	ribose-phosphate pyrophosphokinase	25	core
group_4049	transcriptional regulator HexR	25	core
group_405	acetyltransferase	25	core
group_4050	hypothetical protein	25	core
group_4051	multidrug resistance protein	25	core
infC	initiation factor IF3	25	core
group_4053	TIGR02808 family protein	25	core
group_4054	peptide ABC transporter ATP-binding protein	25	core
frr	ribosome-recycling factor	25	core

group_4056	UPF0114 protein	25	core
group_4057	fructose-1,6-bisphosphatase	25	core
dksA	RNA polymerase-binding transcription factor DksA	25	core
group_4059	hypothetical protein	25	core
group_406	aminoimidazole riboside kinase	25	core
rpmC	50S ribosomal protein L29	25	core
rpmF	50S ribosomal protein L32	25	core
group_4062	class II fructose-bisphosphate aldolase	25	core
rplL	50S ribosomal protein L7/L12	25	core
group_4064	tRNA-Arg	25	core
rhlB	ATP-dependent RNA helicase RhlB	25	core
group_4066	hypothetical protein	25	core
rpsJ	30S ribosomal protein S10	25	core
rplM	50S ribosomal protein L13	25	core
group_4069	hypothetical protein	25	core
group_407	membrane protein	25	core
nhaR	transcriptional activator protein NhaR	25	core
group_4071	Tol-Pal system subunit TolQ	25	core
group_4072	diguanylate cyclase	25	core
group_4073	heat-shock protein Hsp20	25	core
group_4074	cytochrome c-type protein	25	core
rpsL	30S ribosomal protein S12	25	core
infA	translation initiation factor IF-1	25	core
group_4077	phosphate transport regulator	25	core
rpsI	30S ribosomal protein S9	25	core
rpsT	30S ribosomal protein S20	25	core
group_408	carotenoid dehydrogenase	25	core
group_4080	acyl-CoA thioesterase	25	core
group_4081	iron-binding protein IscA	25	core
rplY	50S ribosomal protein L25	25	core
group_4083	tol-pal system-associated acyl-CoA thioesterase	25	core
group_4084	alkylphosphonate utilization protein	25	core
thrS	threonine--tRNA ligase	25	core
group_4086	antimicrobial peptide ABC transporter permease SapC	25	core
hflK	protein HflK	25	core
group_4088	peptide ABC transporter permease	25	core
group_4089	YcgL domain-containing protein	25	core
group_409	3'3'-cGAMP-specific phosphodiesterase 1	25	core
group_4090	UPF0434 protein	25	core

fliA	RNA polymerase sigma factor FliA	25	core
rpmG	50S ribosomal protein L33	25	core
group_4093	response regulator	25	core
group_4094	transcriptional regulator	25	core
group_4095	acetyl-CoA carboxylase biotin carboxyl carrier protein subunit	25	core
potA	spermidine/putrescine import ATP-binding protein PotA	25	core
group_4097	two-component system response regulator TorR	25	core
groS1	10 kDa chaperonin 1	25	core
pstB2	phosphate import ATP-binding protein PstB 2	25	core
group_410	guanylate cyclase	25	core
group_4100	tRNA-Pro	25	core
metJ	Met repressor	25	core
group_4102	cold-shock protein	25	core
group_4103	tRNA-Met	25	core
group_4104	PrkA family serine protein kinase	25	core
group_4105	oxygen-insensitive NAD(P)H-dependent nitroreductase NfsB	25	core
fusA1	elongation factor G 1	25	core
minE	cell division topological specificity factor	25	core
group_4108	transcriptional regulator AsnC	25	core
group_4109	succinate--CoA ligase [ADP-forming] subunit alpha	25	core
group_411	hypothetical protein	25	core
flaA	flagellin A	25	core
hupB	DNA-binding protein HU-beta	25	core
pta	phosphate acetyltransferase	25	core
rpsN	30S ribosomal protein S14	25	core
csrA	carbon storage regulator	25	core
prfB	peptide chain release factor 2	25	core
atpH	ATP synthase subunit delta	25	core
rplE	50S ribosomal protein L5	25	core
menA	1,4-dihydroxy-2-naphthoate octaprenyltransferase	25	core
rpsH	30S ribosomal protein S8	25	core
group_412	molybdate ABC transporter substrate-binding protein	25	core
group_4120	iron transporter FeoA	25	core
groS2	10 kDa chaperonin 2	25	core
group_4122	ABC transporter permease	25	core
group_4123	sodium-type flagellar protein MotX	25	core
rpsU	30S ribosomal protein S21	25	core
nrdR	transcriptional repressor NrdR	25	core
group_4126	hypothetical protein	25	core

pyrD	dihydroorotate dehydrogenase (quinone)	25	core
group_4128	RNA polymerase sigma factor	25	core
group_4129	transcriptional initiation protein Tat	25	core
group_413	hypothetical protein	25	core
group_4130	3-deoxy-D-manno-octulosonate 8-phosphate phosphatase	25	core
group_4131	ubiquinol-cytochrome c reductase iron-sulfur subunit	25	core
group_4132	transcriptional regulator	25	core
group_4133	ferredoxin	25	core
group_4134	histidine triad nucleotide-binding protein	25	core
group_4135	arabinose 5-phosphate isomerase	25	core
rplV	50S ribosomal protein L22	25	core
hflC	protein HflC	25	core
group_4138	NADH dehydrogenase	25	core
frdD	fumarate reductase subunit D	25	core
group_414	aquaporin	25	core
acpP	acyl carrier protein	25	core
group_4141	peptidase S41	25	core
group_4142	hypothetical protein	25	core
ftsB	cell division protein FtsB	25	core
tatA_2	Sec-independent protein translocase protein TatA	25	core
group_4145	UPF0312 protein	25	core
nqrE	Na(+)-translocating NADH-quinone reductase subunit E	25	core
group_4147	hypothetical protein	25	core
rpsO	30S ribosomal protein S15	25	core
pyrG	CTP synthase	25	core
yieM	hypothetical protein	25	core
group_4150	hypothetical protein	25	core
cysB	transcriptional regulator CysB	25	core
group_4152	DNA-binding response regulator	25	core
group_4153	hypothetical protein	25	core
group_4154	flagellar hook protein FlgE	25	core
group_4155	RNA polymerase subunit sigma	25	core
rplA	50S ribosomal protein L1	25	core
cheR1	chemotaxis protein methyltransferase 1	25	core
sspA	stringent starvation protein A	25	core
group_4159	hypothetical protein	25	core
group_416	cytochrome c554	25	core
uspB	universal stress protein B	25	core
rnhA	ribonuclease HI	25	core

rluB	ribosomal large subunit pseudouridine synthase B	25	core
group_4163	flagellar basal-body rod protein FlgC	25	core
fis	DNA-binding protein Fis	25	core
group_4165	chemotaxis protein CheX	25	core
tsf	elongation factor Ts	25	core
group_4167	hypothetical protein	25	core
group_4168	iron transporter FeoC	25	core
rpsD	30S ribosomal protein S4	25	core
group_417	ACP phosphodiesterase	25	core
ppa	inorganic pyrophosphatase	25	core
ribH	6,7-dimethyl-8-ribityllumazine synthase	25	core
nqrB	Na(+)-translocating NADH-quinone reductase subunit B	25	core
rpoZ	DNA-directed RNA polymerase subunit omega	25	core
fliG	flagellar motor switch protein FliG	25	core
group_4175	acyl-CoA thioesterase II	25	core
parB	putative chromosome-partitioning protein ParB	25	core
rbfA	ribosome-binding factor A	25	core
rplI	50S ribosomal protein L9	25	core
deoD1	purine nucleoside phosphorylase DeoD-type 1	25	core
group_418	UPF0271 protein	25	core
group_4180	LysR family transcriptional regulator	25	core
group_4181	tRNA-Phe	25	core
group_4182	DeoR/GlpR family transcriptional regulator	25	core
group_4183	hypothetical protein	25	core
group_4184	cell division protein ZapA	25	core
exbD2	biopolymer transport protein exbD2	25	core
group_4186	putative intracellular septation protein A	25	core
group_4187	periplasmic nitrate reductase cytochrome c-type protein	25	core
rplN	50S ribosomal protein L14	25	core
rlmB	23S rRNA (guanosine-2'-O-)-methyltransferase RlmB	25	core
group_419	hypothetical protein	25	core
group_4190	bacteriocin production protein	25	core
group_4191	hypothetical protein	25	core
group_4192	hypothetical protein	25	core
msrB	peptide methionine sulfoxide reductase MsrB	25	core
rpsF	30S ribosomal protein S6	25	core
zapB	cell division protein ZapB	25	core
nusB	N utilization substance protein B	25	core
group_4197	hemolysin III family protein	25	core

group_4198	DNA-binding response regulator	25	core
atpG	ATP synthase gamma chain	25	core
group_42	AraC family transcriptional regulator	25	core
group_420	inosine/guanosine kinase	25	core
rplT	50S ribosomal protein L20	25	core
group_4201	hypothetical protein	25	core
npr	phosphocarrier protein NPr	25	core
group_4203	thioredoxin	25	core
mioC	protein MioC	25	core
pspB	phage shock protein B	25	core
group_4206	hypothetical protein	25	core
group_4207	inner membrane protein YpjD	25	core
group_4208	ABC transporter ATP-binding protein	25	core
gmhA_1	phosphoheptose isomerase	25	core
gbpA	hypothetical protein	25	core
group_4210	cytochrome c biogenesis protein CcsB	25	core
group_4211	outer membrane lipid asymmetry maintenance protein MlaD	25	core
rplU	50S ribosomal protein L21	25	core
group_4213	N-acetylglucosamine repressor	25	core
nutA	5'-nucleotidase	25	core
group_4215	aspartate ammonia-lyase	25	core
rpsK	30S ribosomal protein S11	25	core
group_4217	DNA-binding transcriptional regulator CytR	25	core
group_4218	tRNA-Ser	25	core
rpsG	30S ribosomal protein S7	25	core
group_422	methyltransferase	25	core
ftsA	cell division protein FtsA	25	core
group_4221	membrane protein	25	core
groL1	60 kDa chaperonin 1	25	core
group_4223	LysR family transcriptional regulator	25	core
group_4224	bacterioferritin	25	core
upp	uracil phosphoribosyltransferase	25	core
ihfB	integration host factor subunit beta	25	core
group_4227	hypothetical protein	25	core
group_4228	hypothetical protein	25	core
rpsC	30S ribosomal protein S3	25	core
group_4230	TetR family transcriptional regulator	25	core
group_4231	hypothetical protein	25	core
trkA	Trk system potassium transport protein TrkA	25	core

group_4233	PTS IIA-like nitrogen-regulatory protein PtsN	25	core
hupA	DNA-binding protein HU-alpha	25	core
group_4235	tRNA-Asn	25	core
ilvH	acetolactate synthase small subunit	25	core
group_4237	hypothetical protein	25	core
rpsB	30S ribosomal protein S2	25	core
rpsR	30S ribosomal protein S18	25	core
hap	hemagglutinin/proteinase	25	core
group_4240	UPF0056 inner membrane protein	25	core
rplP	50S ribosomal protein L16	25	core
fliS	flagellar protein FliS	25	core
ptsH	phosphocarrier protein HPr	25	core
group_4244	rhodanese-like domain-containing protein	25	core
cysE	serine O-acetyltransferase	25	core
fliQ	flagellar biosynthetic protein FliQ	25	core
group_4247	UPF0227 protein	25	core
group_4248	cytochrome-c oxidase	25	core
group_4249	protein phosphatase CheZ	25	core
ompW	outer membrane protein W	25	core
group_4250	hypothetical protein	25	core
group_4251	CDP-diacylglycerol--glycerol-3-phosphate 3-phosphatidyltransferase	25	core
group_4252	sulfur carrier protein ThiS	25	core
oppB	peptide ABC transporter permease	25	core
pyrH	uridylate kinase	25	core
cspA	cold shock-like protein CspA	25	core
group_4256	UPF0301 protein	25	core
group_4257	hypothetical protein	25	core
grx	glutaredoxin	25	core
argR	arginine repressor	25	core
group_4260	D-alanyl-D-alanine carboxypeptidase	25	core
group_4261	cation:proton antiporter	25	core
group_4262	flagellar biosynthesis anti-sigma factor FlgM	25	core
clpS	ATP-dependent Clp protease adapter protein ClpS	25	core
group_4264	hypothetical protein	25	core
group_4265	UPF0265 protein	25	core
group_4266	hypothetical protein	25	core
group_4267	ABC transporter	25	core
group_4268	nitrogen regulatory protein P-II 1	25	core
mgsA	methylglyoxal synthase	25	core

group_4270	D-3-phosphoglycerate dehydrogenase	25	core
group_4271	hypothetical protein	25	core
gpt	xanthine phosphoribosyltransferase	25	core
rpmE2	50S ribosomal protein L31 type B	25	core
rpmE	50S ribosomal protein L31	25	core
group_4275	peptidoglycan-associated lipoprotein	25	core
gph	phosphoglycolate phosphatase	25	core
group_4277	hypothetical protein	25	core
rpsP	30S ribosomal protein S16	25	core
group_4279	signal peptidase I	25	core
zwf	glucose-6-phosphate 1-dehydrogenase	25	core
ndk	nucleoside diphosphate kinase	25	core
atpE	ATP synthase subunit c	25	core
group_4282	hypothetical protein	25	core
group_4283	membrane protein	25	core
nusG	transcription termination/antitermination protein NusG	25	core
group_4285	membrane protein	25	core
group_4286	hypothetical protein	25	core
group_4287	hypothetical protein	25	core
group_4288	heme utilization protein HutZ	25	core
group_4289	hypothetical protein	25	core
group_429	hypothetical protein	25	core
group_4290	nucleoid-associated protein	25	core
rplW	50S ribosomal protein L23	25	core
group_4292	peptide ABC transporter permease	25	core
lapB	lipopolysaccharide assembly protein B	25	core
group_4294	acyl-CoA thioesterase	25	core
lpxA	acyl-[acyl-carrier-protein]--UDP-N-acetylglucosam ine O-acyltransferase	25	core
hflD	high frequency lysogenization protein HflD	25	core
fabA	3-hydroxydecanoyl-[acyl-carrier-protein] dehydratase	25	core
group_4298	chemotaxis protein CheW	25	core
group_4299	hypothetical protein	25	core
group_43	methyl-accepting chemotaxis protein	25	core
group_430	glycerate kinase	25	core
group_4300	glyceraldehyde-3-phosphate dehydrogenase	25	core
group_4301	transcriptional regulator	25	core
group_4302	ribosomal subunit interface protein	25	core
group_4303	multidrug DMT transporter	25	core
group_4304	biopolymer transporter TolR	25	core

group_4305	peptidase	25	core
group_4306	hypothetical protein	25	core
rpsQ	30S ribosomal protein S17	25	core
group_4308	UPF0253 protein	25	core
group_4309	Cys-tRNA(Pro)/Cys-tRNA(Cys) deacylase	25	core
group_431	hypothetical protein	25	core
lysS	lysine--tRNA ligase	25	core
motB	flagellar motor protein MotB	25	core
rpsS	30S ribosomal protein S19	25	core
group_4313	MSHA biogenesis protein MshK	25	core
group_4314	ABC transporter permease	25	core
group_4315	DUF3971 domain-containing protein	25	core
group_4316	smp protein	25	core
bolA	DNA-binding transcriptional regulator BolA	25	core
gltB	glutamate synthase large subunit	25	core
argB	acetylglutamate kinase	25	core
group_432	hypothetical protein	25	core
grcA	autonomous glycyl radical cofactor	25	core
group_4321	FAD-binding oxidoreductase	25	core
group_4322	GNAT family N-acetyltransferase	25	core
dapA	4-hydroxy-tetrahydrodipicolinate synthase	25	core
group_4324	branched chain amino acid aminotransferase	25	core
ileS	isoleucine--tRNA ligase	25	core
group_4326	ribonucleoside-diphosphate reductase	25	core
group_4327	zinc ABC transporter permease	25	core
nqrF	Na(+)-translocating NADH-quinone reductase subunit F	25	core
cysJ	sulfite reductase [NADPH] flavoprotein alpha-component	25	core
group_433	hypothetical protein	25	core
group_4330	peptide ABC transporter permease	25	core
group_4331	riboflavin biosynthesis protein	25	core
metH	methionine synthase	25	core
ackA2	acetate kinase 2	25	core
tolB	protein TolB	25	core
tatB	sec-independent protein translocase protein TatB	25	core
group_4336	peptidoglycan-binding protein LysM	25	core
hisD	histidinol dehydrogenase	25	core
nagA	N-acetylglucosamine-6-phosphate deacetylase	25	core
group_4339	sulfate ABC transporter permease	25	core
group_434	hypothetical protein	25	core

luxS	S-ribosylhomocysteine lyase	25	core
aat	leucyl/phenylalanyl-tRNA--protein transferase	25	core
glgC1	glucose-1-phosphate adenylyltransferase 1	25	core
group_4343	HxlR family transcriptional regulator	25	core
fabG	3-oxoacyl-[acyl-carrier-protein] reductase FabG	25	core
group_4345	ATPase AAA	25	core
group_4346	protein TolA	25	core
group_4347	hypothetical protein	25	core
group_4348	glyoxalase	25	core
grpE	protein GrpE	25	core
group_435	MFS transporter	25	core
group_4350	hypothetical protein	25	core
group_4352	hemolysin	25	core
tig	trigger factor	25	core
group_4354	peptidase	25	core
trxB	thioredoxin reductase	25	core
group_4356	coenzyme A pyrophosphatase	25	core
accD	acetyl-coenzyme A carboxylase carboxyl transferase subunit beta	25	core
mukE	chromosome partition protein MukE	25	core
slyD	FKBP-type peptidyl-prolyl cis-trans isomerase SlyD	25	core
group_436	ABC transporter	25	core
group_4360	TetR family transcriptional regulator	25	core
group_4361	hypothetical protein	25	core
group_4362	hypothetical protein	25	core
flhA	flagellar biosynthesis protein FlhA	25	core
group_4364	protein TonB	25	core
glpT	sn-glycerol-3-phosphate transporter	25	core
group_4366	class I poly(R)-hydroxyalkanoic acid synthase	25	core
mbaA	biofilm architecture maintenance protein MbaA	25	core
carB	carbamoyl-phosphate synthase large chain	25	core
group_4369	type VI secretion protein	25	core
group_437	amidinotransferase	25	core
group_4370	non-canonical purine NTP phosphatase	25	core
ung	uracil-DNA glycosylase	25	core
tpiA	triosephosphate isomerase	25	core
rimK	putative alpha-L-glutamate ligase	25	core
lipA_2	lipoyl synthase	25	core
gltD_1	dihydropyrimidine dehydrogenase subunit A	25	core
group_4376	hypothetical protein	25	core

cmoB	tRNA U34 carboxymethyltransferase	25	core
purF	amidophosphoribosyltransferase	25	core
group_4379	hypothetical protein	25	core
group_438	hypothetical protein	25	core
group_4380	nucleotidyltransferase	25	core
group_4381	MSHA biogenesis protein MshI	25	core
group_4382	cryptochrome/photolyase family protein	25	core
epd	D-erythrose-4-phosphate dehydrogenase	25	core
group_4384	hypothetical protein	25	core
group_4385	chemotaxis protein CheW	25	core
group_4386	cell shape-determining protein MreC	25	core
opgG	glucans biosynthesis protein G	25	core
dcuC	putative anaerobic C4-dicarboxylate transporter DcuC	25	core
group_4389	alpha-acetolactate decarboxylase	25	core
group_4390	AsnC family transcriptional regulator	25	core
recA	recombinase A	25	core
group_4392	peptide ABC transporter permease	25	core
group_4393	peptidyl-prolyl cis-trans isomerase	25	core
group_4394	alpha,alpha-phosphotrehalase	25	core
group_4395	2-keto-4-pentenoate hydratase	25	core
group_4396	pyruvate formate-lyase-activating enzyme	25	core
group_4397	peroxiredoxin	25	core
group_4398	oxidative stress defense protein	25	core
group_4399	putative phosphoenolpyruvate synthase regulatory protein	25	core
group_44	UPF0225 protein	25	core
group_4400	peptidyl-prolyl cis-trans isomerase	25	core
group_4401	LysR family transcriptional regulator	25	core
group_4402	DNA-binding response regulator	25	core
ispF	2-C-methyl-D-erythritol 2,4-cyclodiphosphate synthase	25	core
group_4404	membrane protein	25	core
prfA	peptide chain release factor 1	25	core
group_4406	peptidyl-prolyl cis-trans isomerase	25	core
group_4407	cytochrome c	25	core
group_4408	MFS transporter	25	core
proQ	RNA chaperone ProQ	25	core
group_4410	lipoprotein	25	core
group_4411	ABC transporter ATP-binding protein	25	core
dxr	1-deoxy-D-xylulose 5-phosphate reductoisomerase	25	core
rluA	ribosomal large subunit pseudouridine synthase A	25	core

rpoC	DNA-directed RNA polymerase subunit beta'	25	core
group_4415	translation elongation factor	25	core
smg	protein Smg	25	core
ratA	ribosome association toxin RatA	25	core
group_4418	bifunctional metallophosphatase/5'-nucleotidase	25	core
group_4419	cysteine synthase	25	core
group_442	sulfate adenylyltransferase	25	core
acsA	acetyl-coenzyme A synthetase	25	core
pepN	aminopeptidase N	25	core
group_4422	FMN reductase	25	core
group_4423	peptide ABC transporter permease	25	core
nudC	NADH pyrophosphatase	25	core
cpxA	two-component sensor histidine kinase	25	core
group_4426	hypothetical protein	25	core
argF	ornithine carbamoyltransferase	25	core
group_4428	ABC transporter permease	25	core
group_4429	ATP-dependent protease	25	core
tmcA	tRNA(Met) cytidine acetyltransferase TmcA	25	core
group_4430	tripartite tricarboxylate transporter TctA	25	core
lldD	L-lactate dehydrogenase	25	core
group_4432	replicative DNA helicase	25	core
group_4433	galactose ABC transporter substrate-binding protein	25	core
group_4434	ABC transporter ATP-binding protein	25	core
potC	spermidine/putrescine ABC transporter permease PotC	25	core
group_4436	cysteine synthase	25	core
clpA	ATP-dependent Clp protease ATP-binding subunit ClpA	25	core
nudE	ADP compounds hydrolase NudE	25	core
mltA	membrane-bound lytic murein transglycosylase A	25	core
group_444	arginase	25	core
group_4440	DNA-binding response regulator	25	core
group_4441	fimbrial protein	25	core
group_4442	hypothetical protein	25	core
group_4443	redox-sensitive transcriptional activator SoxR	25	core
group_4444	threonine transporter RhtB	25	core
group_4445	hypothetical protein	25	core
group_4446	aspartate kinase	25	core
group_4447	pilus (MSHA type) biogenesis protein MshL	25	core
purC	phosphoribosylaminoimidazole-succinocarboxamide synthase	25	core
group_4449	N-acetylmuramoyl-L-alanine amidase	25	core

rbsD	D-ribose pyranase	25	core
group_4450	ABC transporter permease	25	core
purA	adenylosuccinate synthetase	25	core
group_4452	purine-binding chemotaxis protein CheW	25	core
group_4453	aminobenzoyl-glutamate transporter	25	core
group_4454	ABC transporter substrate-binding protein	25	core
group_4455	ribosomal subunit interface protein	25	core
group_4456	transcriptional regulator	25	core
group_4457	serine transporter	25	core
group_4458	hypothetical protein	25	core
leuS	leucine--tRNA ligase	25	core
group_446	prepilin peptidase	25	core
group_4460	N-acetyltransferase	25	core
group_4461	transcriptional regulator	25	core
group_4462	molecular chaperone	25	core
group_4463	pyruvate kinase	25	core
epsE	type II secretion system protein E	25	core
group_4465	ligand-gated channel	25	core
pdxJ	pyridoxine 5'-phosphate synthase	25	core
group_4467	methionine ABC transporter ATP-binding protein	25	core
group_4468	beta-N-acetylhexosaminidase	25	core
group_4469	electron transport complex subunit D	25	core
group_4470	peptide ABC transporter ATP-binding protein	25	core
cadB	arginine:agmatine antiporter	25	core
group_4472	sigma-54-dependent Fis family transcriptional regulator	25	core
yihI	Der GTPase-activating protein YihI	25	core
ppk	polyphosphate kinase	25	core
btuC	vitamin B12 import system permease protein BtuC	25	core
group_4476	hypothetical protein	25	core
group_4477	tRNA-Leu	25	core
group_4478	tRNA-Asn	25	core
dinB	DNA polymerase IV	25	core
group_4480	UPF0145 protein	25	core
group_4481	chemotaxis protein CheW	25	core
mglC	galactoside ABC transporter permease MglC	25	core
pstB1	phosphate import ATP-binding protein PstB 1	25	core
group_4484	murein hydrolase effector protein LrgB	25	core
group_4485	hypothetical protein	25	core
group_4486	D-alanyl-D-alanine carboxypeptidase	25	core

group_4487	GGDEF domain-containing protein	25	core
flhF	flagellar biosynthesis regulator FlhF	25	core
group_4489	transcriptional regulator	25	core
group_449	hypothetical protein	25	core
group_4490	ABC transporter permease	25	core
group_4491	DNA-binding protein	25	core
glnD	bifunctional uridylyltransferase/uridylyl-removing enzyme	25	core
group_4493	6-phosphogluconate dehydrogenase, decarboxylating	25	core
serC	phosphoserine aminotransferase	25	core
group_4495	penicillin-binding protein activator LpoB	25	core
group_4496	hypothetical protein	25	core
group_4497	long-chain-fatty-acid--CoA ligase	25	core
group_4498	hypothetical protein	25	core
group_4499	hypothetical protein	25	core
group_450	DNA-3-methyladenine glycosylase I	25	core
group_4500	ATP-dependent RNA helicase RhlE	25	core
group_4501	threonine transporter RhtB	25	core
flgJ	peptidoglycan hydrolase FlgJ	25	core
group_4503	fimbrial protein	25	core
secD_2	protein translocase subunit SecD	25	core
fur	ferric uptake regulation protein	25	core
rraB	regulator of ribonuclease activity B	25	core
group_4507	PTS glucose transporter subunit IIA	25	core
group_4508	hypothetical protein	25	core
group_4509	UDP-glucose 6-dehydrogenase	25	core
group_4510	PAS domain-containing sensor histidine kinase	25	core
group_4511	lactate dehydrogenase	25	core
group_4512	transcriptional regulator	25	core
group_4513	ketohydroxyglutarate aldolase	25	core
ilvC	ketol-acid reductoisomerase (NADP(+))	25	core
rplX	50S ribosomal protein L24	25	core
group_4516	lipase	25	core
fadA	3-ketoacyl-CoA thiolase	25	core
group_4518	ABC transporter substrate-binding protein	25	core
guaB	inosine-5'-monophosphate dehydrogenase	25	core
group_4520	hypothetical protein	25	core
metE	5-methyltetrahydropteroyltriglutamate--homocystei ne methyltransferase	25	core
group_4522	tRNA-Gly	25	core
group_4523	cytochrome C biogenesis protein CcmI	25	core

epsI	type II secretion system protein I	25	core
group_4525	DNA-binding transcriptional regulator FabR	25	core
group_4526	hypothetical protein	25	core
nhaB	Na(+)/H(+) antiporter NhaB	25	core
group_4528	GGDEF domain-containing protein	25	core
group_4529	LysR family transcriptional regulator	25	core
group_4530	nucleotide-binding protein	25	core
rapA	RNA polymerase-associated protein RapA	25	core
leuO	transcriptional regulator LeuO	25	core
group_4533	preprotein translocase subunit Tim44	25	core
group_4534	hypothetical protein	25	core
group_4535	PTS sugar transporter subunit IIB	25	core
gyrA	DNA gyrase subunit A	25	core
nadE	NH(3)-dependent NAD(+) synthetase	25	core
group_4538	ribosomal large subunit pseudouridine synthase A	25	core
murD	UDP-N-acetylmuramoyl-L-alanyl-D-glutamate synthetase	25	core
group_4540	putrescine-binding periplasmic protein	25	core
fliD	flagellar hook-associated protein 2	25	core
group_4542	nucleoside permease	25	core
der	GTPase Der	25	core
group_4544	glutathione-disulfide reductase	25	core
group_4545	AI-2E family transporter	25	core
mraY	phospho-N-acetylmuramoyl-pentapeptide-transferase	25	core
znuC	zinc import ATP-binding protein ZnuC	25	core
group_4548	succinate dehydrogenase iron-sulfur subunit	25	core
group_4549	sensor histidine kinase	25	core
group_455	GGDEF domain-containing protein	25	core
epmA	elongation factor P--(R)-beta-lysine ligase	25	core
group_4551	type VI secretion system-associated protein	25	core
oadB	putative oxaloacetate decarboxylase beta chain	25	core
group_4553	ABC transporter	25	core
group_4554	citrate synthase	25	core
group_4555	DOPA 4,5-dioxygenase	25	core
group_4556	MSHA biogenesis protein MshE	25	core
group_4557	hypothetical protein	25	core
ispG	4-hydroxy-3-methylbut-2-en-1-yl diphosphate synthase (flavodoxin)	25	core
group_4559	membrane protein	25	core
group_4560	sulfate ABC transporter permease	25	core
gloB	hydroxyacylglutathione hydrolase	25	core

dam	DNA adenine methylase	25	core
group_4563	hemin ABC transporter substrate-binding protein	25	core
group_4564	isocitrate dehydrogenase, NADP-dependent	25	core
group_4565	superoxide dismutase	25	core
dctP	C4-dicarboxylate-binding periplasmic protein DctP	25	core
group_4567	coproporphyrinogen III oxidase	25	core
group_4568	short chain dehydrogenase	25	core
group_4569	sensor histidine kinase	25	core
group_4570	putative response regulatory protein	25	core
rlmJ	ribosomal RNA large subunit methyltransferase J	25	core
pfkA	ATP-dependent 6-phosphofructokinase	25	core
group_4573	MATE family efflux transporter	25	core
group_4574	peptidase M23	25	core
group_4575	manganese-dependent inorganic pyrophosphatase	25	core
group_4576	energy-dependent translational throttle protein EttA	25	core
group_4578	amino acid ABC transporter substrate-binding protein	25	core
thiL	thiamine-monophosphate kinase	25	core
group_4580	sulfate permease	25	core
flgH	flagellar L-ring protein	25	core
group_4582	ATP-binding protein	25	core
group_4583	phosphate transport system permease protein PstA	25	core
group_4584	type VI secretion system-associated protein	25	core
group_4585	LysR family transcriptional regulator	25	core
group_4586	rod shape-determining protein	25	core
group_4587	epimerase	25	core
gpsA	glycerol-3-phosphate dehydrogenase [NAD(P)+]	25	core
group_4589	paraquat-inducible protein B	25	core
group_4590	transporter	25	core
group_4591	SM-20	25	core
atpA	ATP synthase subunit alpha	25	core
parC	DNA topoisomerase 4 subunit A	25	core
group_4594	methylated-DNA--protein-cysteine methyltransferase	25	core
iscR	HTH-type transcriptional regulator IscR	25	core
phoR	PAS domain-containing sensor histidine kinase	25	core
lspA	lipoprotein signal peptidase	25	core
group_4598	anti-sigma B factor antagonist	25	core
group_4599	transcriptional regulator Crp	25	core
group_46	ribonuclease	25	core
rplS	50S ribosomal protein L19	25	core

group_4601	GGDEF domain-containing protein	25	core
fadI	3-ketoacyl-CoA thiolase	25	core
group_4603	ABC transporter ATP-binding protein	25	core
group_4604	adenine permease	25	core
plsY	glycerol-3-phosphate acyltransferase	25	core
fabH2	3-oxoacyl-[acyl-carrier-protein] synthase 3 protein 2	25	core
group_4607	LysR family transcriptional regulator	25	core
tnaA	tryptophanase	25	core
group_4609	phosphatidate cytidylyltransferase	25	core
ftsH	ATP-dependent zinc metalloprotease FtsH	25	core
group_4611	quinone oxidoreductase	25	core
group_4612	membrane protein	25	core
group_4613	7,8-dihydroneopterin aldolase	25	core
group_4614	histidine/lysine/arginine/ornithine ABC transporter ATP-binding protein HisP	25	core
group_4615	hemolysin	25	core
group_4616	oxaloacetate decarboxylase subunit beta	25	core
group_4617	tol-pal system protein YbgF	25	core
ate	putative arginyl-tRNA--protein transferase	25	core
rplO	50S ribosomal protein L15	25	core
group_462	methyl-accepting chemotaxis protein	25	core
group_4620	TIGR02444 family protein	25	core
group_4621	PTS trehalose transporter subunit IIBC	25	core
group_4622	arginine/ornithine succinyltransferase	25	core
group_4623	succinate dehydrogenase hydrophobic membrane anchor subunit	25	core
nspC	carboxynorspermidine/carboxyspermidine decarboxylase	25	core
group_4625	class II glutamine amidotransferase	25	core
guaC	GMP reductase	25	core
corC	magnesium and cobalt efflux protein CorC	25	core
group_4628	peptide ABC transporter substrate-binding protein	25	core
group_4629	magnesium transporter MgtE	25	core
lon	Lon protease	25	core
group_4631	UPF0016 family membrane protein	25	core
group_4632	putative phosphatase	25	core
alr1	alanine racemase 1	25	core
oppD	peptide ABC transporter ATP-binding protein	25	core
group_4635	peptidase	25	core
group_4636	glutamate decarboxylase	25	core
group_4637	delta-aminolevulinic acid dehydratase	25	core
group_4638	transcriptional regulator	25	core

group_4639	sodium-dependent transporter	25	core
group_464	N-acetyltransferase	25	core
murQ2	N-acetylmuramic acid 6-phosphate etherase 2	25	core
group_4641	DEAD/DEAH box helicase	25	core
skp	chaperone protein Skp	25	core
hisI	histidine biosynthesis bifunctional protein HisIE	25	core
pcm	protein-L-isoaspartate O-methyltransferase	25	core
flgL	flagellar hook-associated protein FlgL	25	core
group_4646	2-amino-4-hydroxy-6-hydroxymethyldihydropteridine diphosphokinase	25	core
fabV1	enoyl-[acyl-carrier-protein] reductase [NADH] 1	25	core
nth	endonuclease III	25	core
group_4649	3'(2'),5'-bisphosphate nucleotidase CysQ	25	core
group_465	hypothetical protein	25	core
group_4650	membrane protein	25	core
group_4651	ABC transporter ATP-binding protein	25	core
group_4652	GTP-dependent nucleic acid-binding protein EngD	25	core
cobS	adenosylcobinamide-GDP ribazoletransferase	25	core
murA	UDP-N-acetylglucosamine 1-carboxyvinyltransferase	25	core
recF	DNA replication and repair protein RecF	25	core
group_4656	transcriptional regulator	25	core
group_4657	mechanosensitive ion channel protein	25	core
group_4658	ribosomal RNA small subunit methyltransferase E	25	core
group_4659	TetR family transcriptional regulator	25	core
group_4660	spermidine/putrescine ABC transporter substrate-binding protein	25	core
group_4661	transcriptional regulator	25	core
glnA	glutamine synthetase	25	core
fliE	flagellar hook-basal body protein FliE	25	core
zapE	cell division protein ZapE	25	core
group_4665	tryptophan-specific transporter	25	core
group_4666	lipoprotein transporter subunit LolE	25	core
group_4667	LPS export ABC transporter permease LptG	25	core
alaS	alanine--tRNA ligase	25	core
group_4669	cytochrome bd oxidase subunit I	25	core
nnr	bifunctional NAD(P)H-hydrate repair enzyme Nnr	25	core
aroQ	3-dehydroquinate dehydratase	25	core
group_4671	reactive intermediate/imine deaminase	25	core
group_4672	MexH family multidrug efflux RND transporter periplasmic adaptor subunit	25	core
miaB	tRNA-2-methylthio-N(6)-dimethylallyladenosine synthase	25	core
group_4674	phosphoenolpyruvate--protein phosphotransferase PtsP	25	core

group_4675	DNA-binding protein	25	core
fadE	acyl-CoA dehydrogenase	25	core
folD	bifunctional protein FolD	25	core
dapD	2,3,4,5-tetrahydropyridine-2,6-dicarboxylate N-succinyltransferase	25	core
group_4679	sodium-independent anion transporter	25	core
group_4680	hypothetical protein	25	core
vibC	vibriobactin-specific isochorismate synthase	25	core
group_4682	methyl-accepting chemotaxis protein	25	core
cmoA	carboxy-S-adenosyl-L-methionine synthase	25	core
dtd	D-aminoacyl-tRNA deacylase	25	core
group_4685	UPF0721 transmembrane protein	25	core
group_4686	sodium-independent anion transporter	25	core
group_4687	GGDEF-domain containing protein	25	core
group_4688	ATPase AAA	25	core
group_4689	nucleoid-associated protein	25	core
purD	phosphoribosylamine--glycine ligase	25	core
group_4691	bifunctional chorismate mutase/prephenate dehydratase	25	core
group_4692	thiol oxidoreductase	25	core
group_4693	hypothetical protein	25	core
group_4694	ribosomal-protein-serine acetyltransferase	25	core
mdh	malate dehydrogenase	25	core
group_4696	membrane protein	25	core
group_4697	peptidase M16	25	core
group_4698	hypothetical protein	25	core
panB	3-methyl-2-oxobutanoate hydroxymethyltransferase	25	core
group_47	membrane protein	25	core
rpoB	DNA-directed RNA polymerase subunit beta	25	core
surA	chaperone SurA	25	core
group_4702	lysine decarboxylase CadA	25	core
nrdG	anaerobic ribonucleoside-triphosphate reductase-activating protein	25	core
group_4704	chain-length determining protein	25	core
group_4705	branched-chain amino acid transport system carrier protein	25	core
group_4706	LysR family transcriptional regulator	25	core
group_4707	alanine dehydrogenase	25	core
group_4708	UPF0208 membrane protein	25	core
rlmD	23S rRNA (uracil(1939)-C(5))-methyltransferase RlmD	25	core
serS	serine--tRNA ligase	25	core
lpxC	UDP-3-O-acyl-N-acetylglucosamine deacetylase	25	core
group_4712	translation initiation factor	25	core

group_4713	aminobenzoyl-glutamate transporter	25	core
infB	translation initiation factor IF-2	25	core
opgH	glucans biosynthesis glucosyltransferase H	25	core
group_4716	tRNA-Cys	25	core
group_4717	hypothetical protein	25	core
group_4718	exodeoxyribonuclease III	25	core
group_4719	3-deoxy-7-phosphoheptulonate synthase	25	core
group_4720	electron transport complex subunit B	25	core
group_4721	amino acid transporter	25	core
group_4722	pseudouridine synthase	25	core
group_4723	MarR family transcriptional regulator	25	core
group_4724	cystathionine gamma-synthase	25	core
purT	phosphoribosylglycinamide formyltransferase 2	25	core
group_4726	response regulator	25	core
purL	phosphoribosylformylglycinamidine synthase	25	core
group_4728	SpoVR family protein	25	core
group_4729	inosine/guanosine kinase	25	core
uppP	undecaprenyl-diphosphatase	25	core
epsM	type II secretion system protein M	25	core
group_4732	type VI secretion protein	25	core
group_4733	aconitate hydratase B	25	core
group_4734	TetR family transcriptional regulator	25	core
group_4735	serine transporter	25	core
dapE	succinyl-diaminopimelate desuccinylase	25	core
metK	S-adenosylmethionine synthase	25	core
group_4738	octaprenyl-diphosphate synthase	25	core
clpP	ATP-dependent Clp protease proteolytic subunit	25	core
aroC	chorismate synthase	25	core
group_4741	tyrosine transporter TyrP	25	core
ftsZ	cell division protein FtsZ	25	core
prmB	50S ribosomal protein L3 glutamine methyltransferase	25	core
group_4744	molybdenum cofactor biosynthesis protein B	25	core
group_4745	aminomethyltransferase	25	core
group_4746	sigma-54-dependent Fis family transcriptional regulator	25	core
group_4747	pilus protein PilZ	25	core
plsX	phosphate acyltransferase	25	core
flhB	flagellar biosynthesis protein FlhB	25	core
group_4750	haloacid dehalogenase	25	core
group_4751	thiamin biosynthesis lipoprotein ApbE	25	core

group_4752	carboxylate--amine ligase	25	core
topA	DNA topoisomerase 1	25	core
cmk	cytidylate kinase	25	core
hemE	uroporphyrinogen decarboxylase	25	core
group_4756	universal stress protein	25	core
group_4757	inner membrane transport protein YdhC	25	core
group_4758	organic solvent ABC transporter substrate-binding protein	25	core
mpl	UDP-N-acetylmuramate--L-alanyl-gamma-D-glutamyl-m eso-2,6-diaminoheptandioate ligase	25	core
efp	elongation factor P	25	core
group_4761	phasin family protein	25	core
clcA	H(+)/Cl(-) exchange transporter ClcA	25	core
group_4763	electron transport complex subunit A	25	core
group_4764	acetolactate synthase	25	core
lpd	dihydrolipoyl dehydrogenase	25	core
group_4766	hypothetical protein	25	core
group_4767	Zn-dependent protease	25	core
group_4768	dehydrogenase	25	core
group_4769	citrate (pro-3S)-lyase subunit beta	25	core
thyA	thymidylate synthase	25	core
group_4771	formate transporter FocA	25	core
pepT	peptidase T	25	core
purM	phosphoribosylformylglycinamidine cyclo-ligase	25	core
glmS	glutamine--fructose-6-phosphate aminotransferase [isomerizing]	25	core
gshB	glutathione synthetase	25	core
guaA	GMP synthase [glutamine-hydrolyzing]	25	core
era	GTPase Era	25	core
group_4778	Na+/H+ antiporter NhaC	25	core
group_4779	glutamate synthase	25	core
group_4780	zinc transporter ZntB	25	core
group_4781	1-acyl-sn-glycerol-3-phosphate acyltransferase	25	core
group_4782	hypothetical protein	25	core
group_4783	MFS transporter	25	core
group_4784	(2E,6E)-farnesyl diphosphate synthase	25	core
group_4786	ABC transporter substrate-binding protein	25	core
group_4787	C4-dicarboxylate ABC transporter substrate-binding protein	25	core
cysN	sulfate adenylyltransferase subunit 1	25	core
group_4789	DNA-binding transcriptional regulator OxyR	25	core
group_4790	arginine ABC transporter ATP-binding protein	25	core
group_4791	aspartate kinase	25	core

group_4792	Na+/H+ antiporter	25	core
group_4793	cobyric acid synthase	25	core
group_4794	UDP-N-acetyl glucosamine 2-epimerase	25	core
group_4795	Na+/H+ antiporter NhaD	25	core
group_4796	sodium:proton antiporter	25	core
group_4797	aminotransferase class III	25	core
group_4798	LysR family transcriptional regulator	25	core
nrdB	ribonucleotide-diphosphate reductase subunit beta	25	core
lacZ	beta-galactosidase	25	core
mtnN	5'-methylthioadenosine/S-adenosylhomocysteine nucleosidase	25	core
argS	arginine--tRNA ligase	25	core
group_4802	aspartyl-tRNA amidotransferase subunit B	25	core
group_4803	rod shape-determining protein RodA	25	core
dsdA	putative D-serine dehydratase	25	core
group_4805	NUDIX hydrolase	25	core
ribB_2	3,4-dihydroxy-2-butanone 4-phosphate synthase	25	core
group_4807	phosphoglucomutase, alpha-D-glucose phosphate-specific	25	core
uvrA	UvrABC system protein A	25	core
group_4809	ABC transporter ATP-binding protein	25	core
group_4810	succinate dehydrogenase flavoprotein subunit	25	core
group_4811	response regulator	25	core
trmB	tRNA (guanine-N(7)-)-methyltransferase	25	core
ackA1	acetate kinase 1	25	core
thiI	tRNA sulfurtransferase	25	core
aspS	aspartate--tRNA ligase	25	core
pepB	peptidase B	25	core
bioD	ATP-dependent dethiobiotin synthetase BioD	25	core
group_4818	long-chain fatty acid outer membrane transporter	25	core
group_4819	chromosome partitioning protein ParB	25	core
group_4820	hybrid sensor histidine kinase/response regulator	25	core
potB	spermidine/putrescine ABC transporter permease PotB	25	core
group_4822	ferredoxin	25	core
group_4823	adenosylcobinamide-phosphate synthase	25	core
group_4824	D-ribose ABC transporter substrate-binding protein	25	core
ffh	signal recognition particle protein	25	core
group_4826	carboxynorspermidine synthase	25	core
group_4827	succinate dehydrogenase iron-sulfur subunit	25	core
group_4828	ATP-dependent protease	25	core
nhaA	Na(+)/H(+) antiporter NhaA	25	core

group_4830	iron-sulfur cluster assembly scaffold protein IscU	25	core
pbpG	D-alanyl-D-alanine endopeptidase	25	core
group_4832	histidine/lysine/arginine/ornithine ABC transporter permease HisQ	25	core
nusA	transcription termination/antitermination protein NusA	25	core
pgk	phosphoglycerate kinase	25	core
group_4835	QacE family quaternary ammonium compound efflux SMR transporter	25	core
group_4836	MFS transporter	25	core
group_4837	PTS N-acetylmuramic acid transporter subunit IIBC	25	core
group_4838	NADH oxidase	25	core
rluD	ribosomal large subunit pseudouridine synthase D	25	core
group_4840	transcriptional regulator	25	core
group_4841	diguanylate phosphodiesterase	25	core
group_4842	carbonic anhydrase	25	core
group_4843	methyl-accepting chemotaxis protein	25	core
atpB	ATP synthase subunit a	25	core
group_4845	ATP-dependent endonuclease	25	core
group_4846	NADPH-dependent oxidoreductase	25	core
group_4847	serine endoprotease DegQ	25	core
sdhC	succinate dehydrogenase cytochrome b556 large subunit	25	core
pepA	cytosol aminopeptidase	25	core
group_4851	flagellar biosynthetic protein FliR	25	core
group_4852	4-hydroxyphenylpyruvate dioxygenase	25	core
group_4853	SpoOM-related protein	25	core
group_4854	UPF0246 protein	25	core
group_4855	peptide ABC transporter substrate-binding protein	25	core
group_4856	beta-N-acetylhexosaminidase	25	core
group_4857	ribulose-phosphate 3-epimerase	25	core
group_4858	SAM-dependent methyltransferase	25	core
group_4859	tRNA-Phe	25	core
group_4860	riboflavin synthase subunit alpha	25	core
deoD2	purine nucleoside phosphorylase DeoD-type 2	25	core
group_4862	NAD(P) transhydrogenase subunit alpha	25	core
clpX	ATP-dependent Clp protease ATP-binding subunit ClpX	25	core
group_4864	gonadoliberin III	25	core
dnaK	chaperone protein DnaK	25	core
rnr	ribonuclease R	25	core
group_4867	UPF0178 protein	25	core
group_4868	hypothetical protein	25	core
dctM	C4-dicarboxylate TRAP transporter large permease protein DctM	25	core

group_4870	MATE family efflux transporter	25	core
bamE	outer membrane protein assembly factor BamE	25	core
cysS	cysteine--tRNA ligase	25	core
group_4873	nucleoside permease	25	core
group_4874	hemin ABC transporter permease	25	core
murI	glutamate racemase	25	core
hslU	ATP-dependent protease ATPase subunit HslU	25	core
group_4877	membrane protein	25	core
rsfS	ribosomal silencing factor RsfS	25	core
group_4879	hypothetical protein	25	core
dnaA	chromosomal replication initiator protein DnaA	25	core
rbsA_1	ribose import ATP-binding protein RbsA	25	core
purU	formyltetrahydrofolate deformylase	25	core
group_4883	hypothetical protein	25	core
group_4884	LPS export ABC transporter permease LptF	25	core
lexA	LexA repressor	25	core
group_4886	hypothetical protein	25	core
group_4887	sigma factor RpoE regulatory protein RseC	25	core
group_4888	methyl-accepting chemotaxis protein	25	core
cdd	cytidine deaminase	25	core
group_489	hypothetical protein	25	core
group_4890	sodium:calcium antiporter	25	core
rbsC	ribose ABC transporter permease	25	core
group_4892	CBS domain-containing protein	25	core
group_4893	Bcr/CflA family drug resistance efflux transporter	25	core
bioB	biotin synthase	25	core
cheB1_1	chemotaxis response regulator protein-glutamate methylesterase of group 1 operon	25	core
groL2	60 kDa chaperonin 2	25	core
group_4897	LuxR family transcriptional regulator	25	core
parE	DNA topoisomerase 4 subunit B	25	core
group_4899	flagellar basal-body rod protein FlgF	25	core
tilS	tRNA(Ile)-lysidine synthase	25	core
group_490	peptidoglycan-binding protein	25	core
group_4900	Maf-like protein	25	core
group_4901	nucleoside permease	25	core
rlmN	dual-specificity RNA methyltransferase RlmN	25	core
rplC	50S ribosomal protein L3	25	core
ilvA	L-threonine dehydratase	25	core
group_4905	hypothetical protein	25	core

group_4906	Bcr/CflA family drug resistance efflux transporter	25	core
group_4907	formate transporter FocA	25	core
group_4908	cation:proton antiporter	25	core
spoT	guanosine-3',5'-bis(diphosphate) 3'-pyrophosphohydrolase	25	core
hemF	oxygen-dependent coproporphyrinogen-III oxidase	25	core
yidC	membrane protein insertase YidC	25	core
group_4911	transcriptional initiation protein Tat	25	core
napA	periplasmic nitrate reductase	25	core
group_4913	ABC transporter permease	25	core
group_4914	LysR family transcriptional regulator	25	core
group_4915	phosphate transporter	25	core
group_4916	peptide ABC transporter ATP-binding protein	25	core
group_4917	hypothetical protein	25	core
rlpA	endolytic peptidoglycan transglycosylase RlpA	25	core
group_4919	peptidyl-prolyl cis-trans isomerase	25	core
thiH	thiamine biosynthesis protein ThiH	25	core
fadJ	fatty acid oxidation complex subunit alpha	25	core
fadB	fatty acid oxidation complex subunit alpha	25	core
group_4922	putative transcriptional regulatory protein	25	core
group_4923	putative zinc metalloprotease	25	core
hldE	bifunctional protein HldE	25	core
group_4925	LysR family transcriptional regulator	25	core
group_4926	ATP-dependent protease	25	core
group_4927	DUF368 domain-containing protein	25	core
group_4928	short-chain dehydrogenase	25	core
group_4929	cytochrome bd ubiquinol oxidase subunit II	25	core
group_493	hypothetical protein	25	core
pgi	glucose-6-phosphate isomerase	25	core
group_4931	fimbrial protein	25	core
glpC	sn-glycerol-3-phosphate dehydrogenase subunit C	25	core
prmA	ribosomal protein L11 methyltransferase	25	core
group_4934	hypothetical protein	25	core
rpoH	RNA polymerase sigma factor RpoH	25	core
glmM	phosphoglucosamine mutase	25	core
epsF	type II secretion system protein F	25	core
group_4938	magnesium transporter MgtE	25	core
eno	enolase	25	core
group_494	membrane protein	25	core
group_4940	peptide ABC transporter permease	25	core

group_4941	malate dehydrogenase	25	core
flaB	flagellin B	25	core
group_4943	aminotransferase	25	core
group_4944	ABC transporter substrate-binding protein	25	core
rpoD	RNA polymerase sigma factor RpoD	25	core
sucA	2-oxoglutarate dehydrogenase subunit E1	25	core
nadK	NAD kinase	25	core
rsmB	ribosomal RNA small subunit methyltransferase B	25	core
hemC	porphobilinogen deaminase	25	core
group_495	SCP2 domain-containing protein	25	core
group_4950	hypothetical protein	25	core
group_4951	hypothetical protein	25	core
group_4952	tRNA-modifying protein YgfZ	25	core
asnS	asparagine--tRNA ligase	25	core
dsbA	thiol:disulfide interchange protein DsbA	25	core
rho	transcription termination factor Rho	25	core
group_4956	protease TldD	25	core
group_4957	hypothetical protein	25	core
flaD	flagellin D	25	core
araD	L-ribulose-5-phosphate 4-epimerase	25	core
ubiB	putative protein kinase UbiB	25	core
group_4960	hypothetical protein	25	core
group_4961	phosphoribosylglycinamide formyltransferase	25	core
group_4962	LOG family protein	25	core
group_4963	lipoprotein	25	core
group_4964	acyl-CoA synthetase	25	core
group_4965	T-protein	25	core
leuA	2-isopropylmalate synthase	25	core
group_4967	RNA polymerase sigma-54 factor	25	core
group_4968	sodium-dependent transporter	25	core
group_4969	ADP-ribose pyrophosphatase	25	core
ubiA	4-hydroxybenzoate octaprenyltransferase	25	core
flgA	flagella basal body P-ring formation protein FlgA	25	core
group_4971	DNA-binding transcriptional regulator FruR	25	core
vdcA	diguanylate cyclase VdcA	25	core
hemL	glutamate-1-semialdehyde 2,1-aminomutase	25	core
group_4974	hypothetical protein	25	core
group_4975	UPF0234 protein	25	core
group_4976	UPF0260 protein	25	core

group_4977	MFS transporter	25	core
group_4978	tRNA-Arg	25	core
recG	ATP-dependent DNA helicase RecG	25	core
group_498	TatD family hydrolase	25	core
group_4980	N,N'-diacetylchitobiose phosphorylase	25	core
purN	phosphoribosylglycinamide formyltransferase	25	core
group_4982	adenylosuccinate lyase	25	core
rimP	ribosome maturation factor RimP	25	core
group_4984	DNA polymerase III subunit epsilon	25	core
group_4985	hemolysin	25	core
iscS	cysteine desulfurase IscS	25	core
group_4987	phage shock protein C	25	core
group_4988	superoxide dismutase	25	core
group_4989	nucleoside triphosphate hydrolase	25	core
group_499	cytochrome c	25	core
argH	argininosuccinate lyase	25	core
argG	argininosuccinate synthase	25	core
kdsA	2-dehydro-3-deoxyphosphooctonate aldolase	25	core
group_4993	DEAD/DEAH box helicase	25	core
folE	GTP cyclohydrolase 1	25	core
group_4995	membrane protein	25	core
fabH1	3-oxoacyl-[acyl-carrier-protein] synthase 3 protein 1	25	core
group_4997	sodium:phosphate symporter	25	core
group_4998	alpha-L-glutamate ligase-like protein	25	core
fxsA	membrane protein FxsA	25	core
group_5	hypothetical protein	25	core
group_50	membrane protein	25	core
group_500	coproporphyrinogen-III oxidase	25	core
group_5000	diguanylate cyclase	25	core
uvrC	UvrABC system protein C	25	core
rsmI	ribosomal RNA small subunit methyltransferase I	25	core
group_5003	DUF805 domain-containing protein	25	core
group_5004	DNA helicase	25	core
group_5005	isocitrate lyase	25	core
group_5006	phosphatidate cytidylyltransferase	25	core
ispH	4-hydroxy-3-methylbut-2-enyl diphosphate reductase	25	core
secD_1	protein translocase subunit SecD	25	core
msbA	lipid A export ATP-binding/permease protein MsbA	25	core
flgK	flagellar hook protein FlgK	25	core

ansA	L-asparaginase 1	25	core
group_5012	peptide ABC transporter permease	25	core
cdpA	cyclic di-GMP phosphodiesterase CdpA	25	core
group_5014	protein lysine acetyltransferase	25	core
group_5015	phospho-2-dehydro-3-deoxyheptonate aldolase	25	core
rseB	sigma-E factor regulatory protein RseB	25	core
mnmE	tRNA modification GTPase MnmE	25	core
group_5018	flavodoxin FldB	25	core
group_5019	hypothetical protein	25	core
cyaY	protein CyaY	25	core
group_5020	ABC transporter permease	25	core
alaE	L-alanine exporter AlaE	25	core
group_5022	hypothetical protein	25	core
group_5023	hypothetical protein	25	core
group_5024	UPF0761 membrane protein	25	core
deaD	ATP-dependent RNA helicase DeaD	25	core
cheD	putative chemoreceptor glutamine deamidase CheD	25	core
group_5027	sensor histidine kinase	25	core
rnt	ribonuclease T	25	core
pcnB	poly(A) polymerase I	25	core
lysA	diaminopimelate decarboxylase	25	core
metF	5,10-methylenetetrahydrofolate reductase	25	core
group_5032	cell division protein DedD	25	core
group_5033	TetR family transcriptional regulator	25	core
queG	epoxyqueuosine reductase	25	core
group_5035	alcohol dehydrogenase	25	core
group_5036	cytochrome b	25	core
group_5037	paraslipin	25	core
flaE	flagellin E	25	core
group_5039	basal-body rod modification protein FlgD	25	core
dapF	diaminopimelate epimerase	25	core
group_5040	hypothetical protein	25	core
group_5041	LysR family transcriptional regulator	25	core
group_5042	cyclic di-GMP binding protein	25	core
group_5043	ABC transporter substrate-binding protein	25	core
group_5044	hypothetical protein	25	core
group_5045	transcriptional regulator IlvY	25	core
group_5046	pyruvate dehydrogenase E1 component	25	core
group_5047	ferritin	25	core

fabF	3-oxoacyl-[acyl-carrier-protein] synthase 2	25	core
group_5049	transporter	25	core
group_505	membrane protein	25	core
group_5050	hypothetical protein	25	core
hisA	1-(5-phosphoribosyl)-5-[(5-phosphoribosylamino)me thylideneamino] imidazole-4-carboxamide isomerase	25	core
group_5052	outer membrane-stress sensor serine endopeptidase DegS	25	core
group_5053	ABC transporter substrate-binding protein	25	core
group_5054	ribosome biogenesis GTPase A	25	core
priA	primosomal protein N'	25	core
hisF	imidazole glycerol phosphate synthase subunit HisF	25	core
pnp	polyribonucleotide nucleotidyltransferase	25	core
ectB	diaminobutyrate--2-oxoglutarate transaminase	25	core
rraA	regulator of ribonuclease activity A	25	core
group_506	ribosomal RNA small subunit methyltransferase D	25	core
proS	proline--tRNA ligase	25	core
mglA	galactose/methyl galactoside import ATP-binding protein MglA	25	core
group_5062	cobyric acid synthase CobQ	25	core
group_5063	threonylcarbamoyl-AMP synthase	25	core
group_5064	hypothetical protein	25	core
group_5065	uracil permease	25	core
group_5066	anaerobic C4-dicarboxylate transporter	25	core
group_5067	MGMT family protein	25	core
group_5068	putrescine-binding periplasmic protein	25	core
group_5069	agmatinase	25	core
group_5070	L-ascorbate-6-phosphate lactonase	25	core
speA	biosynthetic arginine decarboxylase	25	core
group_5072	acetolactate synthase	25	core
fieF	cation-efflux pump FieF	25	core
group_5074	6-carboxy-5,6,7,8-tetrahydropterin synthase	25	core
group_5075	NADPH-dependent 2,4-dienoyl-CoA reductase	25	core
secY	protein translocase subunit SecY	25	core
rmuC	DNA recombination protein RmuC	25	core
pheT	phenylalanine--tRNA ligase beta subunit	25	core
pckA	phosphoenolpyruvate carboxykinase [ATP]	25	core
group_5080	GTP pyrophosphokinase	25	core
recR	recombination protein RecR	25	core
group_5082	membrane protein	25	core
gppA	guanosine-5'-triphosphate,3'-diphosphate pyrophosphatase	25	core
group_5084	GGDEF domain-containing protein	25	core

lgt	prolipoprotein diacylglyceryl transferase	25	core
group_5086	peroxidase	25	core
group_5087	methyl-accepting chemotaxis protein	25	core
group_5088	cysteine synthase	25	core
group_5089	aromatic amino acid exporter	25	core
group_509	RNA-binding protein	25	core
group_5090	chemotaxis protein CheC	25	core
group_5091	transcriptional regulatory protein	25	core
group_5092	AraC family transcriptional regulator	25	core
lipB	octanoyltransferase	25	core
group_5094	c-type cytochrome biogenesis protein CcmF	25	core
dnaN	DNA polymerase III subunit beta	25	core
group_5096	aldehyde dehydrogenase	25	core
group_5098	hypothetical protein	25	core
group_5099	trimethylamine N-oxide reductase I catalytic subunit	25	core
group_5100	YciK family oxidoreductase	25	core
purR	HTH-type transcriptional repressor PurR	25	core
group_5102	ribonuclease G	25	core
helD	DNA helicase IV	25	core
group_5104	proton/glutamate symporter	25	core
group_5105	AMP-dependent synthetase	25	core
group_5106	methyl-accepting chemotaxis protein	25	core
panC	pantothenate synthetase	25	core
group_5108	bifunctional folylpolyglutamate synthase/dihydrofolate synthase	25	core
group_5109	MaoC family dehydratase	25	core
group_5110	hypothetical protein	25	core
group_5111	flagellar M-ring protein	25	core
group_5112	ABC transporter ATP-binding protein	25	core
group_5113	sodium:proton antiporter	25	core
secA	protein translocase subunit SecA	25	core
group_5115	flagellar motor switch protein FliM	25	core
group_5116	ABC transporter ATP-binding protein	25	core
group_5117	tRNA threonylcarbamoyladenosine dehydratase	25	core
srmB	ATP-dependent RNA helicase SrmB	25	core
group_5119	hypothetical protein	25	core
hslO	33 kDa chaperonin	25	core
cmoM	tRNA 5-carboxymethoxyuridine methyltransferase	25	core
group_5121	phosphoenolpyruvate-protein phosphotransferase	25	core
aroA	3-phosphoshikimate 1-carboxyvinyltransferase	25	core

group_5123	universal stress protein E	25	core
group_5124	exonuclease	25	core
group_5125	MFS transporter	25	core
group_5126	universal stress protein	25	core
glpA	sn-glycerol-3-phosphate dehydrogenase subunit A	25	core
group_5128	putative transport protein	25	core
bamC	outer membrane protein assembly factor BamC	25	core
epsH	type II secretion system protein H	25	core
group_5130	chemotaxis protein CheW	25	core
tmk	thymidylate kinase	25	core
group_5132	DNA transformation protein	25	core
group_5133	mycothiol reductase	25	core
dxs	1-deoxy-D-xylulose-5-phosphate synthase	25	core
group_5135	thermolabile hemolysin	25	core
thrA	bifunctional aspartokinase I/homoserine dehydrogenase I	25	core
group_5137	sigma-54-dependent Fis family transcriptional regulator	25	core
group_5138	inositol-1-monophosphatase	25	core
gmhA_2	phosphoheptose isomerase	25	core
epsJ	type II secretion system protein J	25	core
rep	ATP-dependent DNA helicase Rep	25	core
group_5141	fumarate hydratase class I	25	core
matP	macrodomain Ter protein	25	core
atpD	ATP synthase subunit beta	25	core
group_5144	3'3'-cGAMP-specific phosphodiesterase 2	25	core
group_5145	C4-dicarboxylate ABC transporter substrate-binding protein	25	core
group_5146	transcriptional regulator	25	core
exbD1	biopolymer transport protein exbD1	25	core
ribA	GTP cyclohydrolase-2	25	core
group_5149	phosphoesterase	25	core
group_5150	ATP-dependent Zn protease	25	core
group_5151	hypothetical protein	25	core
fliI	flagellum-specific ATP synthase	25	core
rplK	50S ribosomal protein L11	25	core
nadA	quinolinate synthase A	25	core
group_5155	PTS sucrose transporter subunit IIBC	25	core
group_5156	DNA-binding response regulator	25	core
group_5157	CinA-like protein	25	core
group_5158	molecular chaperone DnaJ	25	core
group_5159	ferredoxin	25	core

group_5160	hypothetical protein	25	core
group_5161	polymerase	25	core
gpmI	2,3-bisphosphoglycerate-independent phosphoglycerate mutase	25	core
group_5163	amino acid ABC transporter substrate-binding protein	25	core
group_5164	LysR family transcriptional regulator	25	core
group_5165	NAD-glutamate dehydrogenase	25	core
group_5166	two-component system response regulator	25	core
atpC	ATP synthase epsilon chain	25	core
group_5168	UPF0115 protein	25	core
rbsK	ribokinase	25	core
group_517	DNA-binding response regulator	25	core
group_5170	UPF0125 protein	25	core
group_5171	antimicrobial peptide ABC transporter permease SapB	25	core
group_5172	L-cystine transporter tcyP	25	core
group_5173	DeoR family transcriptional regulator	25	core
lepA	elongation factor 4	25	core
group_5175	FixG-like protein	25	core
group_5176	amino acid ABC transporter permease	25	core
group_5177	ABC transporter ATP-binding protein	25	core
group_5178	phosphatase	25	core
group_5179	ABC transporter permease	25	core
frdC	fumarate reductase subunit C	25	core
secB	protein-export protein SecB	25	core
pheS	phenylalanine--tRNA ligase alpha subunit	25	core
group_5182	membrane protein	25	core
group_5183	DNA starvation/stationary phase protection protein	25	core
group_5184	UPF0267 protein	25	core
gcvH	glycine cleavage system H protein	25	core
group_5186	transcriptional regulator GcvA	25	core
deoB	phosphopentomutase	25	core
secF_2	protein-export membrane protein SecF	25	core
group_5189	LysR family transcriptional regulator	25	core
group_519	hypothetical protein	25	core
group_5190	transcriptional regulator	25	core
group_5191	membrane-bound lytic murein transglycosylase C	25	core
group_5192	hypothetical protein	25	core
group_5193	NAD(P) transhydrogenase subunit beta	25	core
tgt	queuine tRNA-ribosyltransferase	25	core
group_5195	GGDEF domain-containing protein	25	core

uppS	ditrans,polycis-undecaprenyl-diphosphate synthase ((2E,6E)-farnesyl-diphosphate specific)	25	core
group_5197	hypothetical protein	25	core
group_5198	protein YebE	25	core
group_5199	type VI secretion protein IcmF	25	core
group_52	hypothetical protein	25	core
mrcA	penicillin-binding protein 1A	25	core
group_5200	peptidase M20	25	core
group_5201	dihydrolipoyllysine-residue succinyltransferase component of 2-oxoglutarate dehydrogenase complex	25	core
group_5202	AI-2E family transporter	25	core
cheR3	chemotaxis protein methyltransferase 3	25	core
erpA	iron-sulfur cluster insertion protein ErpA	25	core
group_5205	Trk system potassium uptake protein	25	core
group_5206	helix-turn-helix transcriptional regulator	25	core
bamD	outer membrane protein assembly factor BamD	25	core
group_5208	sodium/glutamate symporter	25	core
group_5209	MFS transporter	25	core
group_521	nuclease	25	core
ppc	phosphoenolpyruvate carboxylase	25	core
group_5211	30S ribosomal protein S1	25	core
tal	transaldolase	25	core
trmD	tRNA (guanine-N(1)-)-methyltransferase	25	core
group_5214	DUF1338 domain-containing protein	25	core
glgB	1,4-alpha-glucan branching enzyme GlgB	25	core
queE	7-carboxy-7-deazaguanine synthase	25	core
group_5217	penicillin-binding protein 3	25	core
katG	catalase-peroxidase	25	core
apt	adenine phosphoribosyltransferase	25	core
group_522	glutamine amidotransferase	25	core
group_5220	hypothetical protein	25	core
ftsW	putative lipid II flippase FtsW	25	core
glyA1	serine hydroxymethyltransferase 1	25	core
group_5223	AsnC family transcriptional regulator	25	core
group_5224	hypothetical protein	25	core
group_5225	DNA-binding response regulator	25	core
group_5226	site-determining protein	25	core
emrD	Bcr/CflA family drug resistance efflux transporter	25	core
group_5228	sodium-independent anion transporter	25	core
group_5229	UPF0251 protein	25	core
group_523	metal-binding protein	25	core

group_5230	membrane protein	25	core
map	methionine aminopeptidase	25	core
group_5232	putative beta-barrel assembly-enhancing protease	25	core
group_5233	fumarate reductase flavoprotein subunit	25	core
group_5234	PTS glucose transporter subunit IIBC	25	core
group_5235	phosphoglycerate transport regulatory protein PgtC	25	core
group_5236	UPF0162 protein	25	core
group_5237	alpha-1,4 glucan phosphorylase	25	core
group_5238	hypothetical protein	25	core
trpS	tryptophan--tRNA ligase	25	core
group_524	hydrolase	25	core
group_5240	RNA-binding transcriptional accessory protein	25	core
ppx	exopolyphosphatase	25	core
group_5242	hydrolase	25	core
xerD	tyrosine recombinase XerD	25	core
group_5244	iron(III) ABC transporter ATP-binding protein	25	core
group_5245	nitrogen regulation protein NR(I)	25	core
cysD	sulfate adenylyltransferase subunit 2	25	core
group_5247	threonine transporter RhtB	25	core
murF	UDP-N-acetylmuramoyl-tripeptide--D-alanyl-D-alani ne ligase	25	core
group_5249	lytic transglycosylase	25	core
rplF	50S ribosomal protein L6	25	core
group_5250	heat shock protein 15	25	core
greA	transcription elongation factor GreA	25	core
group_5252	hypothetical protein	25	core
glyQ	glycine--tRNA ligase alpha subunit	25	core
metL	bifunctional aspartate kinase/homoserine dehydrogenase II	25	core
trmL	tRNA (cytidine(34)-2'-O)-methyltransferase	25	core
rpiA	ribose-5-phosphate isomerase A	25	core
rsmG	ribosomal RNA small subunit methyltransferase G	25	core
group_5258	ABC transporter ATP-binding protein	25	core
group_5259	formate acetyltransferase	25	core
rpoA	DNA-directed RNA polymerase subunit alpha	25	core
group_5260	isopentenyl-diphosphate Delta-isomerase	25	core
group_5261	ClpV1 family T6SS ATPase	25	core
speG	spermidine N(1)-acetyltransferase	25	core
sstT	serine/threonine transporter SstT	25	core
group_5264	glutaredoxin	25	core
mltF	membrane-bound lytic murein transglycosylase F	25	core

group_5266	sigma-54-dependent Fis family transcriptional regulator	25	core
group_5267	LacI family transcriptional regulator	25	core
ubiD	3-octaprenyl-4-hydroxybenzoate carboxy-lyase	25	core
group_5269	heat-shock protein HslJ	25	core
group_527	ElaA protein	25	core
group_5270	tRNA-Cys	25	core
group_5271	long-chain-fatty-acid--CoA ligase	25	core
norM	multidrug resistance protein NorM	25	core
group_5273	UPF0229 protein	25	core
smpB	SsrA-binding protein	25	core
cgtA	GTPase Obg/CgtA	25	core
group_5276	DNA-binding response regulator	25	core
maeA	NAD-dependent malic enzyme	25	core
group_5278	GlcNAc phosphomutase	25	core
group_5279	hypothetical protein	25	core
group_528	ATP-dependent RNA helicase	25	core
group_5280	3-phenylpropionic acid transporter	25	core
trpB	tryptophan synthase beta chain	25	core
nfo	putative endonuclease 4	25	core
group_5283	multidrug resistance protein	25	core
group_5284	molecular chaperone TorD	25	core
minC	putative septum site-determining protein MinC	25	core
murC	UDP-N-acetylmuramate--L-alanine ligase	25	core
group_5287	MerR family transcriptional regulator	25	core
group_5288	ABC transporter ATPase	25	core
group_5289	outer membrane protein assembly factor	25	core
group_529	uroporphyrin-III C-methyltransferase	25	core
pdxH	pyridoxine/pyridoxamine 5'-phosphate oxidase	25	core
group_5291	C4-dicarboxylate ABC transporter	25	core
group_5292	ribosomal protein S6 modification protein	25	core
group_5293	cell division protein FtsX	25	core
nqrA	Na(+)-translocating NADH-quinone reductase subunit A	25	core
group_5295	histidine kinase	25	core
group_5296	hypothetical protein	25	core
group_5297	cell division protein DedD	25	core
group_5298	glutathione amide-dependent peroxidase	25	core
group_5299	penicillin-binding protein 2	25	core
menC	o-succinylbenzoate synthase	25	core
group_530	hypothetical protein	25	core

group_5300	multidrug resistance protein	25	core
group_5301	DUF4145 domain-containing protein	25	core
group_5302	peptidyl-prolyl cis-trans isomerase	25	core
fbp	fructose-1,6-bisphosphatase class 1	25	core
group_5304	3'-5' exonuclease	25	core
ybeY	endoribonuclease YbeY	25	core
group_5306	ligand-gated channel protein	25	core
group_5307	hemolysin secretion protein D	25	core
group_5308	cysteine desulfurase-like protein	25	core
group_5309	cytochrome C biogenesis protein CcmH	25	core
group_531	DNA polymerase III subunit chi	25	core
djlA	co-chaperone protein DjlA	25	core
group_5311	glycerol-3-phosphate acyltransferase	25	core
rlmL	ribosomal RNA large subunit methyltransferase K/L	25	core
group_5313	beta-ketoacyl-[acyl-carrier-protein] synthase I	25	core
group_5314	agglutination protein	25	core
group_5315	phosphoribulokinase	25	core
group_5316	ATPase AAA	25	core
group_5317	methyl-accepting chemotaxis protein	25	core
group_5318	C-terminal processing peptidase	25	core
apaG	protein ApaG	25	core
group_532	HD family phosphohydrolase	25	core
tdh	L-threonine 3-dehydrogenase	25	core
argP	HTH-type transcriptional regulator ArgP	25	core
group_5322	anaerobic ribonucleoside triphosphate reductase	25	core
group_5323	LysR family transcriptional regulator	25	core
group_5324	CDP-diacylglycerol--serine O-phosphatidyltransferase	25	core
group_5325	rod shape-determining protein MreD	25	core
group_5326	dihydrolipoyl dehydrogenase	25	core
mnmA	tRNA-specific 2-thiouridylase MnmA	25	core
ruvB	Holliday junction ATP-dependent DNA helicase RuvB	25	core
group_5329	ligand-gated channel	25	core
group_5330	peptidyl-prolyl cis-trans isomerase	25	core
group_5331	cytochrome c	25	core
rnb	exoribonuclease 2	25	core
group_5333	UPF0313 protein	25	core
dinG	ATP-dependent DNA helicase DinG	25	core
ompV	outer membrane protein OmpV	25	core
hscA	chaperone protein HscA	25	core

group_5337	MBL fold metallo-hydrolase	25	core
group_5338	glycerol-3-phosphate transporter permease	25	core
lplA	lipoate-protein ligase A	25	core
group_5340	hypothetical protein	25	core
dsbD	thiol:disulfide interchange protein DsbD	25	core
gltD_2	glutamate synthase subunit beta	25	core
luxP	autoinducer 2-binding periplasmic protein LuxP	25	core
group_5345	hypothetical protein	25	core
gspK	glucosamine kinase GspK	25	core
group_5347	PTS fructose transporter subunit IIBC	25	core
group_5348	pilus assembly protein PilN	25	core
group_5349	iron-enterobactin transporter ATP-binding protein	25	core
group_535	2-octaprenyl-6-methoxyphenol hydroxylase	25	core
group_5350	transcriptional regulator	25	core
group_5351	phosphoethanolamine transferase	25	core
group_5352	iron-regulated protein A	25	core
fabV2	enoyl-[acyl-carrier-protein] reductase [NADH] 2	25	core
group_5354	hypothetical protein	25	core
group_5355	EF-P beta-lysylation protein EpmB	25	core
viuB	vibriobactin utilization protein ViuB	25	core
group_5357	exodeoxyribonuclease I	25	core
group_5358	2-succinyl-6-hydroxy-2,4-cyclohexadiene-1-carboxy late synthase	25	core
group_5359	multidrug resistance protein	25	core
group_536	anti-sigma-E factor RseA	25	core
dadA	D-amino acid dehydrogenase	25	core
group_5361	menaquinone-specific isochorismate synthase	25	core
group_5362	dihydropteroate synthase	25	core
hisC	histidinol-phosphate aminotransferase	25	core
group_5364	AraC family transcriptional regulator	25	core
group_5365	membrane protein	25	core
ectC	L-ectoine synthase	25	core
group_5367	cobalt-zinc-cadmium resistance protein	25	core
epsG	type II secretion system protein G	25	core
murE	UDP-N-acetylmuramoyl-L-alanyl-D-glutamate--2,6-di aminopimelate ligase	25	core
acpS	holo-[acyl-carrier-protein] synthase	25	core
toxS	transmembrane regulatory protein ToxS	25	core
group_5371	chromosome segregation ATPase	25	core
group_5372	two-component sensor histidine kinase	25	core
group_5373	hypothetical protein	25	core

group_5374	phosphonate utilization transcriptional regulator PhnR	25	core
padC	putative phenolic acid decarboxylase	25	core
group_5376	oxidoreductase	25	core
mscL	large-conductance mechanosensitive channel	25	core
group_5378	two-component sensor histidine kinase	25	core
group_5379	GGDEF domain-containing protein	25	core
group_538	diguanylate cyclase	25	core
group_5380	methyl-accepting chemotaxis protein	25	core
group_5381	mechanosensitive ion channel protein MscS	25	core
group_5382	DUF3450 domain-containing protein	25	core
dsbE	thiol:disulfide interchange protein DsbE	25	core
pncB	nicotinate phosphoribosyltransferase	25	core
group_5385	lysozyme	25	core
group_5386	LysR family transcriptional regulator	25	core
group_5387	regulatory protein LuxO	25	core
group_5388	1,4-alpha-glucan-branching protein	25	core
group_5389	LysR family transcriptional regulator	25	core
mazG	nucleoside triphosphate pyrophosphohydrolase	25	core
group_5390	NAD(P)H nitroreductase	25	core
group_5391	membrane protein	25	core
group_5392	membrane protein	25	core
malS	alpha-amylase	25	core
group_5394	hypothetical protein	25	core
group_5395	2-octaprenyl-6-methoxyphenyl hydroxylase	25	core
group_5396	iron-hydroxamate ABC transporter substrate-binding protein	25	core
group_5397	DNA transformation protein	25	core
group_5398	lysine transporter LysE	25	core
group_5399	sensor protein TorS	25	core
group_54	hypothetical protein	25	core
cpdA	3',5'-cyclic adenosine monophosphate phosphodiesterase CpdA	25	core
group_5400	DNA processing protein DprA	25	core
lolD	lipoprotein-releasing system ATP-binding protein LolD	25	core
group_5402	phosphate ABC transporter permease	25	core
rnd	ribonuclease D	25	core
group_5404	rhomboid family intramembrane serine protease GlpG	25	core
dnaE	DNA polymerase III subunit alpha	25	core
group_5406	kinase inhibitor	25	core
group_5407	TetR family transcriptional regulator	25	core
group_5408	Trk system potassium uptake protein	25	core

group_5409	2-succinyl-6-hydroxy-2,4-cyclohexadiene-1-carboxy late synthase	25	core
group_541	esterase YqiA	25	core
group_5410	U32 family peptidase	25	core
ftsQ	cell division protein FtsQ	25	core
group_5412	hypothetical protein	25	core
group_5413	hypothetical protein	25	core
group_5414	hypothetical protein	25	core
purE	N5-carboxyaminoimidazole ribonucleotide mutase	25	core
slmA	nucleoid occlusion factor SlmA	25	core
group_5417	spindolin	25	core
irgB	iron-regulated virulence regulatory protein IrgB	25	core
group_5419	DNA polymerase I	25	core
group_542	type 4 prepilin-like proteins leader peptide-processing enzyme	25	core
ihfA	integration host factor subunit alpha	25	core
coaBC	coenzyme A biosynthesis bifunctional protein CoaBC	25	core
group_5422	glycerophosphoryl diester phosphodiesterase	25	core
group_5423	LysR family transcriptional regulator	25	core
glyA2	serine hydroxymethyltransferase 2	25	core
rsmC	ribosomal RNA small subunit methyltransferase C	25	core
group_5426	GGDEF domain-containing protein	25	core
group_5427	transcriptional regulator	25	core
leuC	3-isopropylmalate dehydratase large subunit	25	core
rlmH	ribosomal RNA large subunit methyltransferase H	25	core
group_543	N-acetyl-anhydromuranmyl-L-alanine amidase	25	core
group_5430	aldo/keto reductase	25	core
vibB	vibriobactin-specific isochorismatase	25	core
rdgC	recombination-associated protein RdgC	25	core
group_5433	cell division protein FtsN	25	core
group_5434	phosphate transport system regulatory protein PhoU	25	core
hmuV	hemin importer ATP-binding protein	25	core
rsmJ	ribosomal RNA small subunit methyltransferase J	25	core
group_5437	hypothetical protein	25	core
hisB	histidine biosynthesis bifunctional protein HisB	25	core
group_5439	sodium/solute symporter	25	core
group_544	ssDNA exonuclease RecJ	25	core
group_5440	hypothetical protein	25	core
group_5441	organic hydroperoxide resistance protein	25	core
group_5442	NADH-dependent alcohol dehydrogenase	25	core
group_5443	TVP38/TMEM64 family protein	25	core

group_5444	multidrug ABC transporter permease	25	core
greB	transcription elongation factor GreB	25	core
group_5446	hypothetical protein	25	core
group_5447	membrane protein	25	core
group_5448	hypothetical protein	25	core
group_5449	hypothetical protein	25	core
ruvC	crossover junction endodeoxyribonuclease RuvC	25	core
group_5451	membrane protein	25	core
group_5452	ferrous iron transport protein B	25	core
group_5453	DNA mismatch repair protein MutT	25	core
recO	DNA repair protein RecO	25	core
group_5455	type VI secretion-associated protein	25	core
hisH	imidazole glycerol phosphate synthase subunit HisH	25	core
group_5457	ABC transporter substrate-binding protein	25	core
group_5458	NADH dehydrogenase	25	core
group_5459	sodium-dependent transporter	25	core
carA	carbamoyl-phosphate synthase small chain	25	core
group_5460	lipid A biosynthesis acyltransferase	25	core
fliH	flagellar assembly protein FliH	25	core
group_5462	cell division protein DamX	25	core
gloA	putative lactoylglutathione lyase	25	core
zapC	cell division protein ZapC	25	core
group_5465	aminopeptidase	25	core
group_5466	nucleotide pyrophosphatase	25	core
avtA	valine--pyruvate transaminase	25	core
argE	acetylornithine deacetylase	25	core
group_5469	DNA polymerase	25	core
group_547	UPF0721 transmembrane protein	25	core
group_5470	hydrolase	25	core
dsbB	disulfide bond formation protein B	25	core
group_5472	AsnC family transcriptional regulator	25	core
group_5473	hypothetical protein	25	core
ubiX	flavin prenyltransferase UbiX	25	core
zapD	cell division protein ZapD	25	core
group_5476	hypothetical protein	25	core
group_5477	putative gluconeogenesis factor	25	core
treR	trehalose operon repressor	25	core
group_5479	glyceraldehyde-3-phosphate dehydrogenase	25	core
group_548	hypothetical protein	25	core

group_5480	RTX toxin transporter	25	core
group_5481	alkene reductase	25	core
group_5482	hypothetical protein	25	core
group_5483	hypothetical protein	25	core
adk	adenylate kinase	25	core
group_5485	hypothetical protein	25	core
group_5486	methyl-accepting chemotaxis protein	25	core
lolA	outer-membrane lipoprotein carrier protein	25	core
group_5488	hemolysin	25	core
group_5489	cytochrome b	25	core
group_549	threonine synthase	25	core
group_5490	methyl-accepting chemotaxis protein	25	core
group_5491	hypothetical protein	25	core
group_5492	sulfurtransferase TusB	25	core
luxQ	autoinducer 2 sensor kinase/phosphatase LuxQ	25	core
group_5494	thiol:disulfide interchange protein DsbC	25	core
rppH	RNA pyrophosphohydrolase	25	core
glyS	glycine--tRNA ligase beta subunit	25	core
group_5497	CDP-diacylglycerol--glycerol-3-phosphate 3-phosphatidyltransferase-like protein	25	core
group_5498	LysR family transcriptional regulator	25	core
deoC	deoxyribose-phosphate aldolase	25	core
group_55	magnesium transporter MgtE	25	core
group_550	sodium:alanine symporter	25	core
group_5500	(Fe-S)-binding protein	25	core
group_5501	hypothetical protein	25	core
group_5502	carboxypeptidase M32	25	core
radA	DNA repair protein RadA	25	core
group_5504	deoxyguanosinetriphosphate triphosphohydrolase-like protein 1	25	core
moaA	GTP 3',8-cyclase	25	core
group_5506	gamma-glutamyltranspeptidase	25	core
group_5507	chitinase	25	core
epsK	type II secretion system protein K	25	core
group_5509	periplasmic protease	25	core
deoA	thymidine phosphorylase	25	core
group_5510	lipoprotein	25	core
group_5511	FAD-dependent oxidoreductase	25	core
group_5512	anaerobic C4-dicarboxylate transporter	25	core
group_5513	citrate synthase	25	core
kefG	glutathione-regulated potassium-efflux system ancillary protein KefG	25	core

coaD	phosphopantetheine adenylyltransferase	25	core
group_5516	glycine cleavage system transcriptional regulator	25	core
group_5517	NAD-dependent succinate-semialdehyde dehydrogenase	25	core
group_5518	phosphoribosyl-AMP cyclohydrolase	25	core
group_5519	acetyltransferase component of pyruvate dehydrogenase complex	25	core
group_552	pilus protein PilZ	25	core
group_5520	hypothetical protein	25	core
group_5521	MexH family multidrug efflux RND transporter periplasmic adaptor subunit	25	core
sthA	soluble pyridine nucleotide transhydrogenase	25	core
ribN	riboflavin transporter	25	core
group_5524	transporter	25	core
group_5525	glucosaminidase	25	core
group_5526	transcriptional repressor	25	core
group_5527	hypothetical protein	25	core
group_5528	glycosyl transferase family 51	25	core
rhtB	homoserine/homoserine lactone efflux protein	25	core
fusA2	elongation factor G 2	25	core
group_5530	ABC transporter substrate-binding protein	25	core
ccmE	cytochrome c-type biogenesis protein CcmE	25	core
group_5532	major outer membrane lipoprotein	25	core
group_5533	anti-anti-sigma regulatory factor	25	core
asd2	aspartate-semialdehyde dehydrogenase 2	25	core
group_5535	sensor domain-containing phosphodiesterase	25	core
group_5536	hypothetical protein	25	core
group_5537	hypothetical protein	25	core
group_5538	hypothetical protein	25	core
group_5539	ATP-dependent protease	25	core
group_554	chorismate-binding protein	25	core
group_5540	membrane protein	25	core
tusA	sulfurtransferase TusA	25	core
group_5542	5'-deoxynucleotidase	25	core
hmp	flavohemoprotein	25	core
group_5544	hypothetical protein	25	core
group_5545	hypothetical protein	25	core
proA	gamma-glutamyl phosphate reductase	25	core
group_5547	sigma D regulator	25	core
ulaD	3-keto-L-gulonate-6-phosphate decarboxylase	25	core
group_5549	hypothetical protein	25	core
group_555	tRNA-binding protein	25	core

nfuA	Fe/S biogenesis protein NfuA	25	core
malT	HTH-type transcriptional regulator MalT	25	core
group_5552	6-phospho-beta-glucosidase	25	core
lpxD	UDP-3-O-acylglucosamine N-acyltransferase	25	core
group_5554	hypothetical protein	25	core
group_5555	MSHA biogenesis protein MshM	25	core
group_5556	GGDEF domain-containing protein	25	core
group_5557	lysophospholipase L2	25	core
xerC	tyrosine recombinase XerC	25	core
group_5559	DUF4442 domain-containing protein	25	core
group_556	2-hydroxyacid dehydrogenase	25	core
gluQ	glutamyl-Q tRNA(Asp) synthetase	25	core
group_5561	D-hexose-6-phosphate mutarotase	25	core
surE	5'-nucleotidase SurE	25	core
add	adenosine deaminase	25	core
group_5564	ABC transporter permease	25	core
wecC	UDP-N-acetyl-D-mannosamine dehydrogenase	25	core
group_5566	copper amine oxidase	25	core
thiG	thiazole synthase	25	core
clsA	cardiolipin synthase A	25	core
group_5569	flagellar motor protein MotA	25	core
recC	RecBCD enzyme subunit RecC	25	core
group_5570	phosphate transport system permease protein PstA	25	core
group_5571	TetR family transcriptional regulator	25	core
group_5572	ATP-dependent helicase	25	core
group_5573	peptide ABC transporter substrate-binding protein	25	core
bamB	outer membrane protein assembly factor BamB	25	core
ilvM	acetolactate synthase	25	core
flaG	protein FlaG	25	core
group_5577	hydrolase	25	core
ligA	DNA ligase	25	core
hscB	co-chaperone protein HscB	25	core
argA	amino-acid acetyltransferase	25	core
group_5580	hydrolase	25	core
group_5581	2-amino-4-hydroxy-6- hydroxymethyldihydropteridine diphosphokinase	25	core
aroE	shikimate dehydrogenase (NADP(+))	25	core
trmJ	tRNA (cytidine/uridine-2'-O-)-methyltransferase TrmJ	25	core
group_5584	sodium-type flagellar protein MotY	25	core
rsmH	ribosomal RNA small subunit methyltransferase H	25	core

artM	arginine transporter permease subunit ArtM	25	core
group_5587	BolA family transcriptional regulator	25	core
glpK	glycerol kinase	25	core
group_5589	co-chaperone YbbN	25	core
group_559	deacylase	25	core
group_5590	hypothetical protein	25	core
group_5591	MFS transporter	25	core
flaC	flagellin C	25	core
rfaH	transcription antitermination protein RfaH	25	core
pdxA	4-hydroxythreonine-4-phosphate dehydrogenase	25	core
group_5595	histidine kinase	25	core
group_5596	hypothetical protein	25	core
group_5597	hypothetical protein	25	core
group_5598	DNA internalization-related competence protein ComEC/Rec2	25	core
astD	N-succinylglutamate 5-semialdehyde dehydrogenase	25	core
group_56	D-alanine--poly(phosphoribitol) ligase	25	core
group_560	riboflavin biosynthesis protein RibD	25	core
group_5600	aminodeoxychorismate lyase	25	core
group_5601	DNA-binding transcriptional regulator DsdC	25	core
group_5602	hypothetical protein	25	core
group_5603	Crp/Fnr family transcriptional regulator	25	core
group_5604	hypothetical protein	25	core
glpQ	glycerophosphodiester phosphodiesterase	25	core
group_5606	hypothetical protein	25	core
group_5607	hypothetical protein	25	core
group_5608	antibiotic biosynthesis monooxygenase	25	core
hlyB	methyl-accepting chemotaxis protein HlyB	25	core
fabZ	3-hydroxyacyl-[acyl-carrier-protein] dehydratase FabZ	25	core
group_5610	two-component sensor histidine kinase	25	core
group_5611	RNA-binding protein	25	core
group_5612	hypothetical protein	25	core
group_5613	hypothetical protein	25	core
pilC	type IV pilin assembly protein PilC	25	core
lolB	outer-membrane lipoprotein LolB	25	core
cobT	nicotinate-nucleotide--dimethylbenzimidazole phosphoribosyltransferase	25	core
group_5617	ribosomal-protein-alanine acetyltransferase	25	core
group_5618	transcriptional regulator	25	core
group_5619	peptide ABC transporter ATP-binding protein	25	core
rnhB	ribonuclease HII	25	core

fumC	fumarate hydratase class II	25	core
group_5621	6-phospho-beta-glucosidase	25	core
group_5622	1-acyl-sn-glycerol-3-phosphate acyltransferase	25	core
gcp	DNA-binding/iron metalloprotein/AP endonuclease	25	core
group_5624	ferredoxin	25	core
truB	tRNA pseudouridine synthase B	25	core
gshA	glutamate--cysteine ligase	25	core
group_5627	UPF0149 protein	25	core
group_5628	amino acid ABC transporter substrate-binding protein	25	core
group_5629	hypothetical protein	25	core
group_563	UPF0294 protein	25	core
gmhB	D-glycero-beta-D-manno-heptose-1,7-bisphosphate 7-phosphatase	25	core
ruvA	Holliday junction ATP-dependent DNA helicase RuvA	25	core
group_5632	thiamine kinase	25	core
astE	succinylglutamate desuccinylase	25	core
glnE	glutamate-ammonia-ligase adenylyltransferase	25	core
metQ	putative D-methionine-binding lipoprotein MetQ	25	core
group_5636	GntP protein	25	core
group_5637	malate synthase	25	core
group_5638	hypothetical protein	25	core
group_5639	paraquat-inducible protein A	25	core
group_564	glutamate synthase	25	core
group_5640	hypothetical protein	25	core
group_5641	oxaloacetate decarboxylase	25	core
group_5642	PAS domain-containing sensor histidine kinase	25	core
group_5643	diguanylate cyclase	25	core
group_5644	UPF0721 transmembrane protein	25	core
group_5645	ADP-ribose pyrophosphatase	25	core
group_5646	hypothetical protein	25	core
group_5647	acetolactate synthase	25	core
group_5648	LysR family transcriptional regulator	25	core
group_5649	heme exporter protein D	25	core
group_565	class II glutamine amidotransferase	25	core
group_5650	hypothetical protein	25	core
group_5651	acyltransferase	25	core
group_5652	benzoate transporter	25	core
leuD	3-isopropylmalate dehydratase small subunit	25	core
group_5654	gluconokinase	25	core
group_5655	MFS transporter	25	core

group_5656	peptide ABC transporter permease	25	core
group_5657	aromatic amino acid aminotransferase	25	core
group_5658	UPF0270 protein	25	core
group_5659	DUF1289 domain-containing protein	25	core
gltX	glutamate--tRNA ligase	25	core
leuB	3-isopropylmalate dehydrogenase	25	core
group_5661	UPF0319 protein	25	core
lpxB	lipid-A-disaccharide synthase	25	core
group_5663	aminoacyl-histidine dipeptidase	25	core
group_5664	ATPase	25	core
group_5665	hypothetical protein	25	core
trmA	tRNA/tmRNA (uracil-C(5))-methyltransferase	25	core
group_5667	ABC transporter substrate-binding protein	25	core
group_5668	pilus assembly protein PilM	25	core
pth	peptidyl-tRNA hydrolase	25	core
flgI	flagellar P-ring protein	25	core
lapA	putative lipopolysaccharide assembly protein A	25	core
kbl	2-amino-3-ketobutyrate coenzyme A ligase	25	core
group_5672	phage shock protein A	25	core
group_5673	arginine ABC transporter ATP-binding protein	25	core
group_5674	acetyltransferase	25	core
hutU	urocanate hydratase	25	core
torD	chaperone protein TorD	25	core
irgA	iron-regulated outer membrane virulence protein	25	core
group_5678	UPF0255 protein	25	core
gmk	guanylate kinase	25	core
group_568	Trp repressor-binding protein	25	core
fliL	flagellar basal body-associated protein FliL	25	core
group_5681	haloacid dehalogenase	25	core
group_5682	MFS transporter	25	core
group_5683	nicotinamidase	25	core
group_5684	hypothetical protein	25	core
ftsK	DNA translocase FtsK	25	core
toxR	cholera toxin transcriptional activator	25	core
group_5687	membrane protein	25	core
group_5688	xanthine permease	25	core
group_5689	histidine kinase	25	core
group_569	arsenate reductase	25	core
group_5690	UPF0304 protein	25	core

group_5691	methyl-accepting chemotaxis protein	25	core
group_5692	metalloprotease PmbA	25	core
group_5693	lipoprotein NlpI	25	core
group_5694	phage shock protein operon transcriptional activator	25	core
argD	acetylornithine aminotransferase	25	core
group_5696	tungsten ABC transporter substrate-binding protein	25	core
group_5697	transcriptional regulator	25	core
group_5698	hemolysin D	25	core
topB	DNA topoisomerase 3	25	core
group_57	LacI family transcriptional regulator	25	core
bcp	peroxiredoxin	25	core
arcA	arginine deiminase	25	core
group_5701	haloacid dehalogenase	25	core
group_5702	lysine transporter LysE	25	core
rne	ribonuclease E	25	core
group_5704	membrane protein	25	core
mutM	formamidopyrimidine-DNA glycosylase	25	core
prpB	2-methylisocitrate lyase	25	core
group_5707	ABC transporter ATP-binding protein	25	core
group_5708	oxidoreductase	25	core
group_5709	hemolysin D	25	core
group_571	hypothetical protein	25	core
group_5710	putative pre-16S rRNA nuclease	25	core
group_5711	multidrug resistance protein	25	core
group_5712	aromatic amino acid aminotransferase	25	core
group_5713	UPF0345 protein	25	core
group_5714	UPF0352 protein	25	core
rrmA	23S rRNA (guanine(745)-N(1))-methyltransferase	25	core
pabC	aminodeoxychorismate lyase	25	core
lptA	lipopolysaccharide export system protein LptA	25	core
group_5718	alpha-galactosidase	25	core
group_5719	iron(III) ABC transporter substrate-binding protein	25	core
group_572	arsenate reductase	25	core
group_5720	cyclopropane-fatty-acyl-phospholipid synthase	25	core
group_5721	multidrug ABC transporter ATP-binding protein	25	core
phhA	phenylalanine 4-monooxygenase	25	core
group_5723	hypothetical protein	25	core
gcvP	glycine dehydrogenase (decarboxylating)	25	core
group_5725	type VI secretion lipoprotein	25	core

group_5726	chemotaxis protein CheA	25	core
group_5727	chromosome segregation ATPase	25	core
malK	maltose/maltodextrin import ATP-binding protein MalK	25	core
group_5729	RNA polymerase sigma factor	25	core
group_5730	sensor histidine kinase	25	core
ddl	D-alanine--D-alanine ligase	25	core
holA	DNA polymerase III subunit delta	25	core
rlmG	ribosomal RNA large subunit methyltransferase G	25	core
group_5734	hypothetical protein	25	core
rimO	ribosomal protein S12 methylthiotransferase RimO	25	core
metN	methionine import ATP-binding protein MetN	25	core
group_5737	carbon starvation protein A	25	core
metI	putative D-methionine transport system permease protein MetI	25	core
group_5739	alpha-ribazole-5'-phosphate phosphatase	25	core
group_574	flagellar protein FliT	25	core
hisG	ATP phosphoribosyltransferase	25	core
group_5741	two-component sensor histidine kinase	25	core
group_5742	hypothetical protein	25	core
vibE	vibriobactin-specific 2,3-dihydroxybenzoate-AMP ligase	25	core
group_5744	oligopeptidase A	25	core
group_5745	6-phosphogluconolactonase	25	core
group_5746	electron transport complex subunit G	25	core
group_5747	DNAse	25	core
group_5748	thiol reductase thioredoxin	25	core
group_5749	hypothetical protein	25	core
fliJ	flagellar protein FliJ	25	core
artP	arginine transporter ATP-binding subunit	25	core
xseA	exodeoxyribonuclease 7 large subunit	25	core
group_5752	thermostable hemolysin	25	core
nhaP2	K(+)/H(+) antiporter NhaP2	25	core
cpxP	periplasmic repressor CpxP	25	core
tatC	Sec-independent protein translocase protein TatC	25	core
group_5756	hypothetical protein	25	core
group_5757	AraC family transcriptional regulator	25	core
group_5758	membrane protein	25	core
hutI	imidazolonepropionase	25	core
group_576	hypothetical protein	25	core
luxU	phosphorelay protein LuxU	25	core
group_5761	hypothetical protein	25	core

murG	UDP-N-acetylglucosamine--N-acetylmuramyl-(pentape ptide) pyrophosphoryl-undecaprenol N-acetylglucosamine transferase	25	core
group_5763	heme exporter protein C	25	core
vmrA	MATE family efflux transporter	25	core
group_5765	PTS ascorbate transporter subunit IIA	25	core
group_5766	UPF0758 protein	25	core
group_5767	phosphatidylglycerophosphatase B	25	core
lpxL	lipid A biosynthesis lauroyltransferase	25	core
thrB	homoserine kinase	25	core
group_577	LysR family transcriptional regulator	25	core
udk	uridine kinase	25	core
group_5771	short-chain dehydrogenase/reductase	25	core
group_5772	methyl-accepting chemotaxis protein	25	core
def2	peptide deformylase 2	25	core
group_5774	hypothetical protein	25	core
group_5775	glutathione-dependent reductase	25	core
tbpA	thiamine transporter substrate binding subunit	25	core
group_5777	hypothetical protein	25	core
group_5778	membrane protein	25	core
lpxM	lipid A biosynthesis myristoyltransferase	25	core
group_578	CidA/LrgA family protein	25	core
group_5780	XRE family transcriptional regulator	25	core
group_5781	histidine kinase	25	core
glpE	thiosulfate sulfurtransferase GlpE	25	core
group_5783	hypothetical protein	25	core
group_5784	TIGR01212 family radical SAM protein	25	core
truA	tRNA pseudouridine synthase A	25	core
zntA	zinc/cadmium/mercury/lead-transporting ATPase	25	core
group_5787	hypothetical protein	25	core
group_5788	inorganic triphosphatase	25	core
group_5789	hypothetical protein	25	core
group_579	zinc ABC transporter substrate-binding protein	25	core
group_5790	hypothetical protein	25	core
group_5791	formate dehydrogenase subunit gamma	25	core
group_5792	Fe3+-hydroxamate ABC transporter permease FhuB	25	core
group_5793	nicotinate-nucleotide diphosphorylase	25	core
viuA	vibriobactin receptor	25	core
group_5795	GTP cyclohydrolase 1 type 2	25	core
group_5796	sigma-54-dependent Fis family transcriptional regulator	25	core
rnpA	ribonuclease P protein component	25	core

group_5798	UPF0176 protein	25	core
rsmA	ribosomal RNA small subunit methyltransferase A	25	core
group_58	sigma-54-dependent Fis family transcriptional regulator	25	core
group_580	phosphohistidine phosphatase SixA	25	core
torA	trimethylamine-N-oxide reductase	25	core
group_5801	twitching motility protein PilT	25	core
group_5802	serine/threonine protein kinase	25	core
group_5803	hypothetical protein	25	core
secG	preprotein translocase subunit SecG	25	core
recB	RecBCD enzyme subunit RecB	25	core
group_5806	sigma-54-dependent Fis family transcriptional regulator	25	core
group_5807	hypothetical protein	25	core
group_5808	hypothetical protein	25	core
anmK	anhydro-N-acetylmuramic acid kinase	25	core
group_581	short-chain dehydrogenase	25	core
group_5810	stringent starvation protein B	25	core
group_5811	hypothetical protein	25	core
group_5812	exopolyphosphatase	25	core
group_5813	histidine kinase	25	core
group_5814	tRNA (adenosine(37)-N6)-threonylcarbamoyltransferase complex dimerization subunit type 1 TsaB	25	core
group_5815	membrane protein	25	core
group_5816	hypothetical protein	25	core
group_5817	ATPase	25	core
group_5818	hypothetical protein	25	core
group_5819	citrate:sodium symporter	25	core
group_582	sensor histidine kinase	25	core
group_5820	DNA-binding transcriptional activator CadC	25	core
group_5821	amidohydrolase	25	core
hemD	uroporphyrinogen-III synthase	25	core
group_5823	SAM-dependent methyltransferase	25	core
group_5824	haloacid dehalogenase	25	core
group_5825	acetoacetyl-CoA synthetase	25	core
group_5826	UPF0502 protein	25	core
group_5827	peptidase S9	25	core
ispE	4-diphosphocytidyl-2-C-methyl-D-erythritol kinase	25	core
ispD	2-C-methyl-D-erythritol 4-phosphate cytidylyltransferase	25	core
rluC	ribosomal large subunit pseudouridine synthase C	25	core
hslV	ATP-dependent protease subunit HslV	25	core
group_5831	hypothetical protein	25	core

group_5832	competence protein ComEA	25	core
group_5833	MarR family transcriptional regulator	25	core
mutS	DNA mismatch repair protein MutS	25	core
group_5835	sensor histidine kinase	25	core
purK	N5-carboxyaminoimidazole ribonucleotide synthase	25	core
hemH	ferrochelatase	25	core
group_5838	allophanate hydrolase	25	core
group_5839	hypothetical protein	25	core
group_584	DNA polymerase III subunit delta'	25	core
group_5840	hypothetical protein	25	core
group_5841	potassium channel protein	25	core
group_5842	UPF0181 protein	25	core
group_5843	phosphatase PAP2 family protein	25	core
group_5844	transporter	25	core
group_5845	helix-turn-helix transcriptional regulator	25	core
group_5846	Maf-like protein	25	core
group_5847	VOC family protein	25	core
tsaC	threonylcarbamoyl-AMP synthase	25	core
glsA	glutaminase	25	core
group_585	hypothetical protein	25	core
hldD	ADP-L-glycero-D-manno-heptose-6-epimerase	25	core
group_5851	putative quercetin 2,3-dioxygenase	25	core
mutH	DNA mismatch repair protein MutH	25	core
group_5853	photosynthetic protein synthase I	25	core
group_5854	hypothetical protein	25	core
group_5855	cytochrome oxidase maturation protein Cbb3	25	core
btuF	vitamin B12-binding protein	25	core
group_5857	hypothetical protein	25	core
group_5858	hypothetical protein	25	core
group_5859	hypothetical protein	25	core
group_586	ATP-dependent helicase	25	core
cysA	sulfate/thiosulfate import ATP-binding protein CysA	25	core
group_5861	diguanylate phosphodiesterase	25	core
mukF	chromosome partition protein MukF	25	core
group_5863	MSHA biogenesis protein MshJ	25	core
cysC	adenylyl-sulfate kinase	25	core
pyrB	aspartate carbamoyltransferase	25	core
group_5866	GntR family transcriptional regulator	25	core
group_5867	helicase-like protein	25	core

purH	bifunctional purine biosynthesis protein PurH	25	core
pyrI	aspartate carbamoyltransferase regulatory chain	25	core
group_587	alpha/beta hydrolase	25	core
group_5870	AraC family transcriptional regulator	25	core
group_5871	Na+/H+ antiporter subunit G	25	core
group_5872	pilus assembly protein PilB	25	core
dns	extracellular deoxyribonuclease	25	core
kdsB	3-deoxy-manno-octulosonate cytidylyltransferase	25	core
modB	molybdate ABC transporter permease	25	core
group_5876	hypothetical protein	25	core
group_5877	bacterioferritin-associated ferredoxin	25	core
tusD	sulfurtransferase TusD	25	core
group_5879	transcriptional regulatory protein	25	core
group_588	DTW domain-containing protein	25	core
group_5880	peptidase M15	25	core
group_5881	L-serine ammonia-lyase	25	core
group_5882	TMAO reductase system periplasmic protein TorT	25	core
ugpE	sn-glycerol-3-phosphate transport system permease protein UgpE	25	core
group_5884	hypothetical protein	25	core
group_5885	transcriptional regulator	25	core
group_5886	phosphoserine phosphatase	25	core
thiC	phosphomethylpyrimidine synthase	25	core
group_5888	hypothetical protein	25	core
pdxB	erythronate-4-phosphate dehydrogenase	25	core
menH	putative 2-succinyl-6-hydroxy-2,4-cyclohexadiene-1-carboxylate synthase	25	core
cobU	adenosylcobinamide kinase/adenosylcobinamide phosphate guanyltransferase	25	core
crl	sigma factor-binding protein Crl	25	core
group_5892	dUMP phosphatase	25	core
group_5893	glycosyl transferase	25	core
group_5894	cytochrome c biogenesis protein CcdA	25	core
birA	bifunctional ligase/repressor BirA	25	core
yacG	DNA gyrase inhibitor YacG	25	core
group_5897	transport permease protein	25	core
aroB	3-dehydroquinate synthase	25	core
bamA	outer membrane protein assembly factor BamA	25	core
group_59	hypothetical protein	25	core
group_590	lytic transglycosylase	25	core
group_5900	membrane protein	25	core
group_5901	histidine utilization repressor	25	core

tyrS2	tyrosine--tRNA ligase 2	25	core
crcB	putative fluoride ion transporter CrcB	25	core
group_5904	LacI family transcriptional regulator	25	core
group_5905	gamma carbonic anhydrase family protein	25	core
cysI	sulfite reductase [NADPH] hemoprotein beta-component	25	core
group_5907	hypothetical protein	25	core
epsN	type II secretion system protein N	25	core
group_5909	cytochrome ubiquinol oxidase subunit I	25	core
group_591	cytochrome c-type protein	25	core
def1	peptide deformylase 1	25	core
group_5911	DNA-binding response regulator	25	core
group_5912	UPF0325 protein	25	core
group_5913	polyphosphate kinase	25	core
group_5914	5-carboxymethyl-2-hydroxymuconate isomerase	25	core
group_5915	membrane protein	25	core
lptC	lipopolysaccharide export system protein LptC	25	core
group_5917	haloacid dehalogenase	25	core
truD	tRNA pseudouridine synthase D	25	core
group_5919	hypothetical protein	25	core
group_5920	lysoplasmalogenase	25	core
group_5921	transcriptional regulator	25	core
malF	maltose transport system permease protein MalF	25	core
group_5923	thiosulfate transporter subunit	25	core
group_5924	branched-chain amino acid ABC transporter	25	core
group_5925	hypothetical protein	25	core
ugpC	sn-glycerol-3-phosphate import ATP-binding protein UgpC	25	core
group_5927	DsbA family protein	25	core
group_5928	gamma-glutamylcyclotransferase	25	core
recX	regulatory protein RecX	25	core
group_593	UPF0061 protein	25	core
nqrC	Na(+)-translocating NADH-quinone reductase subunit C	25	core
group_5931	recombinase RarA	25	core
group_5932	citrate lyase subunit alpha	25	core
valS	valine--tRNA ligase	25	core
group_5934	DNA topoisomerase	25	core
group_5935	chemotaxis protein	25	core
group_5936	2-polyprenyl-3-methyl-5-hydroxy-6-metoxy-1,4-benz oquinol methylase	25	core
group_5937	peptidyl-prolyl cis-trans isomerase	25	core
group_5938	hypothetical protein	25	core

group_5939	hypothetical protein	25	core
dctQ	C4-dicarboxylate TRAP transporter small permease protein DctQ	25	core
group_5940	membrane protein	25	core
group_5941	aminopeptidase	25	core
group_5942	multidrug transporter AcrB	25	core
group_5943	membrane protein	25	core
glpB	anaerobic glycerol-3-phosphate dehydrogenase subunit B	25	core
group_5945	hypothetical protein	25	core
tusC	protein TusC	25	core
group_5947	ATPase AAA	25	core
group_5948	hypothetical protein	25	core
group_5949	hypothetical protein	25	core
pyrF	orotidine 5'-phosphate decarboxylase	25	core
group_5950	hypothetical protein	25	core
group_5951	glyoxalase	25	core
group_5952	RDD family protein	25	core
group_5953	multidrug transporter AcrB	25	core
group_5954	hypothetical protein	25	core
group_5955	N-acetyltransferase	25	core
group_5956	(Fe-S)-binding protein	25	core
epsC	type II secretion system protein C	25	core
group_5958	general secretion pathway protein GspA	25	core
group_5959	hypothetical protein	25	core
group_596	tRNA-(ms[2]io[6]A)-hydroxylase	25	core
group_5960	hypothetical protein	25	core
group_5961	RNA methyltransferase	25	core
group_5962	cysteine/glutathione ABC transporter ATP-binding protein/permease CydC	25	core
group_5963	pyruvate kinase	25	core
group_5964	hypothetical protein	25	core
group_5965	GGDEF domain-containing protein	25	core
group_5966	diguanylate cyclase	25	core
psd	phosphatidylserine decarboxylase proenzyme	25	core
group_5968	hypothetical protein	25	core
tdk	thymidine kinase	25	core
lpxK	tetraacyldisaccharide 4'-kinase	25	core
group_5970	allophanate hydrolase	25	core
csd	putative cysteine desulfurase	25	core
group_5972	putative heme utilization radical SAM enzyme HutW	25	core
group_5973	N-acetyltransferase	25	core

group_5974	trimethylamine N-oxide reductase system protein TorE	25	core
group_5975	hypothetical protein	25	core
hutH	histidine ammonia-lyase	25	core
rmf	ribosome modulation factor	25	core
coaA	pantothenate kinase	25	core
glnL	two-component system sensor histidine kinase NtrB	25	core
group_598	membrane protein	25	core
moaE	molybdopterin synthase catalytic subunit	25	core
group_5981	hypothetical protein	25	core
group_5982	[Citrate [pro-3S]-lyase] ligase	25	core
ubiE	ubiquinone/menaquinone biosynthesis C-methyltransferase UbiE	25	core
nagZ	beta-hexosaminidase	25	core
group_5985	acyl-CoA esterase	25	core
group_5986	hypothetical protein	25	core
group_5987	LysR family transcriptional regulator	25	core
group_5988	putative pterin-4-alpha-carbinolamine dehydratase	25	core
group_5989	patatin family protein	25	core
group_5990	membrane protein	25	core
group_5991	Fe-S assembly protein IscX	25	core
group_5992	phosphatidylglycerophosphatase A	25	core
rnc	ribonuclease 3	25	core
group_5994	type IV pilus biogenesis/stability protein PilW	25	core
msrA	peptide methionine sulfoxide reductase MsrA	25	core
group_5996	phosphate ABC transporter substrate-binding protein	25	core
group_5997	NADH dehydrogenase subunit L	25	core
group_5998	DUF490 domain-containing protein	25	core
group_5999	two-component sensor histidine kinase	25	core
group_6	methyl-accepting chemotaxis protein	25	core
group_60	thioredoxin family protein	25	core
group_600	histidine kinase	25	core
group_6000	MBL fold metallo-hydrolase	25	core
glgA	glycogen synthase	25	core
group_6002	multidrug transporter	25	core
group_6003	biliverdin-producing heme oxygenase	25	core
group_6004	thiol:disulfide oxidoreductase	25	core
group_6005	acetyl-CoA carboxylase biotin carboxylase subunit	25	core
cpdB	bifunctional 2',3'-cyclic nucleotide 2'-phosphodiesterase/3'-nucleotidase	25	core
menD	2-succinyl-5-enolpyruvyl-6-hydroxy-3-cyclohexene- 1-carboxylate synthase	25	core
group_601	hypothetical protein	25	core

group_602	cytochrome d ubiquinol oxidase subunit II	25	core
group_603	hypothetical protein	25	core
group_605	nuclease	25	core
group_609	hemolysin secretion protein D	25	core
group_611	diguanylate phosphodiesterase	25	core
group_612	nucleoside-diphosphate sugar epimerase	25	core
group_613	DNA-binding protein	25	core
group_614	cytochrome c-type protein	25	core
group_615	hypothetical protein	25	core
group_616	cystathionine beta-lyase	25	core
group_617	membrane protein	25	core
group_618	ABC transporter substrate-binding protein	25	core
group_619	membrane protein	25	core
group_62	hypothetical protein	25	core
group_621	haloacid dehalogenase	25	core
group_622	hypothetical protein	25	core
rsuA	ribosomal small subunit pseudouridine synthase A	25	core
group_625	hypothetical protein	25	core
group_626	membrane protein	25	core
group_628	amino acid ABC transporter substrate-binding protein	25	core
group_63	GGDEF domain-containing protein	25	core
group_630	GGDEF domain-containing protein	25	core
group_631	galactose-1-phosphate uridylyltransferase	25	core
group_635	hypothetical protein	25	core
group_636	hypothetical protein	25	core
group_637	ABC transporter ATP-binding protein	25	core
group_638	two-component sensor histidine kinase	25	core
group_639	ABC-F family ATPase	25	core
group_64	hydroxymethylpyrimidine/phosphomethylpyrimidine kinase	25	core
group_640	formate dehydrogenase accessory protein	25	core
group_641	formate dehydrogenase	25	core
group_642	formate dehydrogenase iron-sulfur subunit	25	core
group_643	hypothetical protein	25	core
group_644	hypothetical protein	25	core
rsmF	ribosomal RNA small subunit methyltransferase F	25	core
group_646	hypothetical protein	25	core
rtxC	cytolysin-activating lysine-acyltransferase RtxC	25	core
group_648	histidine kinase	25	core
group_649	Cbb3-type cytochrome c oxidase subunit	25	core

bioA	adenosylmethionine-8-amino-7-oxononanoate aminotransferase	25	core
group_653	ATP-dependent RNA helicase HrpA	25	core
group_654	hypothetical protein	25	core
group_656	hypothetical protein	25	core
group_657	molecular chaperone DnaK	25	core
group_660	hypothetical protein	25	core
group_661	amino acid ABC transporter permease	25	core
rlmI	ribosomal RNA large subunit methyltransferase I	25	core
group_664	putative dioxygenase	25	core
group_665	hypothetical protein	25	core
group_666	hypothetical protein	25	core
group_667	hypothetical protein	25	core
group_668	methyl-accepting chemotaxis protein	25	core
group_669	ribosomal protein S5 alanine N-acetyltransferase	25	core
group_670	UPF0283 membrane protein	25	core
group_671	aminodeoxychorismate synthase, component I	25	core
group_672	methyl-accepting chemotaxis protein	25	core
group_675	carbohydrate deacetylase	25	core
group_676	C4-dicarboxylate ABC transporter	25	core
group_677	C4-dicarboxylate ABC transporter	25	core
group_678	ABC transporter substrate-binding protein	25	core
group_679	sugar efflux transporter SetB	25	core
group_68	ABC transporter permease	25	core
ubiG	ubiquinone biosynthesis O-methyltransferase	25	core
tpm	thiopurine S-methyltransferase	25	core
group_682	N-acetyltransferase	25	core
group_683	elongation factor P-like protein	25	core
group_684	UPF0263 protein	25	core
group_686	hypothetical protein	25	core
group_687	hypothetical protein	25	core
group_688	SanA protein	25	core
group_689	thiol reductant ABC exporter subunit CydD	25	core
group_69	ABC transporter permease	25	core
trpD	anthranilate phosphoribosyltransferase	25	core
trpCF	tryptophan biosynthesis protein TrpCF	25	core
trpA	tryptophan synthase alpha chain	25	core
group_693	proton/glutamate symporter	25	core
group_694	hypothetical protein	25	core
cspD	cold shock-like protein CspD	25	core

rluE	hypothetical protein	25	core
group_697	DUF1365 domain-containing protein	25	core
htpX	protease HtpX	25	core
dusC	tRNA-dihydrouridine(16) synthase	25	core
cry1	hypothetical protein	25	core
group_70	vibriobactin synthetase	25	core
group_700	two-component system response regulator	25	core
group_701	threonylcarbamoyl-AMP synthase	25	core
group_702	chitinase	25	core
group_703	glyceraldehyde-3-phosphate dehydrogenase	25	core
group_704	UPF0153 protein	25	core
group_705	DNA polymerase III subunit gamma/tau	25	core
group_706	aromatic hydrocarbon degradation protein	25	core
group_708	cob(I)yrinic acid a,c-diamide adenosyltransferase	25	core
metG	methionine--tRNA ligase	25	core
vibA	vibriobactin-specific 2,3-dihydro-2,3-dihydroxybenzoate dehydrogenase	25	core
group_710	GGDEF domain-containing protein	25	core
moaD	molybdopterin synthase sulfur carrier subunit	25	core
luxO	regulatory protein LuxO	25	core
group_713	membrane protein	25	core
group_714	potassium transporter Kef	25	core
htpG	chaperone protein HtpG	25	core
group_716	hypothetical protein	25	core
cueR	HTH-type transcriptional regulator CueR	25	core
cysZ	sulfate transporter CysZ	25	core
group_72	membrane protein	25	core
group_720	2-octaprenyl-3-methyl-6-methoxy-1,4-benzoquinol hydroxylase	25	core
group_721	hypothetical protein	25	core
group_723	teicoplanin resistance protein VanZ	25	core
group_724	hypothetical protein	25	core
group_725	undecaprenyl-phosphate glucose phosphotransferase	25	core
queF	NADPH-dependent 7-cyano-7-deazaguanine reductase	25	core
group_729	hypothetical protein	25	core
xni	Flap endonuclease Xni	25	core
group_731	DUF423 domain-containing protein	25	core
group_732	flagellar motor protein PomA	25	core
xseB	exodeoxyribonuclease 7 small subunit	25	core
group_734	tRNA (N6-threonylcarbamoyladenosine(37)-N6)-methyltransferase TrmO	25	core
group_735	hypothetical protein	25	core

recN	DNA repair protein RecN	25	core
citG	putative 2-(5"-triphosphoribosyl)-3'-dephosphocoenzyme-A synthase	25	core
citX	putative apo-citrate lyase phosphoribosyl-dephospho-CoA transferase	25	core
citD	citrate lyase acyl carrier protein	25	core
group_749	ATPase	25	core
group_751	sodium:alanine symporter	25	core
group_756	hypothetical protein	25	core
hisS	histidine--tRNA ligase	25	core
group_758	acetoin utilization protein AcuB	25	core
cutC	copper homeostasis protein CutC	25	core
group_76	diguanylate phosphodiesterase	25	core
group_760	laccase domain protein	25	core
trpR	Trp operon repressor	25	core
group_762	pilus protein PilZ	25	core
prfC	peptide chain release factor 3	25	core
group_764	DNA polymerase III subunit psi	25	core
group_765	SCP2 domain-containing protein	25	core
group_766	protease	25	core
rlmE	ribosomal RNA large subunit methyltransferase E	25	core
group_768	ammonium transporter	25	core
sfsA	sugar fermentation stimulation protein	25	core
group_771	lipoprotein	25	core
group_772	UPF0102 protein	25	core
group_773	aspartokinase	25	core
group_774	competence damage-inducible protein A	25	core
group_780	DeoR family transcriptional regulator	25	core
group_781	pyruvate kinase	25	core
group_782	mechanosensitive ion channel protein	25	core
sprT	protein SprT	25	core
tyrS1	tyrosine--tRNA ligase 1	25	core
group_785	helix-turn-helix transcriptional regulator	25	core
group_787	UPF0001 protein	25	core
group_788	UPF0235 protein	25	core
group_789	hypothetical protein	25	core
group_790	non-canonical purine NTP pyrophosphatase	25	core
group_791	YggW family oxidoreductase	25	core
apaH	bis(5'-nucleosyl)-tetraphosphatase, symmetrical	25	core
group_793	dihydrofolate reductase	25	core
group_794	arginine:ornithine antiporter	25	core

group_797	MSHA pilin protein MshB	25	core
group_799	RNase E specificity factor CsrD	25	core
res	type III restriction-modification system restriction subunit Res	25	core
ssb	single-stranded DNA-binding protein	25	core
group_801	UTP--glucose-1-phosphate uridylyltransferase	25	core
cysH	phosphoadenosine phosphosulfate reductase	25	core
group_803	hypothetical protein	25	core
hflX	GTPase HflX	25	core
hfq	RNA-binding protein Hfq	25	core
miaA	tRNA dimethylallyltransferase	25	core
mutL	DNA mismatch repair protein MutL	25	core
orn	oligoribonuclease	25	core
cqsS	CAI-1 autoinducer sensor kinase/phosphatase CqsS	25	core
rsgA	putative ribosome biogenesis GTPase RsgA	25	core
group_811	membrane protein	25	core
group_812	peptidase M23	25	core
group_813	hypothetical protein	25	core
group_814	TetR family transcriptional regulator	25	core
murB	UDP-N-acetylenolpyruvoylglucosamine reductase	25	core
fre	NAD(P)H-flavin reductase	25	core
group_817	transcriptional regulator	25	core
group_818	peptidase	25	core
zntR	heavy metal-responsive transcriptional regulator	25	core
group_820	2-ketoacid reductase	25	core
group_822	MBL fold hydrolase	25	core
group_83	ammonia monooxygenase	25	core
group_834	glycosyl transferase	25	core
rpmB	50S ribosomal protein L28	25	core
rph	ribonuclease PH	25	core
murQ1	N-acetylmuramic acid 6-phosphate etherase 1	25	core
group_839	XRE family transcriptional regulator	25	core
group_84	DNA mismatch repair protein MutT	25	core
group_840	iron(III) ABC transporter ATP-binding protein	25	core
group_841	putative transporter	25	core
group_842	threonine transporter	25	core
group_843	sporulation protein	25	core
hemG	protoporphyrinogen oxidase	25	core
group_845	YigZ family protein	25	core
glmU	bifunctional protein GlmU	25	core

atpI	ATP synthase protein I	25	core
group_85	putative sucrose-6-phosphate hydrolase	25	core
group_851	pyridoxamine 5'-phosphate oxidase	25	core
group_852	3-hydroxyisobutyrate dehydrogenase	25	core
group_853	4-alpha-glucanotransferase	25	core
group_856	glutathione S-transferase	25	core
group_857	transporter	25	core
group_858	methyl-accepting chemotaxis protein	25	core
group_859	hypothetical protein	25	core
group_86	two-component sensor histidine kinase	25	core
group_860	hypothetical protein	25	core
group_861	helix-turn-helix-type transcriptional regulator	25	core
group_862	hypothetical protein	25	core
group_863	phosphate ABC transporter permease	25	core
group_864	oxidoreductase	25	core
group_865	membrane protein	25	core
group_866	peptidase M23	25	core
group_867	MFS transporter	25	core
group_868	MFS transporter	25	core
group_869	hypothetical protein	25	core
cobQ	cobyric acid synthase	25	core
group_870	hypothetical protein	25	core
group_871	hypothetical protein	25	core
group_872	hypothetical protein	25	core
group_873	hypothetical protein	25	core
group_875	C4-dicarboxylate ABC transporter permease	25	core
group_878	hypothetical protein	25	core
group_879	GGDEF domain-containing protein	25	core
group_88	serine protease	25	core
group_880	alternative ribosome-rescue factor A	25	core
group_881	hypothetical protein	25	core
group_882	sodium:proton antiporter	25	core
rhlE	ATP-dependent RNA helicase RhlE	25	core
group_885	hypothetical protein	25	core
hlyA	hemolysin	25	core
lipA_1	lipase	25	core
group_889	protease	25	core
group_89	membrane protein	25	core
group_893	DNA-binding response regulator	25	core

group_894	cytochrome b	25	core
group_897	hypothetical protein	25	core
group_898	carbonic anhydrase	25	core
rpmI	50S ribosomal protein L35	25	core
group_9	hypothetical protein	25	core
group_90	metal-dependent phosphohydrolase	25	core
group_909	RelB protein	25	core
group_912	ribosomal protein S6 modification protein	25	core
sbcD	nuclease SbcCD subunit D	25	core
group_919	hypothetical protein	25	core
group_92	diguanylate cyclase	25	core
group_920	hypothetical protein	25	core
group_921	Zn-dependent protease	25	core
group_922	hypothetical protein	25	core
group_923	membrane protein	25	core
group_924	sensor domain-containing diguanylate cyclase	25	core
group_925	hypothetical protein	25	core
group_926	GGDEF domain-containing protein	25	core
group_927	sensor histidine kinase	25	core
group_931	hypothetical protein	25	core
group_932	hypothetical protein	25	core
group_933	glutathione S-transferase	25	core
group_934	TetR family transcriptional regulator	25	core
group_935	DNA mismatch repair protein MutT	25	core
group_936	hypothetical protein	25	core
group_937	multidrug transporter	25	core
group_942	molybdopterin biosynthesis MoeA protein	25	core
group_943	23S rRNA methyltransferase	25	core
group_944	membrane protein	25	core
group_945	hypothetical protein	25	core
group_946	glycerol-3-phosphate dehydrogenase	25	core
group_948	beta-ketoacyl-ACP reductase	25	core
group_949	diguanylate cyclase	25	core
group_95	diguanylate phosphodiesterase	25	core
group_950	chitinase	25	core
group_951	lysoplasmalogenase	25	core
group_952	diacylglycerol kinase	25	core
modC	molybdenum import ATP-binding protein ModC	25	core
group_954	hypothetical protein	25	core

group_956	arginine transporter permease subunit ArtQ	25	core
group_957	arginine ABC transporter substrate-binding protein	25	core
group_958	threonine aldolase	25	core
group_959	hypothetical protein	25	core
group_960	oxidoreductase	25	core
group_961	arylesterase	25	core
group_962	membrane protein	25	core
group_963	hypothetical protein	25	core
group_964	ABC transporter substrate-binding protein	25	core
group_965	hypothetical protein	25	core
ectA	L-2,4-diaminobutyric acid acetyltransferase	25	core
group_967	hypothetical protein	25	core
group_968	hypothetical protein	25	core
group_969	hypothetical protein	25	core
ubiC	putative chorismate pyruvate-lyase	25	core
group_981	VOC family protein	25	core
group_982	hypothetical protein	25	core
group_983	methyl-accepting chemotaxis protein	25	core
tonB	protein TonB	25	core
exbB1	biopolymer transport protein exbB1	25	core
group_986	hypothetical protein	25	core
pyrC	dihydroorotase	25	core
rlmC	23S rRNA (uracil(747)-C(5))-methyltransferase RlmC	25	core
group_99	flagellar basal body-associated protein FliL	25	core
malE	maltose ABC transporter substrate-binding protein	25	core
group_991	chemotaxis protein CheW	25	core
group_992	malate synthase	25	core
group_993	sensor domain-containing diguanylate cyclase	25	core
group_994	alkylated DNA repair protein	25	core
group_995	membrane protein	25	core
group_996	methyl-accepting chemotaxis protein	25	core
group_997	hypothetical protein	25	core
group_998	L-lactate permease	25	core
group_999	phosphoenolpyruvate synthase	25	core
group_1010	amino acid ABC transporter permease	24	accessory
mtlR	MltR family transcriptional regulator	24	accessory
group_1012	oxidoreductase	24	accessory
group_1013	UPF0721 transmembrane protein	24	accessory
group_1014	LysR family transcriptional regulator	24	accessory

group_1015	hypothetical protein	24	accessory
group_105	hypothetical protein	24	accessory
group_115	hypothetical protein	24	accessory
group_1172	quinone oxidoreductase	24	accessory
ilvD	dihydroxy-acid dehydratase	24	accessory
group_1180	heme biosynthesis protein HemY	24	accessory
group_1182	3',5'-cyclic-nucleotide phosphodiesterase	24	accessory
group_1183	2-haloalkanoic acid dehalogenase	24	accessory
group_1195	GTP-binding protein	24	accessory
group_1197	hypothetical protein	24	accessory
group_1206	ABC transporter ATP-binding protein	24	accessory
group_1207	isoaspartyl peptidase/L-asparaginase	24	accessory
rpsM	30S ribosomal protein S13	24	accessory
group_121	hypothetical protein	24	accessory
group_1210	putative gamma-glutamylcyclotransferase	24	accessory
group_1211	tRNA-Thr	24	accessory
nadB	L-aspartate oxidase	24	accessory
group_1217	putative HTH-type transcriptional regulator	24	accessory
group_1218	diguanylate cyclase	24	accessory
group_1223	methyl-accepting chemotaxis protein	24	accessory
ccmA	cytochrome c biogenesis ATP-binding export protein CcmA	24	accessory
asd1	aspartate-semialdehyde dehydrogenase 1	24	accessory
group_1234	malonyl CoA-acyl carrier protein transacylase	24	accessory
group_1251	SAM-dependent methyltransferase	24	accessory
group_1310	acyl-CoA dehydrogenase	24	accessory
group_133	molecular chaperone DnaK	24	accessory
group_1337	copper-translocating P-type ATPase	24	accessory
group_1338	cytochrome biogenesis protein	24	accessory
group_1341	transporter	24	accessory
group_1345	exonuclease	24	accessory
queC	7-cyano-7-deazaguanine synthase	24	accessory
group_1347	amino acid ABC transporter permease	24	accessory
group_1348	putative membrane protein	24	accessory
group_1350	acyl-CoA synthetase	24	accessory
group_1353	hypothetical protein	24	accessory
group_1354	cyclic nucleotide-binding protein	24	accessory
group_1355	permease IIC component	24	accessory
group_1356	BCCT family transporter	24	accessory
group_1358	membrane protein	24	accessory

trpE	anthranilate synthase component 1	24	accessory
group_1360	DNA-binding protein	24	accessory
group_1363	hypothetical protein	24	accessory
group_1364	electron transport complex subunit E	24	accessory
glnS	glutamine--tRNA ligase	24	accessory
group_1431	murein transglycosylase	24	accessory
hlyU	transcriptional activator HlyU	24	accessory
group_1436	hypoxanthine phosphoribosyltransferase	24	accessory
group_1437	peptidase M16	24	accessory
group_1439	hemolysin	24	accessory
group_1440	ABC transporter substrate-binding protein	24	accessory
proC	pyrroline-5-carboxylate reductase	24	accessory
lptD	LPS-assembly protein LptD	24	accessory
group_1453	membrane protein	24	accessory
group_1455	cyclic nucleotide-binding protein	24	accessory
group_1500	peptide ABC transporter permease	24	accessory
group_1517	hypothetical protein	24	accessory
group_1519	hypothetical protein	24	accessory
group_1528	iron(III) ABC transporter substrate-binding protein	24	accessory
group_1529	iron(III) ABC transporter permease	24	accessory
oadG2	hypothetical protein	24	accessory
group_1530	iron(III) ABC transporter permease	24	accessory
group_1531	iron(III) ABC transporter ATP-binding protein	24	accessory
group_1573	GTPase	24	accessory
msrAB	peptide methionine sulfoxide reductase MsrA/MsrB	24	accessory
group_1593	membrane protein	24	accessory
secF_1	protein-export membrane protein SecF	24	accessory
group_1602	3-hydroxy-3-methylglutaryl-CoA reductase	24	accessory
group_1655	acyltransferase	24	accessory
group_1657	lactate dehydrogenase	24	accessory
group_1661	N-formylglutamate amidohydrolase	24	accessory
group_1665	phospho-2-dehydro-3-deoxyheptonate aldolase	24	accessory
group_1666	polar amino acid ABC transporter permease	24	accessory
group_1667	amino acid ABC transporter substrate-binding protein	24	accessory
group_1668	phosphomannomutase	24	accessory
mtlA	PTS system mannitol-specific EIICBA component	24	accessory
group_1671	hypothetical protein	24	accessory
group_1672	hypothetical protein	24	accessory
group_1673	NAD-dependent dehydratase	24	accessory

group_1674	oxidoreductase	24	accessory
group_1675	LysR family transcriptional regulator	24	accessory
group_1676	putative pseudouridine methyltransferase	24	accessory
ribB_1	3,4-dihydroxy-2-butanone 4-phosphate synthase	24	accessory
potE	putrescine-ornithine antiporter	24	accessory
group_1679	ornithine decarboxylase SpeF	24	accessory
group_1680	aldehyde dehydrogenase	24	accessory
group_1681	AsnC family transcriptional regulator	24	accessory
group_1682	methyl-accepting chemotaxis protein	24	accessory
group_1683	sodium:proline symporter	24	accessory
group_1684	1-pyrroline-5-carboxylate dehydrogenase	24	accessory
group_1685	bifunctional proline dehydrogenase/pyrroline-5-carboxylate dehydrogenase	24	accessory
group_1686	AraC family transcriptional regulator	24	accessory
group_1692	sulfurtransferase	24	accessory
group_1701	chromosome segregation ATPase	24	accessory
group_1774	hypothetical protein	24	accessory
group_208	type II secretion system protein GspD	24	accessory
group_282	hemolysin D	24	accessory
acyP	acylphosphatase	24	accessory
group_288	GGDEF domain-containing protein	24	accessory
group_2917	methyl-accepting chemotaxis protein	24	accessory
group_30	transporter	24	accessory
group_342	miniconductance mechanosensitive channel	24	accessory
group_38	sensor histidine kinase	24	accessory
group_383	plasmid stabilization protein ParE	24	accessory
group_40	SAM-dependent methyltransferase	24	accessory
group_439	arginine ABC transporter ATP-binding protein	24	accessory
mtlD	mannitol-1-phosphate 5-dehydrogenase	24	accessory
group_441	hypothetical protein	24	accessory
group_501	heme biosynthesis operon protein HemX	24	accessory
ftsY	signal recognition particle receptor FtsY	24	accessory
group_5097	tRNA-Asp	24	accessory
group_51	flagellar hook-length control protein FliK	24	accessory
group_5342	hypothetical protein	24	accessory
group_61	RTX toxin transporter	24	accessory
group_629	hypothetical protein	24	accessory
group_632	GGDEF domain-containing protein	24	accessory
group_633	diguanylate phosphodiesterase	24	accessory
group_650	hypothetical protein	24	accessory

group_652	LysR family transcriptional regulator	24	accessory
group_655	diguanylate cyclase	24	accessory
group_658	hypothetical protein	24	accessory
group_659	diguanylate cyclase	24	accessory
group_674	LacI family transcriptional regulator	24	accessory
zipA	cell division protein ZipA	24	accessory
lptE	LPS-assembly lipoprotein LptE	24	accessory
group_73	MSHA pilin protein MshC	24	accessory
group_769	UPF0231 protein	24	accessory
group_786	twitching motility protein PilT	24	accessory
group_798	MSHA biogenesis protein MshF	24	accessory
group_890	AraC family transcriptional regulator	24	accessory
group_895	DNA-binding response regulator	24	accessory
group_91	hypothetical protein	24	accessory
group_914	tellurium resistance protein	24	accessory
group_93	methyl-accepting chemotaxis protein	24	accessory
group_955	UPF0213 protein	24	accessory
group_989	3'3'-cGAMP-specific phosphodiesterase 3	24	accessory
group_1178	cytochrome c biogenesis protein CcsA	23	accessory
group_1181	hypothetical protein	23	accessory
rpmD	50S ribosomal protein L30	23	accessory
group_1253	hypothetical protein	23	accessory
group_1336	peptidase C39	23	accessory
group_1344	MFS transporter	23	accessory
group_1351	response regulator	23	accessory
group_1352	transcriptional regulator	23	accessory
group_1435	iron(III) ABC transporter permease	23	accessory
group_146	cell envelope biogenesis protein AsmA	23	accessory
group_1527	Na+/H+ antiporter	23	accessory
group_1703	cytochrome c oxidase, cbb3-type subunit I	23	accessory
group_2324	aconitate hydratase	23	accessory
group_250	hypothetical protein	23	accessory
group_283	multidrug resistance protein	23	accessory
group_286	ferrochelatase	23	accessory
group_290	hypothetical protein	23	accessory
group_334	MSHA pilin protein MshD	23	accessory
group_4577	tRNA-Tyr	23	accessory
group_610	hypothetical protein	23	accessory
group_66	hypothetical protein	23	accessory

group_663	response regulator	23	accessory
group_707	protein-tyrosine-phosphatase	23	accessory
group_1002	glutaredoxin 2	22	accessory
fmt	methionyl-tRNA formyltransferase	22	accessory
group_1215	2',3'-cyclic-nucleotide 2'-phosphodiesterase	22	accessory
group_1221	membrane protein	22	accessory
group_1252	hemolysin	22	accessory
group_1309	paraquat-inducible protein B	22	accessory
fnr	fumarate and nitrate reduction regulatory protein	22	accessory
group_1367	hemolysin	22	accessory
group_1452	ligase	22	accessory
group_1454	tRNA-Thr	22	accessory
group_149	hypothetical protein	22	accessory
group_1518	phosphate ABC transporter substrate-binding protein	22	accessory
group_1539	aspartate aminotransferase family protein	22	accessory
group_1595	DNA-binding response regulator	22	accessory
group_1596	regulatory protein UhpC	22	accessory
group_1597	iron(III) ABC transporter substrate-binding protein	22	accessory
group_1598	iron(III) ABC transporter permease	22	accessory
fbpC	Fe(3+) ions import ATP-binding protein FbpC	22	accessory
group_1600	acetyl-CoA acetyltransferase	22	accessory
group_1606	GGDEF domain-containing protein	22	accessory
group_1659	LysR family transcriptional regulator	22	accessory
group_1660	alkene reductase	22	accessory
group_1687	sulfate transporter	22	accessory
group_170	hypothetical protein	22	accessory
group_314	UDP-N-acetyl-D-mannosamine transferase	22	accessory
group_315	hypothetical protein	22	accessory
group_341	IclR family transcriptional regulator	22	accessory
group_376	hypothetical protein	22	accessory
group_604	cell division protein	22	accessory
group_620	serine protease	22	accessory
group_750	LysR family transcriptional regulator	22	accessory
group_77	hypothetical protein	22	accessory
group_877	universal stress protein	22	accessory
group_930	PTS sugar transporter subunit IIA	22	accessory
group_947	sensor histidine kinase	22	accessory
group_1	hypothetical protein	21	accessory
group_120	2-succinylbenzoate-CoA ligase	21	accessory

group_122	hypothetical protein	21	accessory
group_1247	microcin C ABC transporter permease YejB	21	accessory
group_1248	peptide ABC transporter permease	21	accessory
group_1249	ABC transporter substrate-binding protein	21	accessory
group_1250	peptide ABC transporter ATP-binding protein	21	accessory
group_130	DTW domain-containing protein	21	accessory
ttcA	tRNA 2-thiocytidine biosynthesis protein TtcA	21	accessory
dnaG	DNA primase	21	accessory
group_1478	hypothetical protein	21	accessory
group_1555	antitoxin	21	accessory
group_1580	hypothetical protein	21	accessory
togA	ABC transporter ATP-binding protein	21	accessory
group_1582	hypothetical protein	21	accessory
group_1583	cyclodextrin-binding protein	21	accessory
group_1584	maltose ABC transporter permease	21	accessory
group_1585	sugar ABC transporter permease	21	accessory
malZ	cyclomaltodextrinase	21	accessory
group_1604	UDP-glucose 4-epimerase	21	accessory
group_1670	MBL fold metallo-hydrolase	21	accessory
group_182	hypothetical protein	21	accessory
group_2064	hybrid sensor histidine kinase/response regulator	21	accessory
group_238	hypothetical protein	21	accessory
group_245	ABC transporter ATP-binding protein	21	accessory
group_31	vibriobactin synthase D	21	accessory
syd	protein Syd	21	accessory
group_345	hypothetical protein	21	accessory
group_346	hypothetical protein	21	accessory
rtxA	multifunctional-autoprocessing repeats-in-toxin	21	accessory
group_401	glycosidase	21	accessory
group_4351	IS200/IS605 family transposase	21	accessory
group_4882	tRNA-Arg	21	accessory
group_515	hypothetical protein	21	accessory
group_627	membrane protein	21	accessory
group_896	hypothetical protein	21	accessory
group_906	plasmid stabilization protein	21	accessory
lpxD_2	hypothetical protein	21	accessory
group_940	hypothetical protein	21	accessory
group_941	hypothetical protein	21	accessory
group_980	O-acetylhomoserine aminocarboxypropyltransferase	21	accessory

group_1312	diguanylate phosphodiesterase	20	accessory
group_1313	response regulator	20	accessory
group_1342	hypothetical protein	20	accessory
group_1343	methyl-accepting chemotaxis protein	20	accessory
group_1432	hypothetical protein	20	accessory
group_1656	DUF805 domain-containing protein	20	accessory
group_385	N-acetyltransferase	20	accessory
group_447	hypothetical protein	20	accessory
group_511	hypothetical protein	20	accessory
group_533	TetR family transcriptional regulator	20	accessory
group_67	hypothetical protein	20	accessory
group_78	hypothetical protein	20	accessory
group_833	lipopolysaccharide heptosyltransferase II	20	accessory
group_1222	tRNA-Leu	19	accessory
group_1231	hypothetical protein	19	accessory
group_1558	antitoxin	19	accessory
phnW	2-aminoethylphosphonate--pyruvate transaminase	19	accessory
group_1590	chitinase	19	accessory
group_1662	maltose operon protein	19	accessory
group_174	aspartate aminotransferase family protein	19	accessory
group_2476	hypothetical protein	19	accessory
group_398	ABC transporter ATP-binding protein	19	accessory
group_41	integrase	19	accessory
group_423	hypothetical protein	19	accessory
group_4785	tRNA-His	19	accessory
group_5031	tRNA-Leu	19	accessory
group_651	hypothetical protein	19	accessory
group_80	hypothetical protein	19	accessory
group_82	ABC transporter permease	19	accessory
group_938	ABC transporter substrate-binding protein	19	accessory
group_1000	methyl-accepting chemotaxis protein	18	accessory
group_107	transposase	18	accessory
group_1198	hypothetical protein	18	accessory
group_1199	sugar ABC transporter substrate-binding protein	18	accessory
rbsA_2	ribose import ATP-binding protein RbsA	18	accessory
group_1201	ABC transporter permease	18	accessory
kefB	potassium transporter	18	accessory
group_1373	general secretion pathway protein GspH	18	accessory
group_1456	nucleotidyltransferase	18	accessory

group_1501	hypothetical protein	18	accessory
ulaA_1	PTS ascorbate transporter subunit IIC	18	accessory
group_1524	PTS ascorbate transporter subunit IIA	18	accessory
group_1525	transcriptional regulator	18	accessory
higA-1	antitoxin HigA-1	18	accessory
group_1552	DNA damage-inducible protein DinB	18	accessory
group_1564	hypothetical protein	18	accessory
group_1574	DNA-binding response regulator	18	accessory
group_1575	conjugal transfer protein TraF	18	accessory
group_1605	hypothetical protein	18	accessory
group_1609	GntR family transcriptional regulator	18	accessory
group_246	hypothetical protein	18	accessory
group_262	universal stress protein	18	accessory
group_310	electron transport complex subunit C	18	accessory
group_391	sensor histidine kinase	18	accessory
group_397	MFS transporter	18	accessory
group_426	hypothetical protein	18	accessory
group_427	cytochrome d ubiquinol oxidase subunit II	18	accessory
group_510	tRNA-Ala	18	accessory
group_516	beta-glucosidase	18	accessory
group_736	type IV pilin	18	accessory
group_821	hypothetical protein	18	accessory
group_874	hypothetical protein	18	accessory
group_876	PTS ascorbate transporter subunit IIB	18	accessory
group_886	GGDEF domain-containing protein	18	accessory
group_900	hypothetical protein	18	accessory
group_907	RelE protein	18	accessory
group_908	plasmid maintenance protein CcdB	18	accessory
kup	putative potassium transport system protein kup	18	accessory
group_916	pyruvate:ferredoxin (flavodoxin) oxidoreductase	18	accessory
tatA_1	Sec-independent protein translocase protein TatA	18	accessory
yqjA	membrane protein	18	accessory
group_970	hydrolase	18	accessory
group_1023	hypothetical protein	17	accessory
group_1314	MBL fold metallo-hydrolase	17	accessory
group_1370	sugar transporter	17	accessory
group_1410	transcriptional regulator	17	accessory
hcr	hybrid-cluster NAD(P)-dependent oxidoreductase	17	accessory
group_1534	copper-binding protein	17	accessory

group_1535	cation transporter	17	accessory
group_1536	hemolysin D	17	accessory
group_1537	copper transporter	17	accessory
group_1538	hypothetical protein	17	accessory
group_1546	antitoxin	17	accessory
group_1547	plasmid stabilization protein	17	accessory
group_1572	RelE toxin	17	accessory
group_25	hypothetical protein	17	accessory
nanM	hypothetical protein	17	accessory
group_253	hypothetical protein	17	accessory
group_322	toxin HipA	17	accessory
hcp	hydroxylamine reductase	17	accessory
group_368	hypothetical protein	17	accessory
group_384	polyketide cyclase	17	accessory
group_387	hypothetical protein	17	accessory
group_396	hypothetical protein	17	accessory
group_599	hypothetical protein	17	accessory
group_673	metal-dependent phosphohydrolase	17	accessory
group_911	hypothetical protein	17	accessory
group_106	hypothetical protein	16	accessory
btuB	vitamin B12 transporter BtuB	16	accessory
group_1202	hypothetical protein	16	accessory
group_147	hypothetical protein	16	accessory
group_1532	hypothetical protein	16	accessory
group_1533	hypothetical protein	16	accessory
group_1549	hypothetical protein	16	accessory
group_1553	hypothetical protein	16	accessory
group_159	hexapeptide transferase	16	accessory
group_316	hypothetical protein	16	accessory
higB-1	toxin HigB-1	16	accessory
hutA	ligand-gated channel	16	accessory
group_892	hypothetical protein	16	accessory
group_1052	methyl-accepting chemotaxis protein	15	accessory
group_1053	hypothetical protein	15	accessory
group_1054	hypothetical protein	15	accessory
group_1220	outer membrane protein OmpK	15	accessory
group_1368	polysaccharide biosynthesis protein	15	accessory
group_1369	capsular polysaccharide biosynthesis protein CapK	15	accessory
group_1464	lipid carrier--UDP-N-acetylgalactosaminyltransferase	15	accessory

group_1465	mannosyltransferase	15	accessory
group_1611	hypothetical protein	15	accessory
group_1612	hypothetical protein	15	accessory
group_1613	hypothetical protein	15	accessory
group_1658	hypothetical protein	15	accessory
group_1726	antitoxin	15	accessory
group_1773	hypothetical protein	15	accessory
group_1798	3-oxoacyl-ACP reductase	15	accessory
group_183	hypothetical protein	15	accessory
wbfB	WbfB protein	15	accessory
group_386	hypothetical protein	15	accessory
group_452	hypothetical protein	15	accessory
group_454	fucose 4-O-acetylase	15	accessory
group_456	biotin synthesis protein BioC	15	accessory
group_457	ATPase	15	accessory
group_476	hypothetical protein	15	accessory
tkt1	transketolase 1	15	accessory
group_685	hypothetical protein	15	accessory
group_726	glycosyl transferase family 1	15	accessory
group_727	phosphotyrosine protein phosphatase	15	accessory
group_74	hypothetical protein	15	accessory
group_75	transcriptional regulator	15	accessory
wbfD	hypothetical protein	15	accessory
group_829	UDP-glucose 4-epimerase	15	accessory
bioF	8-amino-7-oxononanoate synthase	15	accessory
yhhY	acetyltransferase	14	accessory
group_1219	hypothetical protein	14	accessory
group_1254	hypothetical protein	14	accessory
group_132	hypothetical protein	14	accessory
group_1371	hypothetical protein	14	accessory
group_1372	pilus assembly protein PilW	14	accessory
group_1407	transcriptional regulator	14	accessory
group_1424	conjugal transfer relaxase TraI	14	accessory
group_1426	transcriptional regulator	14	accessory
group_1450	MSHA biogenesis protein MshO	14	accessory
group_1479	hypothetical protein	14	accessory
group_1480	hexapeptide transferase	14	accessory
group_1481	acyltransferase	14	accessory
group_1556	antitoxin	14	accessory

group_1557	hypothetical protein	14	accessory
group_1587	transcriptional regulator	14	accessory
group_1610	hypothetical protein	14	accessory
group_171	hypothetical protein	14	accessory
group_1838	5-formyltetrahydrofolate cyclo-ligase	14	accessory
group_2374	hypothetical protein	14	accessory
group_634	LysR family transcriptional regulator	14	accessory
group_741	UPF0758 protein	14	accessory
group_742	hypothetical protein	14	accessory
group_743	hypothetical protein	14	accessory
group_744	GTPase	14	accessory
group_745	transcriptional regulator	14	accessory
group_753	hypothetical protein	14	accessory
group_754	P-type conjugative transfer protein TrbL	14	accessory
group_755	plasmid replication protein	14	accessory
group_795	hypothetical protein	14	accessory
group_796	hypothetical protein	14	accessory
kdkA	3-deoxy-D-manno-octulosonic acid kinase	14	accessory
group_883	chromosome segregation ATPase	14	accessory
group_1194	tRNA-Val	13	accessory
group_1315	hypothetical protein	13	accessory
group_1430	phage-related integrase	13	accessory
group_1567	hypothetical protein	13	accessory
group_1568	phosphotransferase	13	accessory
group_1844	hypothetical protein	13	accessory
group_196	hypothetical protein	13	accessory
group_3944	long-chain fatty acid transporter	13	accessory
group_737	prepilin-type N-terminal cleavage/methylation domain-containing protein	13	accessory
group_775	hypothetical protein	13	accessory
group_79	hypothetical protein	13	accessory
group_901	hypothetical protein	13	accessory
group_1044	hypothetical protein	12	accessory
group_1067	hypothetical protein	12	accessory
wzc	tyrosine protein kinase	12	accessory
group_1235	starvation lipoprotein Slp	12	accessory
group_1366	porin	12	accessory
group_1463	hypothetical protein	12	accessory
group_1487	UPF0758 protein	12	accessory
group_1512	hypothetical protein	12	accessory

group_1526	hypothetical protein	12	accessory
group_1542	DNA-binding protein	12	accessory
group_1548	hypothetical protein	12	accessory
group_160	hypothetical protein	12	accessory
group_161	hypothetical protein	12	accessory
group_1620	hypothetical protein	12	accessory
lamB	maltoporin	12	accessory
group_1708	hypothetical protein	12	accessory
group_1783	prepilin-type N-terminal cleavage/methylation domain-containing protein	12	accessory
group_193	methyl-accepting chemotaxis protein	12	accessory
int_2	integrase	12	accessory
group_344	hypothetical protein	12	accessory
yagK_1	hypothetical protein	12	accessory
group_448	transcriptional regulator	12	accessory
group_778	hypothetical protein	12	accessory
group_779	transposase	12	accessory
etp	phosphotyrosine protein phosphatase	12	accessory
gfcE	polysaccharide export protein Wza	12	accessory
wbfC	hypothetical protein	12	accessory
rffG	dTDP-glucose 4,6-dehydratase	12	accessory
group_848	resolvase	12	accessory
group_905	hypothetical protein	12	accessory
group_1048	pilus assembly protein PilW	11	accessory
group_1317	hypothetical protein	11	accessory
group_1411	dihydropteroate synthase	11	accessory
group_1416	IS91 family transposase	11	accessory
group_1445	IS110 family transposase	11	accessory
group_1457	hypothetical protein	11	accessory
group_1505	hypothetical protein	11	accessory
hsdM	restriction endonuclease subunit M	11	accessory
group_1508	hypothetical protein	11	accessory
group_1509	hypothetical protein	11	accessory
group_1510	DNA-binding protein	11	accessory
group_1511	hypothetical protein	11	accessory
group_1544	Qnr family quinolone resistance pentapeptide repeat protein	11	accessory
group_1559	hypothetical protein	11	accessory
group_1594	hypothetical protein	11	accessory
group_1616	peptidase M23	11	accessory
group_1621	hypothetical protein	11	accessory

group_1622	hypothetical protein	11	accessory
group_1623	hypothetical protein	11	accessory
group_1624	hypothetical protein	11	accessory
group_1625	hypothetical protein	11	accessory
group_1626	hypothetical protein	11	accessory
group_1627	hypothetical protein	11	accessory
group_1628	hypothetical protein	11	accessory
group_1629	hypothetical protein	11	accessory
group_1630	major capsid protein	11	accessory
group_1631	hypothetical protein	11	accessory
group_1633	hypothetical protein	11	accessory
group_1635	hypothetical protein	11	accessory
group_1636	holin	11	accessory
group_1645	portal protein	11	accessory
group_1646	hypothetical protein	11	accessory
group_1647	hypothetical protein	11	accessory
group_1649	hypothetical protein	11	accessory
group_169	hypothetical protein	11	accessory
group_1690	hypothetical protein	11	accessory
group_184	hypothetical protein	11	accessory
group_19	GCN5 family acetyltransferase	11	accessory
group_199	hypothetical protein	11	accessory
group_2030	MSHA biogenesis protein MshO	11	accessory
group_2158	LD-carboxypeptidase	11	accessory
group_2160	hypothetical protein	11	accessory
waaL	O-antigen ligase	11	accessory
group_3860	terminase	11	accessory
group_458	hypothetical protein	11	accessory
group_534	5-formyltetrahydrofolate cyclo-ligase	11	accessory
group_739	integrase	11	accessory
group_777	transposase	11	accessory
group_823	hypothetical protein	11	accessory
rffH	glucose-1-phosphate thymidylyltransferase	11	accessory
group_832	Lex2B	11	accessory
hsdR	type I restriction endonuclease	11	accessory
group_850	hypothetical protein	11	accessory
group_891	hypothetical protein	11	accessory
group_902	stress protein	11	accessory
group_971	hypothetical protein	11	accessory

group_972	hypothetical protein	11	accessory
group_973	hypothetical protein	11	accessory
group_974	hypothetical protein	11	accessory
group_975	hypothetical protein	11	accessory
group_976	hypothetical protein	11	accessory
group_977	terminase	11	accessory
group_978	hypothetical protein	11	accessory
group_1026	hypothetical protein	10	accessory
group_1051	lipoprotein NlpC	10	accessory
group_1184	hypothetical protein	10	accessory
group_1185	hypothetical protein	10	accessory
group_1187	hypothetical protein	10	accessory
group_1189	hypothetical protein	10	accessory
group_1190	TatD family hydrolase	10	accessory
group_1191	hypothetical protein	10	accessory
group_1192	hypothetical protein	10	accessory
group_1196	ATPase	10	accessory
group_1203	hypothetical protein	10	accessory
group_1204	hypothetical protein	10	accessory
group_1213	prepilin-type N-terminal cleavage/methylation domain-containing protein	10	accessory
group_1216	hypothetical protein	10	accessory
group_1224	6-phosphogluconolactonase	10	accessory
group_1225	ketohydroxyglutarate aldolase	10	accessory
group_1226	sodium:proline symporter	10	accessory
group_1227	reactive intermediate/imine deaminase	10	accessory
dan	D-aminoacylase	10	accessory
group_1229	aldolase	10	accessory
group_1230	transcriptional regulator	10	accessory
group_1236	terminase	10	accessory
group_1237	hypothetical protein	10	accessory
group_1238	hypothetical protein	10	accessory
group_1239	hypothetical protein	10	accessory
group_1240	peptidase	10	accessory
group_1241	hypothetical protein	10	accessory
group_1242	hypothetical protein	10	accessory
group_1243	hypothetical protein	10	accessory
group_1244	hypothetical protein	10	accessory
group_1245	hypothetical protein	10	accessory
int_1	integrase	10	accessory

group_1255	porin	10	accessory
group_1256	UPF0319 protein	10	accessory
group_1259	hypothetical protein	10	accessory
group_1262	hypothetical protein	10	accessory
group_1263	hypothetical protein	10	accessory
tdh2	thermostable direct hemolysin 2	10	accessory
group_1268	hypothetical protein	10	accessory
group_1271	hypothetical protein	10	accessory
group_1272	hypothetical protein	10	accessory
group_1273	hypothetical protein	10	accessory
group_1274	hypothetical protein	10	accessory
group_1275	type III secretion protein	10	accessory
group_1276	hypothetical protein	10	accessory
group_1277	hypothetical protein	10	accessory
group_1278	hypothetical protein	10	accessory
group_1279	hypothetical protein	10	accessory
group_1280	hypothetical protein	10	accessory
group_1281	hypothetical protein	10	accessory
group_1282	hypothetical protein	10	accessory
group_1283	dimethyladenosine transferase	10	accessory
group_1284	hypothetical protein	10	accessory
group_1285	hypothetical protein	10	accessory
group_1286	type III secretion protein	10	accessory
group_1287	type III secretion protein	10	accessory
group_1288	outer membrane protein	10	accessory
group_1289	hypothetical protein	10	accessory
group_1290	hypothetical protein	10	accessory
group_1291	hypothetical protein	10	accessory
group_1292	type III secretion protein	10	accessory
group_1293	type III secretion system protein	10	accessory
group_1294	hypothetical protein	10	accessory
group_1295	hypothetical protein	10	accessory
group_1296	secretin	10	accessory
group_1297	type III secretion system ATPase	10	accessory
group_1298	hypothetical protein	10	accessory
group_1300	type III secretion protein	10	accessory
group_1307	accessory colonization factor AcfA	10	accessory
group_1308	hypothetical protein	10	accessory
group_1316	methyl-accepting chemotaxis protein	10	accessory

group_1318	hypothetical protein	10	accessory
group_1319	hypothetical protein	10	accessory
group_1320	hypothetical protein	10	accessory
group_1321	hypothetical protein	10	accessory
group_1322	hypothetical protein	10	accessory
group_1324	toxin	10	accessory
group_1325	hypothetical protein	10	accessory
group_1326	hypothetical protein	10	accessory
group_1327	hypothetical protein	10	accessory
group_1328	hypothetical protein	10	accessory
group_1329	hypothetical protein	10	accessory
group_1330	hypothetical protein	10	accessory
group_1331	hypothetical protein	10	accessory
group_1332	hypothetical protein	10	accessory
group_1333	antirepressor	10	accessory
group_1361	biotin synthesis protein BioC	10	accessory
group_1362	8-amino-7-oxononanoate synthase	10	accessory
group_1374	integrase	10	accessory
group_1375	hypothetical protein	10	accessory
group_1376	hypothetical protein	10	accessory
group_1377	hypothetical protein	10	accessory
fliP_2	flagellar biosynthetic protein FliP	10	accessory
group_1379	hypothetical protein	10	accessory
group_1380	hypothetical protein	10	accessory
group_1381	hypothetical protein	10	accessory
flgE	flagellar hook protein FlgE	10	accessory
group_1383	flagellar hook capping protein	10	accessory
group_1384	hypothetical protein	10	accessory
group_1385	hypothetical protein	10	accessory
group_1386	hypothetical protein	10	accessory
group_1387	hypothetical protein	10	accessory
group_1388	hypothetical protein	10	accessory
group_1389	hypothetical protein	10	accessory
group_1390	hypothetical protein	10	accessory
group_1391	hypothetical protein	10	accessory
group_1392	hypothetical protein	10	accessory
flgI_1	flagellar P-ring protein	10	accessory
group_1394	hypothetical protein	10	accessory
group_1395	hypothetical protein	10	accessory

flgG	flagellar basal body rod protein FlgG	10	accessory
group_1397	flagellar basal-body rod protein FlgF	10	accessory
group_1398	hypothetical protein	10	accessory
group_1399	hypothetical protein	10	accessory
group_1400	flagellar export apparatus protein FliQ	10	accessory
group_1401	hypothetical protein	10	accessory
group_1402	hypothetical protein	10	accessory
group_1403	hypothetical protein	10	accessory
group_1404	hypothetical protein	10	accessory
group_1405	hypothetical protein	10	accessory
group_1406	hypothetical protein	10	accessory
yagK_2	hypothetical protein	10	accessory
group_1412	hypothetical protein	10	accessory
group_1413	hypothetical protein	10	accessory
group_1414	chloramphenicol efflux MFS transporter	10	accessory
group_1415	hypothetical protein	10	accessory
group_1417	hypothetical protein	10	accessory
group_1418	hypothetical protein	10	accessory
group_1419	hypothetical protein	10	accessory
group_1420	hypothetical protein	10	accessory
group_1421	hypothetical protein	10	accessory
group_1422	hypothetical protein	10	accessory
group_1423	conjugal transfer protein TrbJ	10	accessory
group_1425	hypothetical protein	10	accessory
group_1427	LuxR family transcriptional regulator	10	accessory
ompU	outer membrane protein U	10	accessory
group_1441	site-specific DNA-methyltransferase	10	accessory
group_1442	hypothetical protein	10	accessory
group_1443	hypothetical protein	10	accessory
group_1444	hypothetical protein	10	accessory
group_1449	hypothetical protein	10	accessory
group_1451	MSHA pilin protein MshA	10	accessory
tuaG	putative teichuronic acid biosynthesis glycosyltransferase TuaG	10	accessory
group_1459	UDP-glucose 6-dehydrogenase	10	accessory
group_1468	galactosyl transferase	10	accessory
group_1475	transport permease protein	10	accessory
group_1476	spore coat protein	10	accessory
rfbC-2	dTDP-4-dehydrorhamnose 3,5-epimerase	10	accessory
group_1482	phage-related integrase	10	accessory

group_1483	hypothetical protein	10	accessory
group_1484	hypothetical protein	10	accessory
group_1485	hypothetical protein	10	accessory
group_1486	hypothetical protein	10	accessory
group_1488	hypothetical protein	10	accessory
group_1489	hypothetical protein	10	accessory
group_1490	twin-arginine translocation pathway signal	10	accessory
group_1491	sodium-independent anion transporter	10	accessory
group_1492	TIGR01244 family protein	10	accessory
group_1493	hypothetical protein	10	accessory
group_1494	peroxiredoxin	10	accessory
group_1495	ribonuclease H	10	accessory
group_1496	transcriptional regulator	10	accessory
group_1497	hypothetical protein	10	accessory
group_1498	hypothetical protein	10	accessory
group_1499	hypothetical protein	10	accessory
group_1502	hypothetical protein	10	accessory
group_1503	hypothetical protein	10	accessory
group_1504	hypothetical protein	10	accessory
group_1507	hypothetical protein	10	accessory
group_1513	hypothetical protein	10	accessory
group_1514	hypothetical protein	10	accessory
group_1515	hypothetical protein	10	accessory
group_1516	fucose 4-O-acetylase	10	accessory
group_1520	hypothetical protein	10	accessory
group_1521	hypothetical protein	10	accessory
group_1522	hypothetical protein	10	accessory
group_1541	hypothetical protein	10	accessory
group_1543	hypothetical protein	10	accessory
group_1550	hypothetical protein	10	accessory
group_1561	oxidoreductase	10	accessory
group_1563	hypothetical protein	10	accessory
group_1565	glyoxalase	10	accessory
group_1566	hypothetical protein	10	accessory
group_1570	protein phosphatase	10	accessory
group_1571	hypothetical protein	10	accessory
group_1577	hypothetical protein	10	accessory
group_1588	hypothetical protein	10	accessory
group_1591	hypothetical protein	10	accessory

group_1592	hypothetical protein	10	accessory
group_1603	hypothetical protein	10	accessory
group_1614	hypothetical protein	10	accessory
group_1617	hypothetical protein	10	accessory
group_1618	hypothetical protein	10	accessory
group_1619	hypothetical protein	10	accessory
group_1632	hypothetical protein	10	accessory
group_1634	hypothetical protein	10	accessory
arsC2	protein-tyrosine-phosphatase	10	accessory
group_1638	arsenical-resistance protein	10	accessory
group_1639	hypothetical protein	10	accessory
group_1640	hypothetical protein	10	accessory
group_1641	hypothetical protein	10	accessory
cspV_1	cold shock protein CspV	10	accessory
cro	transcriptional regulator	10	accessory
group_1650	hypothetical protein	10	accessory
group_1651	cytosine-specific methyltransferase	10	accessory
group_1652	cytosine-specific methyltransferase	10	accessory
group_1653	hypothetical protein	10	accessory
group_1654	hypothetical protein	10	accessory
group_1664	sensor domain-containing diguanylate cyclase	10	accessory
group_1688	hypothetical protein	10	accessory
group_1691	beta-N-acetylhexosaminidase	10	accessory
group_194	hypothetical protein	10	accessory
group_197	hypothetical protein	10	accessory
group_198	hypothetical protein	10	accessory
group_224	hypothetical protein	10	accessory
group_254	hypothetical protein	10	accessory
group_255	hypothetical protein	10	accessory
group_321	flagellar biosynthesis protein FlhA	10	accessory
group_351	hypothetical protein	10	accessory
group_460	phosphotyrosine protein phosphatase	10	accessory
group_475	hypothetical protein	10	accessory
group_508	hypothetical protein	10	accessory
group_545	hypothetical protein	10	accessory
kdgK	2-dehydro-3-deoxygluconokinase	10	accessory
group_592	hypothetical protein	10	accessory
group_740	hypothetical protein	10	accessory
group_752	aminoglycoside O-phosphotransferase APH(6)-Id	10	accessory

group_776	hypothetical protein	10	accessory
group_928	hypothetical protein	10	accessory
group_979	peptidase S24	10	accessory
group_1036	hypothetical protein	9	accessory
group_1068	hypothetical protein	9	accessory
group_1257	hypothetical protein	9	accessory
group_1261	hypothetical protein	9	accessory
group_1264	hypothetical protein	9	accessory
group_1266	hypothetical protein	9	accessory
peb3	accessory colonization factor AcfC	9	accessory
group_1303	transcriptional regulator	9	accessory
group_1304	hypothetical protein	9	accessory
group_1305	hypothetical protein	9	accessory
group_1306	hypothetical protein	9	accessory
group_1323	hypothetical protein	9	accessory
group_1466	hypothetical protein	9	accessory
group_1472	hypothetical protein	9	accessory
group_1474	polysaccharide/polyol phosphate ABC transporter ATP-binding protein	9	accessory
higA-2	antitoxin HigA-2	9	accessory
group_1576	hypothetical protein	9	accessory
group_1615	hypothetical protein	9	accessory
nhaA_1	Na(+)/H(+) antiporter NhaA	9	accessory
group_1643	hypothetical protein	9	accessory
group_1693	hypothetical protein	9	accessory
group_1694	hypothetical protein	9	accessory
group_1695	hypothetical protein	9	accessory
group_1696	ATP-binding protein	9	accessory
group_1697	WYL domain-containing protein	9	accessory
group_1698	hypothetical protein	9	accessory
group_1699	hypothetical protein	9	accessory
group_1700	hypothetical protein	9	accessory
group_1709	chitinase	9	accessory
group_1731	acetyltransferase	9	accessory
group_1734	TonB-dependent receptor	9	accessory
group_1779	hypothetical protein	9	accessory
group_1915	vitamin B12 transporter BtuB	9	accessory
group_2033	starvation lipoprotein Slp	9	accessory
rpmJ2	50S ribosomal protein L36 2	9	accessory
group_2159	hypothetical protein	9	accessory

group_285	alkaline phosphatase	9	accessory
group_382	N-acetyltransferase	9	accessory
group_461	hypothetical protein	9	accessory
group_824	glycosyl transferase	9	accessory
group_855	hypothetical protein	9	accessory
group_904	hydrolase	9	accessory
group_929	hypothetical protein	9	accessory
group_1042	transcriptional regulator	8	accessory
group_1043	isochorismatase	8	accessory
group_1064	hypothetical protein	8	accessory
group_1073	hypothetical protein	8	accessory
group_1075	hypothetical protein	8	accessory
group_1084	hypothetical protein	8	accessory
group_1085	hypothetical protein	8	accessory
group_1188	hypothetical protein	8	accessory
group_1205	hypothetical protein	8	accessory
group_1267	hypothetical protein	8	accessory
group_1270	hypothetical protein	8	accessory
group_1299	hypothetical protein	8	accessory
group_1301	hypothetical protein	8	accessory
group_1462	hypothetical protein	8	accessory
group_1471	carbamoyl phosphate synthase	8	accessory
group_1569	hypothetical protein	8	accessory
group_1941	hypothetical protein	8	accessory
group_1960	phosphoglycerate mutase	8	accessory
group_2034	hypothetical protein	8	accessory
group_2035	hypothetical protein	8	accessory
group_2061	glycoporin	8	accessory
group_213	integrase	8	accessory
group_2140	antitoxin	8	accessory
group_2141	plasmid stabilization protein ParE	8	accessory
group_2161	plasmid stabilization protein	8	accessory
group_2173	hypothetical protein	8	accessory
group_45	N-acetyltransferase	8	accessory
group_463	DUF1289 domain-containing protein	8	accessory
group_473	hypothetical protein	8	accessory
group_606	hypothetical protein	8	accessory
group_607	hypothetical protein	8	accessory
group_608	hypothetical protein	8	accessory

tufB_1	hypothetical protein	8	accessory
group_1029	mannose-6-phosphate isomerase	7	accessory
group_1030	PTS fructose transporter subunit IIA	7	accessory
group_1031	PTS fructose transporter subunit IIA	7	accessory
bdh	NADH-dependent alcohol dehydrogenase	7	accessory
group_1033	cupin	7	accessory
group_1035	GDXG family lipase	7	accessory
group_1040	hypothetical protein	7	accessory
group_1058	hypothetical protein	7	accessory
group_1059	lipoprotein ABC transporter ATP-binding protein LolD	7	accessory
group_1060	hypothetical protein	7	accessory
blc	outer membrane lipoprotein Blc	7	accessory
group_1079	N-acetylglucosamine-6-phosphate deacetylase	7	accessory
group_1177	integrase	7	accessory
group_1409	hypothetical protein	7	accessory
group_1428	hypothetical protein	7	accessory
group_1461	hypothetical protein	7	accessory
group_1467	hypothetical protein	7	accessory
group_1469	hypothetical protein	7	accessory
group_1470	metallophosphoesterase	7	accessory
group_1473	hypothetical protein	7	accessory
group_1607	hypothetical protein	7	accessory
group_1713	PTS fructose transporter subunit IIC	7	accessory
group_1714	transcriptional regulator	7	accessory
group_1715	PTS fructose transporter subunit IIA	7	accessory
group_1716	PTS fructose transporter subunit IIB	7	accessory
group_1717	PTS fructose transporter subunit IIC	7	accessory
group_1718	transcriptional regulator	7	accessory
group_1722	cytochrome c biogenesis protein	7	accessory
group_1727	plasmid stabilization protein	7	accessory
group_1729	hypothetical protein	7	accessory
group_1730	transcriptional regulator	7	accessory
group_1733	conjugal transfer protein TraF	7	accessory
group_1743	ferredoxin-type protein NapF	7	accessory
group_1752	membrane protein	7	accessory
group_1753	hypothetical protein	7	accessory
group_1757	methyl-accepting chemotaxis protein	7	accessory
group_1764	hypothetical protein	7	accessory
group_1765	RNA polymerase sigma factor	7	accessory

group_1766	hypothetical protein	7	accessory
group_1767	hypothetical protein	7	accessory
group_1768	copper-binding protein	7	accessory
group_1775	MarR family transcriptional regulator	7	accessory
group_1778	methyl-accepting chemotaxis protein	7	accessory
group_1782	general secretion pathway protein GspH	7	accessory
group_1849	putative response regulatory protein	7	accessory
group_1850	ATPase	7	accessory
group_1851	hypothetical protein	7	accessory
group_1852	outer membrane lipoprotein-sorting protein	7	accessory
group_1865	methyl-accepting chemotaxis protein	7	accessory
group_1867	hypothetical protein	7	accessory
group_1869	response regulator	7	accessory
group_1871	hypothetical protein	7	accessory
cheB3	chemotaxis response regulator protein-glutamate methylesterase of group 3 operon	7	accessory
group_1873	chemotaxis protein W	7	accessory
group_1874	methyl-accepting chemotaxis protein	7	accessory
group_1945	hypothetical protein	7	accessory
group_195	hypothetical protein	7	accessory
group_1950	hypothetical protein	7	accessory
group_1959	hypothetical protein	7	accessory
group_1964	hypothetical protein	7	accessory
group_1990	hypothetical protein	7	accessory
macA	hemolysin secretion protein D	7	accessory
macB	macrolide export ATP-binding/permease protein MacB	7	accessory
macC	RND transporter	7	accessory
group_2029	hypothetical protein	7	accessory
group_2071	transcriptional regulator	7	accessory
group_2072	N-acetylneuraminate lyase	7	accessory
siaM	sialic acid TRAP transporter large permease protein SiaM	7	accessory
siaQ	sialic acid TRAP transporter small permease protein SiaQ	7	accessory
siaP	sialic acid-binding periplasmic protein SiaP	7	accessory
nanE	putative N-acetylmannosamine-6-phosphate 2-epimerase	7	accessory
nanK	N-acetylmannosamine kinase	7	accessory
nanH	sialidase	7	accessory
group_2114	hypothetical protein	7	accessory
group_2127	type IV pilin	7	accessory
group_2128	hypothetical protein	7	accessory
group_2162	hypothetical protein	7	accessory

group_2163	hypothetical protein	7	accessory
group_2186	cold-shock protein	7	accessory
group_280	hypothetical protein	7	accessory
group_324	hypothetical protein	7	accessory
group_453	hypothetical protein	7	accessory
group_469	UPF0319 protein	7	accessory
group_471	3-deoxy-D-manno-octulosonic acid kinase	7	accessory
group_483	cell filamentation protein Fic	7	accessory
group_97	nucleoside-diphosphate sugar epimerase	7	accessory
group_1038	hypothetical protein	6	accessory
group_1082	hypothetical protein	6	accessory
group_1083	integrase	6	accessory
group_1133	hypothetical protein	6	accessory
group_1156	maltose operon protein	6	accessory
group_1258	hypothetical protein	6	accessory
group_1260	hypothetical protein	6	accessory
group_1302	hypothetical protein	6	accessory
group_1429	hypothetical protein	6	accessory
group_1460	hypothetical protein	6	accessory
group_1578	hypothetical protein	6	accessory
group_1608	hypothetical protein	6	accessory
group_1866	response regulator	6	accessory
group_1868	chemotaxis protein CheA	6	accessory
cheR2	chemotaxis protein methyltransferase 2	6	accessory
group_1939	N-acetyltransferase	6	accessory
group_2036	hypothetical protein	6	accessory
group_2037	hypothetical protein	6	accessory
group_2038	MFS transporter	6	accessory
group_2039	hypothetical protein	6	accessory
group_2081	porin	6	accessory
group_2086	hypothetical protein	6	accessory
group_2120	hypothetical protein	6	accessory
group_2147	hypothetical protein	6	accessory
group_2149	hypothetical protein	6	accessory
group_2150	hypothetical protein	6	accessory
group_2174	hypothetical protein	6	accessory
rnr_1	ribonuclease R	6	accessory
group_2270	hypothetical protein	6	accessory
group_2918	amidohydrolase	6	accessory

group_3052	hypothetical protein	6	accessory
group_3053	O-glycosyl hydrolase family 13	6	accessory
treC	glucan 1,6-alpha-glucosidase	6	accessory
group_343	O-antigen ligase family protein	6	accessory
group_451	hypothetical protein	6	accessory
group_466	2-aminoethylphosphonate--pyruvate transaminase	6	accessory
group_472	hypothetical protein	6	accessory
group_474	type IV secretion protein Rhs	6	accessory
group_485	N-acetyltransferase	6	accessory
abgR	LysR family transcriptional regulator	6	accessory
group_624	hypothetical protein	6	accessory
group_854	hypothetical protein	6	accessory
higB-2	toxin HigB-2	6	accessory
group_910	hypothetical protein	6	accessory
group_96	hypothetical protein	6	accessory
group_1041	hypothetical protein	5	accessory
group_1046	sodium:proton antiporter	5	accessory
group_1063	hypothetical protein	5	accessory
group_1080	hypothetical protein	5	accessory
group_1091	outer membrane protein U	5	accessory
group_1122	serine acetyltransferase	5	accessory
rulB	DNA polymerase V subunit UmuC	5	accessory
umuD	DNA polymerase V	5	accessory
group_1143	hypothetical protein	5	accessory
group_1175	hypothetical protein	5	accessory
group_1186	hypothetical protein	5	accessory
group_1759	hypothetical protein	5	accessory
group_1834	lipopolysaccharide heptosyltransferase II	5	accessory
group_1835	LPS biosynthesis protein	5	accessory
group_1935	membrane protein	5	accessory
group_2003	DUF805 domain-containing protein	5	accessory
group_2028	hypothetical protein	5	accessory
group_2068	hypothetical protein	5	accessory
group_2082	hypothetical protein	5	accessory
group_2083	hypothetical protein	5	accessory
group_2148	WYL domain-containing protein	5	accessory
group_2151	hypothetical protein	5	accessory
group_2152	hypothetical protein	5	accessory
group_2153	hypothetical protein	5	accessory

group_2178	hypothetical protein	5	accessory
group_2187	MarR family transcriptional regulator	5	accessory
group_2296	hypothetical protein	5	accessory
group_2375	serine acetyltransferase	5	accessory
group_2376	hypothetical protein	5	accessory
group_2377	sugar transporter	5	accessory
group_2378	glycosyl transferase family 1	5	accessory
group_2386	aerotaxis receptor Aer	5	accessory
group_2502	hypothetical protein	5	accessory
group_3209	transcriptional regulator	5	accessory
group_3381	N-acetyltransferase	5	accessory
group_3524	hypothetical protein	5	accessory
group_470	hypothetical protein	5	accessory
group_487	ISAs1 family transposase	5	accessory
group_1045	outer membrane protein U	4	accessory
group_1050	OriT-binding protein, TraJ	4	accessory
wzt	ABC transporter	4	accessory
group_1057	3-deoxy-D-manno-octulosonic acid kinase	4	accessory
group_1061	hypothetical protein	4	accessory
group_1072	hypothetical protein	4	accessory
group_1088	hypothetical protein	4	accessory
group_1090	hypothetical protein	4	accessory
group_1111	sensor histidine kinase	4	accessory
group_1112	Ktr system potassium transporter B	4	accessory
group_1113	hypothetical protein	4	accessory
group_1127	DNA primase	4	accessory
group_1128	hypothetical protein	4	accessory
group_1131	hypothetical protein	4	accessory
group_1137	hypothetical protein	4	accessory
group_1139	hypothetical protein	4	accessory
group_1140	hypothetical protein	4	accessory
group_1142	hypothetical protein	4	accessory
group_1148	RNA-binding protein	4	accessory
group_1157	hypothetical protein	4	accessory
group_1179	hypothetical protein	4	accessory
yncI	putative transposase YncI	4	accessory
group_1554	hypothetical protein	4	accessory
group_1562	hypothetical protein	4	accessory
group_1689	hypothetical protein	4	accessory

group_1705	starvation lipoprotein Slp	4	accessory
group_1706	peptidase	4	accessory
group_1707	2-succinylbenzoate-CoA ligase	4	accessory
group_1710	hypothetical protein	4	accessory
group_1711	porin	4	accessory
group_1712	hypothetical protein	4	accessory
group_1719	ABC transporter substrate-binding protein	4	accessory
group_1720	hypothetical protein	4	accessory
group_1721	hypothetical protein	4	accessory
group_1723	nucleotide pyrophosphohydrolase	4	accessory
group_1724	hypothetical protein	4	accessory
group_1725	GCN5 family acetyltransferase	4	accessory
group_1728	glutathione S-transferase	4	accessory
group_1732	hypothetical protein	4	accessory
group_1735	hypothetical protein	4	accessory
group_1736	hypothetical protein	4	accessory
group_1737	hypothetical protein	4	accessory
group_1738	hypothetical protein	4	accessory
group_1739	sodium:solute symporter	4	accessory
group_1740	MFS transporter	4	accessory
group_1741	polyketide cyclase	4	accessory
group_1742	LacI family transcriptional regulator	4	accessory
group_1744	cytochrome b	4	accessory
group_1745	cytochrome b	4	accessory
group_1746	ATPase	4	accessory
group_1747	hypothetical protein	4	accessory
group_1748	hypothetical protein	4	accessory
group_1749	glyoxalase family protein	4	accessory
group_1750	LysR family transcriptional regulator	4	accessory
group_1751	UDP-glucose 4-epimerase	4	accessory
group_1754	hypothetical protein	4	accessory
group_1755	MFP transporter	4	accessory
group_1756	GTPase	4	accessory
group_1758	hypothetical protein	4	accessory
group_1760	hypothetical protein	4	accessory
group_1761	hypothetical protein	4	accessory
group_1762	hypothetical protein	4	accessory
group_1763	hypothetical protein	4	accessory
group_1769	hypothetical protein	4	accessory

group_1770	hypothetical protein	4	accessory
group_1771	hypothetical protein	4	accessory
group_1772	hypothetical protein	4	accessory
group_1776	hypothetical protein	4	accessory
group_1777	maltoporin	4	accessory
group_1780	long-chain fatty acid transporter	4	accessory
group_1781	hypothetical protein	4	accessory
group_1784	alkyl/aryl-sulfatase	4	accessory
group_1785	LysR family transcriptional regulator	4	accessory
group_1786	vibriobactin synthase D	4	accessory
group_1787	hypothetical protein	4	accessory
group_1788	hypothetical protein	4	accessory
group_1789	hypothetical protein	4	accessory
group_1790	hypothetical protein	4	accessory
group_1791	hypothetical protein	4	accessory
group_1792	conjugal transfer protein TrbJ	4	accessory
group_1793	LuxR family transcriptional regulator	4	accessory
group_1794	hypothetical protein	4	accessory
group_1796	hypothetical protein	4	accessory
group_1797	hypothetical protein	4	accessory
group_1799	membrane protein	4	accessory
group_1800	catalase	4	accessory
group_1801	hypothetical protein	4	accessory
group_1802	superoxide dismutase [Cu-Zn]	4	accessory
group_1803	DTW domain-containing protein	4	accessory
group_1804	hypothetical protein	4	accessory
group_1805	hypothetical protein	4	accessory
group_1806	hypothetical protein	4	accessory
group_1807	fructokinase	4	accessory
group_1808	mannose-6-phosphate isomerase	4	accessory
group_1809	lipopolysaccharide biosynthesis protein RfbV	4	accessory
wbpY	glycosyltransferase WbpY	4	accessory
group_1811	GDP-6-deoxy-D-lyxo-4-hexulose reductase	4	accessory
gmd	GDP-mannose 4,6-dehydratase	4	accessory
group_1813	hypothetical protein	4	accessory
group_1814	glycosyl transferase	4	accessory
group_1815	hypothetical protein	4	accessory
group_1816	hypothetical protein	4	accessory
group_1817	dTDP-4-amino-4,6-dideoxy-D-glucose transaminase	4	accessory

group_1818	AMP-dependent synthetase	4	accessory
group_1819	oxidoreductase	4	accessory
group_1820	hypothetical protein	4	accessory
group_1821	short-chain dehydrogenase	4	accessory
acpP3	acyl carrier protein	4	accessory
group_1823	putative 3-oxoacyl-[acyl-carrier-protein] synthase 3	4	accessory
group_1824	hypothetical protein	4	accessory
group_1825	dTDP-glucose 4,6-dehydratase	4	accessory
group_1826	hypothetical protein	4	accessory
group_1828	hypothetical protein	4	accessory
wzm	transport permease protein	4	accessory
rfbB	phosphomannomutase	4	accessory
group_1831	mannose-1-phosphate guanylyltransferase	4	accessory
group_1832	hexapeptide transferase	4	accessory
group_1833	polymerase	4	accessory
group_1836	hypothetical protein	4	accessory
group_1837	hypothetical protein	4	accessory
group_1839	prepilin-type N-terminal cleavage/methylation domain-containing protein	4	accessory
group_1840	hypothetical protein	4	accessory
group_1841	ATP-dependent endonuclease	4	accessory
group_1842	DNA/RNA helicase superfamily I	4	accessory
group_1843	hypothetical protein	4	accessory
group_1845	capsular polysaccharide biosynthesis protein CapK	4	accessory
group_1846	hypothetical protein	4	accessory
group_1847	polysaccharide biosynthesis protein	4	accessory
group_1848	porin	4	accessory
group_1853	permease	4	accessory
group_1854	hypothetical protein	4	accessory
group_1855	hypothetical protein	4	accessory
group_1857	modification methylase, putative	4	accessory
group_1858	hypothetical protein	4	accessory
hchA	protein deglycase HchA	4	accessory
group_1860	LuxR family transcriptional regulator	4	accessory
group_1861	permease	4	accessory
group_1862	CdaR family transcriptional regulator	4	accessory
group_1863	hypothetical protein	4	accessory
group_1864	MSHA pilin protein MshA	4	accessory
group_1875	integrase	4	accessory
group_1876	hypothetical protein	4	accessory

group_1877	hypothetical protein	4	accessory
group_1878	hypothetical protein	4	accessory
group_1879	hypothetical protein	4	accessory
group_1880	transcriptional regulator	4	accessory
group_1881	hypothetical protein	4	accessory
group_1882	hypothetical protein	4	accessory
group_1883	hypothetical protein	4	accessory
group_1884	hypothetical protein	4	accessory
group_1885	hypothetical protein	4	accessory
group_1886	hypothetical protein	4	accessory
group_1887	hypothetical protein	4	accessory
group_1888	hypothetical protein	4	accessory
group_1889	hypothetical protein	4	accessory
R	lysozyme	4	accessory
group_1891	hypothetical protein	4	accessory
group_1892	recombinase	4	accessory
group_1893	hypothetical protein	4	accessory
group_1894	hypothetical protein	4	accessory
group_1895	hypothetical protein	4	accessory
group_1896	phage portal protein	4	accessory
group_1897	hypothetical protein	4	accessory
group_1898	hypothetical protein	4	accessory
group_1899	hypothetical protein	4	accessory
group_1900	hypothetical protein	4	accessory
group_1901	hypothetical protein	4	accessory
gpV	baseplate assembly protein	4	accessory
gpW	baseplate assembly protein	4	accessory
J	baseplate assembly protein	4	accessory
group_1905	hypothetical protein	4	accessory
group_1906	hypothetical protein	4	accessory
group_1907	hypothetical protein	4	accessory
group_1908	hypothetical protein	4	accessory
group_1909	bacteriophage protein	4	accessory
group_1910	hypothetical protein	4	accessory
group_1911	hypothetical protein	4	accessory
group_1912	hypothetical protein	4	accessory
group_1913	tail protein X	4	accessory
D	phage late control protein	4	accessory
group_1916	hypothetical protein	4	accessory

group_1917	hypothetical protein	4	accessory
group_1918	hemagglutinin	4	accessory
group_1919	methyltransferase	4	accessory
group_1920	hypothetical protein	4	accessory
group_1921	hypothetical protein	4	accessory
group_1922	hypothetical protein	4	accessory
group_1923	acetyltransferase	4	accessory
group_1924	hypothetical protein	4	accessory
group_1925	hypothetical protein	4	accessory
group_1926	nuclease	4	accessory
group_1927	hypothetical protein	4	accessory
group_1928	hypothetical protein	4	accessory
group_1929	hypothetical protein	4	accessory
group_1930	activator of HSP90 ATPase	4	accessory
group_1931	hypothetical protein	4	accessory
group_1932	hypothetical protein	4	accessory
group_1933	hypothetical protein	4	accessory
group_1934	acetyltransferase	4	accessory
group_1936	hypothetical protein	4	accessory
group_1937	N-acetyltransferase	4	accessory
group_1938	hypothetical protein	4	accessory
group_1940	hypothetical protein	4	accessory
group_1942	hypothetical protein	4	accessory
group_1943	hypothetical protein	4	accessory
group_1944	hypothetical protein	4	accessory
group_1946	hypothetical protein	4	accessory
group_1947	hypothetical protein	4	accessory
group_1948	acetyltransferase	4	accessory
group_1949	hypothetical protein	4	accessory
group_1951	hypothetical protein	4	accessory
group_1952	hypothetical protein	4	accessory
group_1953	isomerase	4	accessory
group_1954	hypothetical protein	4	accessory
group_1955	hypothetical protein	4	accessory
group_1956	hypothetical protein	4	accessory
group_1957	hypothetical protein	4	accessory
group_1958	DUF1289 domain-containing protein	4	accessory
group_1961	hypothetical protein	4	accessory
group_1963	sulfate-binding protein	4	accessory

group_1965	hypothetical protein	4	accessory
group_1966	antibiotic biosynthesis monooxygenase	4	accessory
group_1967	hypothetical protein	4	accessory
group_1968	hypothetical protein	4	accessory
group_1969	hypothetical protein	4	accessory
group_1970	hypothetical protein	4	accessory
group_1971	hypothetical protein	4	accessory
group_1972	hypothetical protein	4	accessory
group_1973	hypothetical protein	4	accessory
group_1974	hypothetical protein	4	accessory
group_1975	hypothetical protein	4	accessory
group_1976	polyketide cyclase	4	accessory
group_1977	alpha/beta hydrolase	4	accessory
group_1978	hypothetical protein	4	accessory
group_1979	hypothetical protein	4	accessory
group_1980	hypothetical protein	4	accessory
group_1981	hypothetical protein	4	accessory
group_1982	plasmid stabilization protein ParE	4	accessory
group_1983	antitoxin	4	accessory
group_1984	hypothetical protein	4	accessory
group_1985	hypothetical protein	4	accessory
group_1986	hydrolase	4	accessory
group_1987	hypothetical protein	4	accessory
group_1988	hypothetical protein	4	accessory
group_1995	hypothetical protein	4	accessory
group_1997	hypothetical protein	4	accessory
group_1998	hypothetical protein	4	accessory
group_2027	hypothetical protein	4	accessory
group_2060	membrane protein	4	accessory
ompK	outer membrane protein OmpK	4	accessory
group_2089	aminotransferase	4	accessory
group_2109	hypothetical protein	4	accessory
group_2110	hypothetical protein	4	accessory
group_2111	Lex2B	4	accessory
group_2112	hypothetical protein	4	accessory
group_2113	hypothetical protein	4	accessory
group_2142	hypothetical protein	4	accessory
group_2156	hypothetical protein	4	accessory
group_2164	glyoxalase	4	accessory

group_2168	hypothetical protein	4	accessory
group_2172	zinc chelation protein SecC	4	accessory
group_2175	hypothetical protein	4	accessory
group_2188	outer membrane protein OmpK	4	accessory
group_2299	outer membrane protein	4	accessory
group_2323	endonuclease	4	accessory
group_2359	hypothetical protein	4	accessory
group_2383	protein Syd	4	accessory
group_2424	hypothetical protein	4	accessory
group_2425	hypothetical protein	4	accessory
group_2477	hypothetical protein	4	accessory
group_2719	hypothetical protein	4	accessory
group_2745	hypothetical protein	4	accessory
group_2746	hypothetical protein	4	accessory
group_2747	hypothetical protein	4	accessory
group_2762	hypothetical protein	4	accessory
group_3050	hypothetical protein	4	accessory
group_3191	Lex2B	4	accessory
group_3210	ribonuclease H	4	accessory
group_459	hypothetical protein	4	accessory
yagK	hypothetical protein	4	accessory
group_1071	hypothetical protein	3	accessory
group_1076	hypothetical protein	3	accessory
group_1077	hypothetical protein	3	accessory
group_1078	hypothetical protein	3	accessory
group_1081	hexapeptide transferase	3	accessory
group_1087	hypothetical protein	3	accessory
group_1089	hypothetical protein	3	accessory
group_1093	integrase	3	accessory
group_1095	hypothetical protein	3	accessory
group_1099	hypothetical protein	3	accessory
group_1100	hypothetical protein	3	accessory
group_1102	hypothetical protein	3	accessory
group_1103	hypothetical protein	3	accessory
group_1105	hypothetical protein	3	accessory
group_1106	hypothetical protein	3	accessory
group_1108	hypothetical protein	3	accessory
group_1109	hypothetical protein	3	accessory
group_1110	peptidase	3	accessory

group_1123	type I-F CRISPR-associated protein Csy1	3	accessory
group_1132	hypothetical protein	3	accessory
trhC	plasmid transfer protein	3	accessory
group_1146	hypothetical protein	3	accessory
group_1150	hypothetical protein	3	accessory
group_1151	hemolysin	3	accessory
group_1152	ABC transporter substrate-binding protein	3	accessory
group_1153	nickel/cobalt efflux system	3	accessory
group_1154	hypothetical protein	3	accessory
group_1158	hypothetical protein	3	accessory
group_1159	hypothetical protein	3	accessory
group_1160	hypothetical protein	3	accessory
group_1161	hypothetical protein	3	accessory
group_1162	hypothetical protein	3	accessory
group_1163	hypothetical protein	3	accessory
group_1164	hypothetical protein	3	accessory
group_1165	hypothetical protein	3	accessory
group_1171	hypothetical protein	3	accessory
group_1795	hypothetical protein	3	accessory
group_1827	hypothetical protein	3	accessory
group_1856	hypothetical protein	3	accessory
group_1962	hypothetical protein	3	accessory
group_1992	PTS sugar transporter subunit IIA	3	accessory
group_1996	reverse transcriptase	3	accessory
group_200	hypothetical protein	3	accessory
group_201	hypothetical protein	3	accessory
group_2015	hypothetical protein	3	accessory
group_2062	hypothetical protein	3	accessory
group_2063	hypothetical protein	3	accessory
group_2065	hypothetical protein	3	accessory
ydfG	hypothetical protein	3	accessory
group_2067	maltoporin	3	accessory
group_2069	hypothetical protein	3	accessory
group_2070	hypothetical protein	3	accessory
group_2080	hypothetical protein	3	accessory
group_2090	hypothetical protein	3	accessory
group_2091	3-oxoacyl-ACP reductase	3	accessory
epsM_1	putative acetyltransferase EpsM	3	accessory
group_2093	hypothetical protein	3	accessory

group_2094	acyl-protein synthetase	3	accessory
group_2095	acyl carrier protein	3	accessory
group_2096	sugar transferase	3	accessory
group_2097	glycosyltransferase WbuB	3	accessory
group_2098	hypothetical protein	3	accessory
group_2099	oxidoreductase	3	accessory
hisF2	putative imidazole glycerol phosphate synthase subunit hisF2	3	accessory
hisH2	imidazole glycerol phosphate synthase subunit HisH 2	3	accessory
wbpG	LPS biosynthesis protein WbpG	3	accessory
wecC_1	UDP-N-acetyl-D-mannosamine dehydrogenase	3	accessory
rffE	UDP-N-acetylglucosamine 2-epimerase	3	accessory
group_2108	hypothetical protein	3	accessory
group_2121	polysaccharide biosynthesis protein	3	accessory
group_2122	hypothetical protein	3	accessory
group_2123	polysaccharide biosynthesis protein	3	accessory
group_2124	hypothetical protein	3	accessory
group_2125	capsular polysaccharide biosynthesis protein CapK	3	accessory
group_2143	type I-F CRISPR-associated endoribonuclease Cas6/Csy4	3	accessory
group_2144	hypothetical protein	3	accessory
group_2145	hypothetical protein	3	accessory
group_2146	hypothetical protein	3	accessory
group_2167	hypothetical protein	3	accessory
group_2177	hypothetical protein	3	accessory
group_2192	hypothetical protein	3	accessory
group_2194	hypothetical protein	3	accessory
group_2219	hypothetical protein	3	accessory
group_2220	hypothetical protein	3	accessory
group_2222	hypothetical protein	3	accessory
trhU	plasmid transfer protein	3	accessory
group_2233	GGDEF domain-containing protein	3	accessory
group_2234	hypothetical protein	3	accessory
group_2235	cyclic nucleotide-binding protein	3	accessory
group_2236	3'-5' exonuclease	3	accessory
group_2237	BCCT family transporter	3	accessory
group_2238	hypothetical protein	3	accessory
group_2240	hypothetical protein	3	accessory
group_2245	DNA repair protein RadC	3	accessory
group_2248	hypothetical protein	3	accessory
group_2249	hypothetical protein	3	accessory

group_2250	diguanylate cyclase	3	accessory
group_2251	hypothetical protein	3	accessory
group_2252	diguanylate cyclase response regulator	3	accessory
traH	conjugal transfer protein TraH	3	accessory
group_2265	hypothetical protein	3	accessory
group_2266	hypothetical protein	3	accessory
group_2267	hypothetical protein	3	accessory
group_2268	hypothetical protein	3	accessory
group_2269	hypothetical protein	3	accessory
group_2297	LysR family transcriptional regulator	3	accessory
group_2381	CRISPR-associated protein Csy3	3	accessory
group_2382	hypothetical protein	3	accessory
group_2411	hypothetical protein	3	accessory
group_2414	protein kinase	3	accessory
group_2462	hypothetical protein	3	accessory
group_2465	hypothetical protein	3	accessory
group_2475	toxin HigB-2	3	accessory
group_2495	hypothetical protein	3	accessory
group_2498	N-acetyltransferase	3	accessory
group_2503	hypothetical protein	3	accessory
group_2516	universal stress protein	3	accessory
group_2713	hypothetical protein	3	accessory
group_2867	hypothetical protein	3	accessory
group_2879	conjugative coupling factor TraD, PFGI-1 class	3	accessory
group_2892	hypothetical protein	3	accessory
group_2893	hypothetical protein	3	accessory
group_2894	hypothetical protein	3	accessory
group_2895	hypothetical protein	3	accessory
group_2896	hypothetical protein	3	accessory
group_2897	hypothetical protein	3	accessory
group_2898	hypothetical protein	3	accessory
group_2899	hypothetical protein	3	accessory
group_2900	hypothetical protein	3	accessory
group_2902	hypothetical protein	3	accessory
group_2903	hypothetical protein	3	accessory
group_2904	hypothetical protein	3	accessory
group_2905	hypothetical protein	3	accessory
group_2906	hypothetical protein	3	accessory
group_2907	hypothetical protein	3	accessory

group_2908	hypothetical protein	3	accessory
group_2909	hypothetical protein	3	accessory
group_2910	hypothetical protein	3	accessory
group_2912	hypothetical protein	3	accessory
group_2913	hypothetical protein	3	accessory
group_2914	hypothetical protein	3	accessory
group_2915	hypothetical protein	3	accessory
group_2916	hypothetical protein	3	accessory
group_2919	peroxiredoxin	3	accessory
group_2920	hypothetical protein	3	accessory
group_2921	hypothetical protein	3	accessory
group_2922	hypothetical protein	3	accessory
group_2923	hypothetical protein	3	accessory
group_2924	hypothetical protein	3	accessory
group_2925	hypothetical protein	3	accessory
group_2926	hypothetical protein	3	accessory
group_2927	cell division protein	3	accessory
group_2928	GGDEF domain-containing protein	3	accessory
group_2929	hypothetical protein	3	accessory
group_2930	hypothetical protein	3	accessory
group_2931	hypothetical protein	3	accessory
group_2932	hypothetical protein	3	accessory
group_2933	hypothetical protein	3	accessory
group_2934	site-specific DNA-methyltransferase type I modification	3	accessory
group_2935	DUF1016 domain-containing protein	3	accessory
group_2936	restriction endonuclease	3	accessory
group_2937	hypothetical protein	3	accessory
group_2938	(p)ppGpp synthetase	3	accessory
group_2939	DUF4935 domain-containing protein	3	accessory
group_2940	hypothetical protein	3	accessory
group_2941	chromosome replication initiation inhibitor protein	3	accessory
yggA	amino acid transporter	3	accessory
group_2943	transcriptional regulator	3	accessory
group_2944	N-acetyltransferase	3	accessory
group_2945	hypothetical protein	3	accessory
group_2946	LysR family transcriptional regulator	3	accessory
group_2947	hypothetical protein	3	accessory
group_2948	ABC transporter permease	3	accessory
group_2949	iron ABC transporter	3	accessory

group_2950	hypothetical protein	3	accessory
group_2951	hypothetical protein	3	accessory
group_2952	hypothetical protein	3	accessory
group_2953	hypothetical protein	3	accessory
group_2955	hypothetical protein	3	accessory
group_2956	hypothetical protein	3	accessory
group_2957	phosphate ABC transporter substrate-binding protein	3	accessory
group_2958	hypothetical protein	3	accessory
gatY	class II aldolase, tagatose bisphosphate family protein	3	accessory
nagA-1	N-acetylglucosamine-6-phosphate deacetylase	3	accessory
group_2961	PTS N-acetylgalactosamine transporter subunit IIA	3	accessory
group_2962	PTS N-acetylgalactosamine transporter subunit IID	3	accessory
group_2963	PTS N-acetylgalactosamine transporter subunit IIC	3	accessory
agaV	PTS N-acetylgalactosamine transporter subunit IIB	3	accessory
agaS	tagatose-6-phosphate ketose isomerase	3	accessory
kbaZ	D-tagatose-1,6-bisphosphate aldolase subunit KbaZ	3	accessory
agaR_1	DeoR family transcriptional regulator	3	accessory
agaR_2	DeoR family transcriptional regulator	3	accessory
group_2969	long-chain fatty acid transporter	3	accessory
group_2970	GGDEF domain-containing protein	3	accessory
group_2971	hypothetical protein	3	accessory
group_2972	hypothetical protein	3	accessory
group_2973	hypothetical protein	3	accessory
group_2974	serine/threonine protein phosphatase	3	accessory
group_2975	hypothetical protein	3	accessory
group_2976	hypothetical protein	3	accessory
group_2977	hypothetical protein	3	accessory
group_2978	transcriptional regulator	3	accessory
kdpE	DNA-binding response regulator	3	accessory
group_2980	Trk system potassium uptake protein	3	accessory
group_2981	potassium transporter TrkA	3	accessory
group_2982	N-acetyltransferase	3	accessory
group_2983	hypothetical protein	3	accessory
group_2984	multidrug resistance protein A	3	accessory
group_2985	hypothetical protein	3	accessory
group_2986	hypothetical protein	3	accessory
group_2987	hypothetical protein	3	accessory
group_2988	hypothetical protein	3	accessory
group_2989	type I restriction endonuclease EcoAI subunit S	3	accessory

group_2990	restriction endonuclease EcoEI subunit M	3	accessory
group_2991	hypothetical protein	3	accessory
group_2992	hypothetical protein	3	accessory
group_2993	translation elongation factor Ts	3	accessory
group_2994	hypothetical protein	3	accessory
group_2995	site-specific DNA-methyltransferase	3	accessory
group_2996	abi family protein	3	accessory
group_2997	DEAD/DEAH box helicase	3	accessory
group_2998	hypothetical protein	3	accessory
group_2999	hypothetical protein	3	accessory
intA	integrase	3	accessory
group_3001	DNA repair ATPase	3	accessory
group_3002	hypothetical protein	3	accessory
group_3003	hypothetical protein	3	accessory
group_3004	DNA-binding transcriptional regulator KdgR	3	accessory
group_3005	hypothetical protein	3	accessory
group_3006	ABC transporter ATP-binding protein	3	accessory
group_3007	peptide ABC transporter permease	3	accessory
group_3008	ABC transporter permease	3	accessory
group_3009	peptide ABC transporter substrate-binding protein	3	accessory
group_3010	hypothetical protein	3	accessory
aslB	anaerobic sulfatase-maturating enzyme homolog AslB	3	accessory
group_3012	arylsulfatase	3	accessory
group_3013	GGDEF domain-containing protein	3	accessory
group_3014	chloramphenicol resistance protein	3	accessory
lnbP	1,3-beta-galactosyl-N-acetylhexosamine phosphorylase	3	accessory
group_3016	glycosyl hydrolase	3	accessory
kduD1	2-deoxy-D-gluconate 3-dehydrogenase	3	accessory
group_3018	galactose-1-phosphate uridylyltransferase	3	accessory
group_3019	ketohydroxyglutarate aldolase	3	accessory
group_3020	2-dehydro-3-deoxygluconokinase	3	accessory
kduI	4-deoxy-L-threo-5-hexosulose-uronate ketol-isomerase	3	accessory
group_3022	hypothetical protein	3	accessory
group_3023	hypothetical protein	3	accessory
group_3024	twin-arginine translocation pathway signal	3	accessory
group_3025	hypothetical protein	3	accessory
group_3026	nucleotidyltransferase	3	accessory
group_3027	hypothetical protein	3	accessory
group_3028	beta-glucosidase	3	accessory

rhaR	AraC family transcriptional regulator	3	accessory
group_3030	maltoporin	3	accessory
group_3031	hypothetical protein	3	accessory
group_3032	hypothetical protein	3	accessory
galR	transcriptional regulator GalR	3	accessory
group_3034	hypothetical protein	3	accessory
group_3035	ABC transporter ATP-binding protein	3	accessory
group_3036	peptide ABC transporter ATP-binding protein	3	accessory
group_3037	peptide ABC transporter permease	3	accessory
group_3038	peptide ABC transporter permease	3	accessory
group_3039	ABC transporter substrate-binding protein	3	accessory
group_3040	hypothetical protein	3	accessory
group_3041	hypothetical protein	3	accessory
group_3042	type IV secretion protein Rhs	3	accessory
group_3043	sulfate transporter	3	accessory
group_3044	hypothetical protein	3	accessory
group_3045	membrane protein	3	accessory
group_3046	NADPH:quinone reductase	3	accessory
group_3047	thioredoxin	3	accessory
group_3048	NAD(P)H dehydrogenase (quinone)	3	accessory
group_3049	transcriptional regulator	3	accessory
group_3051	hypothetical protein	3	accessory
group_3055	ABC transporter substrate-binding protein	3	accessory
group_3056	serine protease	3	accessory
group_3057	porin	3	accessory
group_3058	hemolysin	3	accessory
group_3059	UDP-N-acetyl-D-mannosamine transferase	3	accessory
group_3060	hypothetical protein	3	accessory
group_3061	polysaccharide biosynthesis protein	3	accessory
group_3062	serine acetyltransferase	3	accessory
group_3063	glycosyl transferase	3	accessory
group_3064	hypothetical protein	3	accessory
group_3065	O-unit flippase	3	accessory
group_3066	hypothetical protein	3	accessory
group_3067	hypothetical protein	3	accessory
group_3068	hypothetical protein	3	accessory
group_3069	outer membrane protein U	3	accessory
pilA	pilin	3	accessory
gmhB_2	D,D-heptose 1,7-bisphosphate phosphatase	3	accessory

hddC	D-glycero-D-manno-heptose 1-phosphate guanosyltransferase	3	accessory
gmhA2	phosphoheptose isomerase 2	3	accessory
group_3178	dehydrogenase	3	accessory
wbaD	glycosyl transferase family 1	3	accessory
group_3180	hypothetical protein	3	accessory
group_3181	hypothetical protein	3	accessory
group_3182	polysaccharide polymerase	3	accessory
group_3183	glycosyl transferase	3	accessory
group_3184	hypothetical protein	3	accessory
rmlB	dTDP-glucose 4,6-dehydratase	3	accessory
rfbC	dTDP-4-dehydrorhamnose 3,5-epimerase	3	accessory
group_3187	glucose-1-phosphate thymidylyltransferase	3	accessory
group_3188	dTDP-glucose 4,6-dehydratase	3	accessory
group_3189	hypothetical protein	3	accessory
group_3190	hypothetical protein	3	accessory
group_3192	hypothetical protein	3	accessory
group_3193	methionyl-tRNA formyltransferase	3	accessory
group_3194	hypothetical protein	3	accessory
group_3195	ligase	3	accessory
group_3196	hypothetical protein	3	accessory
group_3197	hypothetical protein	3	accessory
group_3198	hypothetical protein	3	accessory
group_3199	OtnA protein	3	accessory
group_3200	LPS biosynthesis protein	3	accessory
group_3201	hypothetical protein	3	accessory
group_3202	hypothetical protein	3	accessory
group_3203	hypothetical protein	3	accessory
group_3204	hypothetical protein	3	accessory
group_3205	MarR family transcriptional regulator	3	accessory
group_3206	hypothetical protein	3	accessory
group_3207	hypothetical protein	3	accessory
group_3208	hypothetical protein	3	accessory
group_3211	hypothetical protein	3	accessory
group_3212	hypothetical protein	3	accessory
group_3213	type IV pilin	3	accessory
group_3214	hypothetical protein	3	accessory
group_3215	peptidase M23	3	accessory
group_3216	hypothetical protein	3	accessory
group_3217	hypothetical protein	3	accessory

group_3218	UPF0758 protein	3	accessory
group_3219	hypothetical protein	3	accessory
group_3220	AraC family transcriptional regulator	3	accessory
group_3221	methyl-accepting chemotaxis protein	3	accessory
group_3222	diguanylate phosphodiesterase	3	accessory
group_3223	hypothetical protein	3	accessory
group_3224	hypothetical protein	3	accessory
group_3225	hypothetical protein	3	accessory
group_3226	hypothetical protein	3	accessory
group_3227	DEAD/DEAH box helicase	3	accessory
group_3228	hypothetical protein	3	accessory
mcrC	5-methylcytosine-specific restriction system specificity protein McrC	3	accessory
group_3230	paraquat-inducible protein B	3	accessory
group_3231	hypothetical protein	3	accessory
group_3232	membrane protein	3	accessory
group_3233	hypothetical protein	3	accessory
group_3234	hypothetical protein	3	accessory
group_3235	hypothetical protein	3	accessory
group_3236	hypothetical protein	3	accessory
group_3237	hypothetical protein	3	accessory
group_3238	hypothetical protein	3	accessory
group_3239	sex pilus assembly and mating pair formation protein TrbC	3	accessory
group_3240	conjugal transfer protein TraF	3	accessory
group_3241	hypothetical protein	3	accessory
group_3242	conjugal transfer protein TraF	3	accessory
group_3243	pilus assembly protein	3	accessory
group_3244	hypothetical protein	3	accessory
group_3245	hypothetical protein	3	accessory
group_3246	hypothetical protein	3	accessory
group_3247	hypothetical protein	3	accessory
group_3248	hypothetical protein	3	accessory
group_3249	hypothetical protein	3	accessory
group_3250	hypothetical protein	3	accessory
group_3251	hypothetical protein	3	accessory
group_3252	hypothetical protein	3	accessory
group_3253	hypothetical protein	3	accessory
group_3254	hypothetical protein	3	accessory
group_3255	hypothetical protein	3	accessory
group_3256	hypothetical protein	3	accessory

group_3257	hypothetical protein	3	accessory
group_3258	hypothetical protein	3	accessory
group_3259	hypothetical protein	3	accessory
group_3260	hypothetical protein	3	accessory
group_3261	hypothetical protein	3	accessory
group_3262	hypothetical protein	3	accessory
group_3263	hypothetical protein	3	accessory
group_3264	hypothetical protein	3	accessory
group_3265	hypothetical protein	3	accessory
group_3266	hypothetical protein	3	accessory
group_3267	hypothetical protein	3	accessory
group_3268	hypothetical protein	3	accessory
group_3269	hypothetical protein	3	accessory
group_3270	hypothetical protein	3	accessory
group_3271	conjugative relaxase	3	accessory
group_3272	hypothetical protein	3	accessory
group_3273	hypothetical protein	3	accessory
group_3274	hypothetical protein	3	accessory
group_3275	hypothetical protein	3	accessory
group_3276	hypothetical protein	3	accessory
group_3277	hypothetical protein	3	accessory
group_3278	hypothetical protein	3	accessory
group_3279	hypothetical protein	3	accessory
group_3280	hypothetical protein	3	accessory
group_3281	hypothetical protein	3	accessory
group_3282	hypothetical protein	3	accessory
group_3283	hypothetical protein	3	accessory
group_3284	hypothetical protein	3	accessory
group_3285	hypothetical protein	3	accessory
group_3286	DNA repair protein RadC	3	accessory
group_3287	hypothetical protein	3	accessory
group_3288	hypothetical protein	3	accessory
group_3289	hypothetical protein	3	accessory
group_3290	hypothetical protein	3	accessory
group_3291	hypothetical protein	3	accessory
group_3292	hypothetical protein	3	accessory
group_3293	hypothetical protein	3	accessory
group_3294	hypothetical protein	3	accessory
group_3295	hypothetical protein	3	accessory

group_3296	hypothetical protein	3	accessory
group_3297	hypothetical protein	3	accessory
group_3298	hypothetical protein	3	accessory
group_3299	hypothetical protein	3	accessory
group_3300	hypothetical protein	3	accessory
group_3301	hypothetical protein	3	accessory
group_3302	hypothetical protein	3	accessory
group_3303	hypothetical protein	3	accessory
group_3304	hypothetical protein	3	accessory
group_3305	hypothetical protein	3	accessory
group_3306	hypothetical protein	3	accessory
group_3307	hypothetical protein	3	accessory
group_3308	hypothetical protein	3	accessory
group_3309	hypothetical protein	3	accessory
group_3310	hypothetical protein	3	accessory
group_3311	hypothetical protein	3	accessory
group_3312	hypothetical protein	3	accessory
group_3313	hypothetical protein	3	accessory
group_3314	hypothetical protein	3	accessory
group_3315	hypothetical protein	3	accessory
group_3316	hypothetical protein	3	accessory
group_3317	hypothetical protein	3	accessory
group_3318	hypothetical protein	3	accessory
group_3319	hypothetical protein	3	accessory
group_3320	hypothetical protein	3	accessory
group_3321	hypothetical protein	3	accessory
group_3322	hypothetical protein	3	accessory
group_3323	membrane protein	3	accessory
group_3324	hypothetical protein	3	accessory
group_3325	hypothetical protein	3	accessory
group_3326	N-acetyltransferase	3	accessory
group_3327	permease	3	accessory
group_3328	hypothetical protein	3	accessory
group_3329	hypothetical protein	3	accessory
group_3330	hypothetical protein	3	accessory
group_3331	DUF1289 domain-containing protein	3	accessory
group_3332	hypothetical protein	3	accessory
group_3333	hypothetical protein	3	accessory
group_3334	hypothetical protein	3	accessory

group_3335	hypothetical protein	3	accessory
group_3336	hypothetical protein	3	accessory
group_3337	biphenyl-2,3-diol 1,2-dioxygenase	3	accessory
group_3338	hypothetical protein	3	accessory
group_3339	hypothetical protein	3	accessory
rflA	hypothetical protein	3	accessory
group_3341	hypothetical protein	3	accessory
group_3342	hypothetical protein	3	accessory
group_3343	hypothetical protein	3	accessory
group_3344	transcriptional regulator	3	accessory
group_3345	glyoxalase	3	accessory
group_3346	glyoxalase	3	accessory
group_3347	hypothetical protein	3	accessory
group_3348	hypothetical protein	3	accessory
group_3349	acetyltransferase	3	accessory
group_3350	pyridoxamine 5'-phosphate oxidase-like FMN-binding protein	3	accessory
yedJ	phosphohydrolase	3	accessory
group_3353	hypothetical protein	3	accessory
group_3354	hypothetical protein	3	accessory
group_3355	hypothetical protein	3	accessory
group_3356	phosphohydrolase	3	accessory
group_3357	hypothetical protein	3	accessory
group_3358	hypothetical protein	3	accessory
group_3359	hypothetical protein	3	accessory
group_3360	hypothetical protein	3	accessory
group_3361	hypothetical protein	3	accessory
group_3362	hypothetical protein	3	accessory
soj	sporulation initiation inhibitor protein Soj	3	accessory
group_3364	hypothetical protein	3	accessory
group_3365	hypothetical protein	3	accessory
group_3366	hypothetical protein	3	accessory
group_3368	hypothetical protein	3	accessory
group_3370	hypothetical protein	3	accessory
group_3371	hypothetical protein	3	accessory
group_3372	site-specific DNA-methyltransferase (adenine-specific)	3	accessory
group_3373	hypothetical protein	3	accessory
group_3374	hypothetical protein	3	accessory
group_3376	hypothetical protein	3	accessory
group_3377	hypothetical protein	3	accessory

group_3378	hypothetical protein	3	accessory
group_3379	hypothetical protein	3	accessory
group_3380	hypothetical protein	3	accessory
group_3382	hypothetical protein	3	accessory
group_3383	hypothetical protein	3	accessory
group_3384	hypothetical protein	3	accessory
group_3385	acetyltransferase	3	accessory
group_3386	hypothetical protein	3	accessory
group_3387	DUF645 domain-containing protein	3	accessory
group_3388	hypothetical protein	3	accessory
group_3389	hypothetical protein	3	accessory
group_3390	resolvase	3	accessory
group_3391	hypothetical protein	3	accessory
group_3393	DNA methyltransferase	3	accessory
group_3394	hypothetical protein	3	accessory
vagC	virulence factor	3	accessory
group_3396	hypothetical protein	3	accessory
group_3397	hypothetical protein	3	accessory
group_3398	hypothetical protein	3	accessory
group_3399	hypothetical protein	3	accessory
group_3400	hypothetical protein	3	accessory
group_3401	hypothetical protein	3	accessory
group_3402	hypothetical protein	3	accessory
group_3403	hypothetical protein	3	accessory
group_3405	conjugal transfer protein TraB	3	accessory
group_3406	hypothetical protein	3	accessory
group_3407	hypothetical protein	3	accessory
group_3408	hypothetical protein	3	accessory
group_3409	hypothetical protein	3	accessory
group_3410	protein disulfide-isomerase	3	accessory
group_3411	hypothetical protein	3	accessory
group_3412	hypothetical protein	3	accessory
group_3413	hypothetical protein	3	accessory
group_3414	DNA-binding protein	3	accessory
group_3415	hypothetical protein	3	accessory
group_3416	hypothetical protein	3	accessory
group_3417	hypothetical protein	3	accessory
group_3418	hypothetical protein	3	accessory
group_3419	acetyltransferase	3	accessory

group_3420	hypothetical protein	3	accessory
group_3421	RDD family protein	3	accessory
group_3422	hypothetical protein	3	accessory
group_3423	hypothetical protein	3	accessory
group_3424	hypothetical protein	3	accessory
group_3425	hypothetical protein	3	accessory
group_3426	hypothetical protein	3	accessory
group_3427	hypothetical protein	3	accessory
group_3428	hypothetical protein	3	accessory
group_3429	hypothetical protein	3	accessory
group_3430	hypothetical protein	3	accessory
group_3431	oxidoreductase	3	accessory
group_3432	hypothetical protein	3	accessory
group_3433	TetR family transcriptional regulator	3	accessory
group_3434	HPP family protein	3	accessory
group_3435	hypothetical protein	3	accessory
group_3436	hypothetical protein	3	accessory
group_3437	hypothetical protein	3	accessory
group_3442	hypothetical protein	3	accessory
group_3443	hypothetical protein	3	accessory
group_3444	hypothetical protein	3	accessory
group_3445	hypothetical protein	3	accessory
group_3446	hypothetical protein	3	accessory
group_3447	hypothetical protein	3	accessory
group_3448	hypothetical protein	3	accessory
group_3449	hypothetical protein	3	accessory
group_3450	S-(hydroxymethyl)glutathione synthase	3	accessory
group_3451	hypothetical protein	3	accessory
group_3452	hypothetical protein	3	accessory
group_3453	hypothetical protein	3	accessory
group_3454	alpha/beta hydrolase	3	accessory
group_3455	N-acetyltransferase	3	accessory
group_3456	hypothetical protein	3	accessory
group_3457	hypothetical protein	3	accessory
ntpA	NUDIX pyrophosphatase	3	accessory
group_3459	hypothetical protein	3	accessory
group_3460	hypothetical protein	3	accessory
rimL	ribosomal-protein-L7/L12-serine acetyltransferase	3	accessory
group_3462	hypothetical protein	3	accessory

group_3463	N-acetyltransferase	3	accessory
group_3464	hypothetical protein	3	accessory
mcbG	hypothetical protein	3	accessory
group_3466	hypothetical protein	3	accessory
group_3467	hypothetical protein	3	accessory
group_3468	hypothetical protein	3	accessory
group_3469	hypothetical protein	3	accessory
group_3470	hypothetical protein	3	accessory
group_3471	hypothetical protein	3	accessory
group_3474	hypothetical protein	3	accessory
group_3475	hypothetical protein	3	accessory
group_3476	AraC family transcriptional regulator	3	accessory
group_3545	acetyl-CoA acetyltransferase	3	accessory
group_3732	hypothetical protein	3	accessory
group_3733	hypothetical protein	3	accessory
group_3734	hypothetical protein	3	accessory
group_3738	hypothetical protein	3	accessory
umuD_3	protein UmuD	3	accessory
group_478	DNA polymerase III subunit epsilon	3	accessory
group_479	hypothetical protein	3	accessory
group_480	hypothetical protein	3	accessory
group_481	hypothetical protein	3	accessory
group_482	type IV secretion protein Rhs	3	accessory
group_1074	hypothetical protein	2	accessory
group_1086	hypothetical protein	2	accessory
group_1092	putative kinase Y4dM	2	accessory
group_1094	hypothetical protein	2	accessory
group_1096	hypothetical protein	2	accessory
group_1097	hypothetical protein	2	accessory
group_1098	hypothetical protein	2	accessory
group_1101	N-acetyltransferase GCN5	2	accessory
group_1104	hypothetical protein	2	accessory
group_1107	hypothetical protein	2	accessory
wbpA	UDP-N-acetyl-d-glucosamine 6-dehydrogenase WbpA	2	accessory
group_1115	undecaprenyl-phosphate beta-N-acetyl-D-fucosaminephosphotransferase	2	accessory
group_1116	DNA-binding protein	2	accessory
group_1117	phage regulatory protein (CII)	2	accessory
group_1118	hypothetical protein	2	accessory
group_1119	hypothetical protein	2	accessory

group_1120	hypothetical protein	2	accessory
group_1121	hypothetical protein	2	accessory
group_1126	hypothetical protein	2	accessory
group_1134	hypothetical protein	2	accessory
group_1135	hypothetical protein	2	accessory
group_1141	ATP-binding protein	2	accessory
group_1144	hypothetical protein	2	accessory
group_1147	hypothetical protein	2	accessory
group_1149	trimethoprim-resistant dihydrofolate reductase DfrA7	2	accessory
group_1167	polysaccharide deacetylase	2	accessory
group_1168	hypothetical protein	2	accessory
group_1169	hypothetical protein	2	accessory
group_1170	hypothetical protein	2	accessory
group_1702	hypothetical protein	2	accessory
group_1993	hypothetical protein	2	accessory
group_1994	hypothetical protein	2	accessory
group_1999	hypothetical protein	2	accessory
group_2000	hypothetical protein	2	accessory
group_2001	hypothetical protein	2	accessory
group_2002	hypothetical protein	2	accessory
group_2007	outer membrane protein U	2	accessory
group_2008	hypothetical protein	2	accessory
group_2009	hypothetical protein	2	accessory
group_2010	hypothetical protein	2	accessory
group_2011	hypothetical protein	2	accessory
group_2012	hypothetical protein	2	accessory
group_2013	hypothetical protein	2	accessory
group_2014	hypothetical protein	2	accessory
group_2016	hypothetical protein	2	accessory
group_2017	hypothetical protein	2	accessory
group_2018	hypothetical protein	2	accessory
group_2019	hypothetical protein	2	accessory
group_2020	hypothetical protein	2	accessory
group_2021	ATPase	2	accessory
group_2022	hypothetical protein	2	accessory
group_2023	hypothetical protein	2	accessory
group_2024	hypothetical protein	2	accessory
group_2025	hypothetical protein	2	accessory
group_2026	hypothetical protein	2	accessory

group_2031	hypothetical protein	2	accessory
group_2032	hypothetical protein	2	accessory
group_2040	hypothetical protein	2	accessory
group_2041	hypothetical protein	2	accessory
group_2042	MATE family efflux transporter	2	accessory
group_2043	AraC family transcriptional regulator	2	accessory
group_2044	ABC transporter permease	2	accessory
group_2045	sugar ABC transporter permease	2	accessory
group_2046	cellobiose 2-epimerase	2	accessory
group_2047	sugar ABC transporter ATP-binding protein	2	accessory
group_2048	hypothetical protein	2	accessory
group_2049	transcriptional regulator	2	accessory
group_2050	phosphomannomutase	2	accessory
group_2051	glycosylase	2	accessory
group_2052	4-O-beta-D-mannosyl-D-glucose phosphorylase	2	accessory
group_2053	maltoporin	2	accessory
group_2054	methyl-accepting chemotaxis protein	2	accessory
group_2055	mannose-6-phosphate isomerase	2	accessory
group_2056	endo-1,4-beta-mannosidase	2	accessory
group_2057	sugar ABC transporter substrate-binding protein	2	accessory
group_2058	beta-mannosidase	2	accessory
group_2079	hypothetical protein	2	accessory
group_2084	hypothetical protein	2	accessory
group_2085	pilus assembly protein PilA	2	accessory
group_2087	tRNA-Leu	2	accessory
group_2103	hypothetical protein	2	accessory
group_2104	hypothetical protein	2	accessory
group_2105	hypothetical protein	2	accessory
int	integrase	2	accessory
group_2116	hypothetical protein	2	accessory
group_2117	hypothetical protein	2	accessory
group_2118	hypothetical protein	2	accessory
group_2119	hypothetical protein	2	accessory
group_2129	membrane protein	2	accessory
group_2130	hypothetical protein	2	accessory
group_2131	hypothetical protein	2	accessory
group_2132	hypothetical protein	2	accessory
group_2133	hypothetical protein	2	accessory
group_2134	helicase SNF2	2	accessory

group_2135	hypothetical protein	2	accessory
group_2136	site-specific DNA-methyltransferase	2	accessory
group_2137	type III restriction endonuclease subunit R	2	accessory
group_2138	protein-tyrosine-phosphatase	2	accessory
group_2139	cell envelope biogenesis protein AsmA	2	accessory
group_2154	hypothetical protein	2	accessory
group_2155	hypothetical protein	2	accessory
group_2157	hypothetical protein	2	accessory
group_2165	hypothetical protein	2	accessory
group_2166	hypothetical protein	2	accessory
group_2169	hypothetical protein	2	accessory
group_2170	hypothetical protein	2	accessory
group_2171	hypothetical protein	2	accessory
group_2176	hypothetical protein	2	accessory
ppdD	prepilin peptidase-dependent protein D	2	accessory
group_2191	transcriptional regulator	2	accessory
umuC	DNA polymerase V subunit UmuC	2	accessory
group_2196	WYL domain-containing protein	2	accessory
group_2197	hypothetical protein	2	accessory
group_2198	hypothetical protein	2	accessory
group_2199	hypothetical protein	2	accessory
group_2200	hypothetical protein	2	accessory
group_2202	DNA repair protein	2	accessory
group_2203	ATP-dependent Lon protease	2	accessory
group_2204	hypothetical protein	2	accessory
group_2205	hypothetical protein	2	accessory
group_2207	hypothetical protein	2	accessory
group_2208	hypothetical protein	2	accessory
group_2209	hypothetical protein	2	accessory
group_2210	ATP-binding protein	2	accessory
group_2211	hypothetical protein	2	accessory
group_2212	protein phosphatase	2	accessory
group_2213	hypothetical protein	2	accessory
group_2214	hypothetical protein	2	accessory
group_2215	hypothetical protein	2	accessory
group_2216	hypothetical protein	2	accessory
group_2241	hypothetical protein	2	accessory
group_2242	hypothetical protein	2	accessory
group_2243	cobalamin synthase	2	accessory

group_2244	hypothetical protein	2	accessory
group_2246	hypothetical protein	2	accessory
group_2247	hypothetical protein	2	accessory
group_2253	hypothetical protein	2	accessory
traF	type-F conjugative transfer system pilin assembly protein TraF	2	accessory
group_2256	conjugal transfer protein TraG	2	accessory
group_2257	transcriptional regulator	2	accessory
group_2258	hypothetical protein	2	accessory
group_2259	hypothetical protein	2	accessory
group_2260	IS630 family transposase	2	accessory
group_2261	hypothetical protein	2	accessory
group_2262	hypothetical protein	2	accessory
group_2279	sodium:calcium antiporter	2	accessory
group_2281	mechanosensitive ion channel protein	2	accessory
group_2333	replication protein A	2	accessory
group_2334	hypothetical protein	2	accessory
group_2335	hypothetical protein	2	accessory
group_2336	hypothetical protein	2	accessory
group_2337	hypothetical protein	2	accessory
group_2338	hypothetical protein	2	accessory
gpO	phage capsid scaffolding protein	2	accessory
group_2340	major capsid protein	2	accessory
group_2341	terminase	2	accessory
group_2342	head completion/stabilization protein (GpL) from bacteriophage origin	2	accessory
group_2343	hypothetical protein	2	accessory
group_2344	hypothetical protein	2	accessory
group_2345	phage tail protein	2	accessory
group_2346	tail protein	2	accessory
group_2347	hypothetical protein	2	accessory
group_2348	hypothetical protein	2	accessory
group_2349	lysozyme	2	accessory
group_2350	hypothetical protein	2	accessory
group_2351	hypothetical protein	2	accessory
group_2352	hypothetical protein	2	accessory
group_2353	hypothetical protein	2	accessory
group_2354	hypothetical protein	2	accessory
group_2355	hypothetical protein	2	accessory
group_2356	hypothetical protein	2	accessory
group_2358	hypothetical protein	2	accessory

group_2372	hypothetical protein	2	accessory
group_2373	hypothetical protein	2	accessory
group_2380	hypothetical protein	2	accessory
group_2393	MSHA pilin protein MshA	2	accessory
group_2403	hypothetical protein	2	accessory
group_2404	hypothetical protein	2	accessory
group_2405	long-chain fatty acid transporter	2	accessory
group_2412	hypothetical protein	2	accessory
group_2464	hypothetical protein	2	accessory
group_2466	hypothetical protein	2	accessory
group_2467	hypothetical protein	2	accessory
group_2468	hypothetical protein	2	accessory
group_2469	hypothetical protein	2	accessory
group_2470	hypothetical protein	2	accessory
group_2471	hypothetical protein	2	accessory
group_2474	hypothetical protein	2	accessory
group_2478	hypothetical protein	2	accessory
group_2480	DNA-damage-inducible protein J	2	accessory
group_2483	hypothetical protein	2	accessory
group_2484	hypothetical protein	2	accessory
group_2486	hypothetical protein	2	accessory
group_2487	hypothetical protein	2	accessory
group_2491	hypothetical protein	2	accessory
group_2494	hypothetical protein	2	accessory
group_2496	hypothetical protein	2	accessory
group_2497	hypothetical protein	2	accessory
group_2499	hypothetical protein	2	accessory
group_2717	hypothetical protein	2	accessory
group_2735	porin	2	accessory
group_2763	hypothetical protein	2	accessory
group_2765	hypothetical protein	2	accessory
group_2795	hypothetical protein	2	accessory
group_2851	hypothetical protein	2	accessory
group_2864	hypothetical protein	2	accessory
group_2874	hypothetical protein	2	accessory
group_2901	phosphohydrolase	2	accessory
group_2911	hypothetical protein	2	accessory
group_2954	hypothetical protein	2	accessory
group_3070	hypothetical protein	2	accessory

group_3071	hypothetical protein	2	accessory
group_3072	hypothetical protein	2	accessory
group_3073	hypothetical protein	2	accessory
group_3074	hypothetical protein	2	accessory
group_3075	hypothetical protein	2	accessory
group_3076	hypothetical protein	2	accessory
group_3077	hypothetical protein	2	accessory
group_3078	hypothetical protein	2	accessory
group_3079	hypothetical protein	2	accessory
group_3080	hypothetical protein	2	accessory
group_3081	hypothetical protein	2	accessory
group_3082	hypothetical protein	2	accessory
group_3083	hypothetical protein	2	accessory
group_3084	hypothetical protein	2	accessory
group_3085	hypothetical protein	2	accessory
group_3086	chromosome partitioning protein ParA	2	accessory
group_3087	hypothetical protein	2	accessory
group_3088	restriction endonuclease subunit M	2	accessory
group_3089	hypothetical protein	2	accessory
group_3090	hypothetical protein	2	accessory
group_3091	hypothetical protein	2	accessory
group_3092	hypothetical protein	2	accessory
rdgC_2	recombination-associated protein RdgC	2	accessory
group_3094	TrfA family protein	2	accessory
group_3095	hypothetical protein	2	accessory
group_3096	hypothetical protein	2	accessory
group_3097	hypothetical protein	2	accessory
group_3098	hypothetical protein	2	accessory
group_3099	hypothetical protein	2	accessory
group_3100	hypothetical protein	2	accessory
group_3101	hypothetical protein	2	accessory
group_3102	hypothetical protein	2	accessory
group_3103	hypothetical protein	2	accessory
group_3104	hypothetical protein	2	accessory
group_3105	hypothetical protein	2	accessory
group_3106	transcriptional regulator	2	accessory
group_3107	hypothetical protein	2	accessory
group_3108	nucleoid-associated protein	2	accessory
group_3109	hypothetical protein	2	accessory

group_3110	hypothetical protein	2	accessory
group_3111	hypothetical protein	2	accessory
group_3112	hypothetical protein	2	accessory
group_3113	hypothetical protein	2	accessory
group_3114	hypothetical protein	2	accessory
group_3115	hypothetical protein	2	accessory
group_3116	hypothetical protein	2	accessory
group_3117	hypothetical protein	2	accessory
group_3118	hypothetical protein	2	accessory
group_3119	hypothetical protein	2	accessory
group_3120	hypothetical protein	2	accessory
group_3121	hypothetical protein	2	accessory
group_3122	hypothetical protein	2	accessory
group_3123	hypothetical protein	2	accessory
group_3124	hypothetical protein	2	accessory
group_3125	hypothetical protein	2	accessory
group_3126	transcriptional regulator	2	accessory
group_3127	site-specific integrase	2	accessory
group_3128	hypothetical protein	2	accessory
group_3129	hypothetical protein	2	accessory
group_3130	hypothetical protein	2	accessory
group_3131	hypothetical protein	2	accessory
group_3132	hypothetical protein	2	accessory
group_3133	hypothetical protein	2	accessory
group_3134	hypothetical protein	2	accessory
group_3135	hypothetical protein	2	accessory
group_3136	hypothetical protein	2	accessory
group_3137	hypothetical protein	2	accessory
group_3138	hypothetical protein	2	accessory
group_3139	hypothetical protein	2	accessory
group_3140	hypothetical protein	2	accessory
group_3141	hypothetical protein	2	accessory
group_3142	hypothetical protein	2	accessory
group_3143	hypothetical protein	2	accessory
group_3144	hypothetical protein	2	accessory
group_3145	hypothetical protein	2	accessory
group_3146	hypothetical protein	2	accessory
group_3147	hypothetical protein	2	accessory
group_3148	DNA-binding protein	2	accessory

group_3149	hypothetical protein	2	accessory
group_3150	hypothetical protein	2	accessory
group_3151	hypothetical protein	2	accessory
group_3152	hypothetical protein	2	accessory
group_3153	hypothetical protein	2	accessory
group_3154	hypothetical protein	2	accessory
group_3155	hypothetical protein	2	accessory
group_3156	hypothetical protein	2	accessory
group_3157	hypothetical protein	2	accessory
group_3158	hypothetical protein	2	accessory
group_3159	hypothetical protein	2	accessory
group_3160	hypothetical protein	2	accessory
group_3161	hypothetical protein	2	accessory
group_3162	hypothetical protein	2	accessory
group_3163	hypothetical protein	2	accessory
group_3164	hypothetical protein	2	accessory
group_3165	hypothetical protein	2	accessory
group_3166	hypothetical protein	2	accessory
group_3167	hypothetical protein	2	accessory
group_3168	hypothetical protein	2	accessory
group_3169	hypothetical protein	2	accessory
group_3170	hypothetical protein	2	accessory
group_3171	hypothetical protein	2	accessory
group_3172	hypothetical protein	2	accessory
group_3173	hypothetical protein	2	accessory
group_3367	membrane protein	2	accessory
group_3369	hypothetical protein	2	accessory
group_3375	hypothetical protein	2	accessory
group_3392	hypothetical protein	2	accessory
group_3404	hypothetical protein	2	accessory
group_3438	hypothetical protein	2	accessory
group_3439	hypothetical protein	2	accessory
group_3440	hypothetical protein	2	accessory
group_3441	hypothetical protein	2	accessory
group_3472	hypothetical protein	2	accessory
rfe	undecaprenyl-phosphate alpha-N-acetylglucosaminyl 1-phosphate transferase	2	accessory
group_3484	hypothetical protein	2	accessory
group_3498	hypothetical protein	2	accessory
group_3508	hypothetical protein	2	accessory

group_3509	hypothetical protein	2	accessory
group_3521	type I-F CRISPR-associated helicase Cas3	2	accessory
group_3523	hypothetical protein	2	accessory
group_3544	hypothetical protein	2	accessory
group_3595	hypothetical protein	2	accessory
group_3596	UPF0319 protein	2	accessory
group_3597	hypothetical protein	2	accessory
group_3598	outer membrane protein OmpK	2	accessory
gpQ	phage portal protein	2	accessory
group_3784	hypothetical protein	2	accessory
group_484	hypothetical protein	2	accessory
hsdM-2_1	type I restriction-modification system subunit M	1	accessory
group_1176	tRNA-Ala	1	accessory
group_1214	hypothetical protein	1	accessory
group_1334	hypothetical protein	1	accessory
group_1335	hypothetical protein	1	accessory
group_1349	sulfurtransferase	1	accessory
group_1357	hypothetical protein	1	accessory
group_1540	hypothetical protein	1	accessory
group_1560	hypothetical protein	1	accessory
group_1704	hypothetical protein	1	accessory
group_1989	hypothetical protein	1	accessory
group_1991	hypothetical protein	1	accessory
group_2059	hypothetical protein	1	accessory
group_2179	hypothetical protein	1	accessory
group_2180	tRNA-Gly	1	accessory
group_2181	hypothetical protein	1	accessory
group_2182	hypothetical protein	1	accessory
group_2183	hypothetical protein	1	accessory
group_2184	hypothetical protein	1	accessory
group_2189	hypothetical protein	1	accessory
group_2193	hypothetical protein	1	accessory
group_2201	hypothetical protein	1	accessory
group_2206	hypothetical protein	1	accessory
group_2217	cell division protein Fic	1	accessory
group_2218	transcriptional regulator	1	accessory
group_2221	transposase	1	accessory
group_2224	hypothetical protein	1	accessory
group_2225	hypothetical protein	1	accessory

group_2226	hypothetical protein	1	accessory
group_2227	IS30 family transposase	1	accessory
group_2228	hypothetical protein	1	accessory
group_2229	hypothetical protein	1	accessory
group_2230	hypothetical protein	1	accessory
group_2231	gamma-glutamyl-gamma-aminobutyrate hydrolase	1	accessory
group_2232	ferritin	1	accessory
group_2239	hypothetical protein	1	accessory
group_2263	hypothetical protein	1	accessory
group_2264	hypothetical protein	1	accessory
group_2271	hypothetical protein	1	accessory
group_2272	hypothetical protein	1	accessory
group_2273	hypothetical protein	1	accessory
group_2274	hypothetical protein	1	accessory
group_2275	transcriptional regulator	1	accessory
yfeJ	GMP synthase	1	accessory
rhtC	threonine transporter	1	accessory
uppP1	undecaprenyl-diphosphatase 1	1	accessory
group_2280	FAD:protein FMN transferase	1	accessory
group_2282	DNA-binding response regulator	1	accessory
group_2283	Trk system potassium uptake protein	1	accessory
group_2284	potassium transporter TrkA	1	accessory
group_2285	hypothetical protein	1	accessory
group_2286	hypothetical protein	1	accessory
group_2287	hypothetical protein	1	accessory
mpi	invertase	1	accessory
group_2289	peroxiredoxin	1	accessory
group_2290	hypothetical protein	1	accessory
group_2291	hypothetical protein	1	accessory
group_2292	hypothetical protein	1	accessory
group_2293	hypothetical protein	1	accessory
group_2294	hypothetical protein	1	accessory
group_2295	flagellar hook-length control protein FliK	1	accessory
group_2298	hypothetical protein	1	accessory
group_2300	polymerase	1	accessory
group_2301	hexapeptide transferase	1	accessory
group_2302	hypothetical protein	1	accessory
group_2303	oxidoreductase	1	accessory
group_2304	hypothetical protein	1	accessory

group_2305	aminotransferase DegT	1	accessory
group_2306	dTDP-glucose 4,6-dehydratase	1	accessory
rmlA	glucose-1-phosphate thymidylyltransferase	1	accessory
group_2308	hypothetical protein	1	accessory
group_2309	hypothetical protein	1	accessory
wecE	dTDP-4-amino-4,6-dideoxygalactose transaminase	1	accessory
group_2311	polysaccharide biosynthesis protein	1	accessory
group_2312	hypothetical protein	1	accessory
group_2313	hypothetical protein	1	accessory
group_2314	glycosyl transferase	1	accessory
group_2315	LPS biosynthesis protein WbpG	1	accessory
group_2316	imidazole glycerol phosphate synthase subunit HisH 2	1	accessory
group_2317	putative imidazole glycerol phosphate synthase subunit hisF2	1	accessory
fnlA	UDP-N-acetylglucosamine 4,6-dehydratase	1	accessory
fnlB	UDP-2-acetamido-2,6-dideoxy-beta-L-talose 4-dehydrogenase	1	accessory
group_2320	UDP-N-acetyl glucosamine 2-epimerase	1	accessory
group_2321	glycosyltransferase WbuB	1	accessory
group_2322	UDP-glucose 4-epimerase	1	accessory
group_2325	recombinase	1	accessory
group_2326	hypothetical protein	1	accessory
group_2327	hypothetical protein	1	accessory
group_2328	hypothetical protein	1	accessory
group_2329	hypothetical protein	1	accessory
group_2330	hypothetical protein	1	accessory
group_2331	hypothetical protein	1	accessory
group_2332	hypothetical protein	1	accessory
group_2357	hypothetical protein	1	accessory
group_2360	hypothetical protein	1	accessory
group_2361	hypothetical protein	1	accessory
group_2362	hypothetical protein	1	accessory
group_2363	2-hydroxyacid dehydrogenase	1	accessory
group_2364	hypothetical protein	1	accessory
group_2365	DEAD/DEAH box helicase	1	accessory
group_2366	type I restriction-modification system specificity subunit	1	accessory
group_2367	DNA methyltransferase	1	accessory
group_2368	hypothetical protein	1	accessory
group_2369	hypothetical protein	1	accessory
group_2370	DNA methylase	1	accessory
group_2371	hypothetical protein	1	accessory

group_2379	type I-F CRISPR-associated helicase Cas3	1	accessory
group_2384	hypothetical protein	1	accessory
group_2385	hypothetical protein	1	accessory
group_2387	heme biosynthesis protein HemY	1	accessory
group_2388	heme biosynthesis operon protein HemX	1	accessory
group_2389	3',5'-cyclic-nucleotide phosphodiesterase	1	accessory
group_2390	2-haloalkanoic acid dehalogenase	1	accessory
group_2391	hypothetical protein	1	accessory
group_2392	MSHA pilin protein MshD	1	accessory
group_2394	hypothetical protein	1	accessory
group_2395	hypothetical protein	1	accessory
group_2396	type III restriction endonuclease EcoPI subunit R	1	accessory
group_2397	type III restriction endonuclease EcoP15I subunit M	1	accessory
group_2398	hypothetical protein	1	accessory
group_2399	hypothetical protein	1	accessory
group_2400	hypothetical protein	1	accessory
group_2401	hypothetical protein	1	accessory
group_2402	hypothetical protein	1	accessory
group_2406	hypothetical protein	1	accessory
group_2407	hypothetical protein	1	accessory
group_2408	hypothetical protein	1	accessory
group_2409	hypothetical protein	1	accessory
group_2410	hypothetical protein	1	accessory
group_2413	hypothetical protein	1	accessory
group_2415	hypothetical protein	1	accessory
topB_2	DNA topoisomerase 3	1	accessory
group_2417	hypothetical protein	1	accessory
ssb_2	single-stranded DNA-binding protein	1	accessory
group_2419	hypothetical protein	1	accessory
group_2420	hypothetical protein	1	accessory
group_2421	hypothetical protein	1	accessory
group_2422	hypothetical protein	1	accessory
group_2423	hypothetical protein	1	accessory
group_2426	hypothetical protein	1	accessory
group_2427	hypothetical protein	1	accessory
group_2428	hypothetical protein	1	accessory
group_2429	hypothetical protein	1	accessory
group_2430	hypothetical protein	1	accessory
group_2431	hypothetical protein	1	accessory

group_2432	hypothetical protein	1	accessory
group_2433	hypothetical protein	1	accessory
group_2434	hypothetical protein	1	accessory
group_2435	hypothetical protein	1	accessory
group_2436	hypothetical protein	1	accessory
group_2437	hypothetical protein	1	accessory
group_2438	hypothetical protein	1	accessory
group_2439	hypothetical protein	1	accessory
group_2440	hypothetical protein	1	accessory
group_2441	hypothetical protein	1	accessory
group_2442	P-type DNA transfer ATPase VirB11	1	accessory
group_2443	hypothetical protein	1	accessory
group_2444	hypothetical protein	1	accessory
group_2445	hypothetical protein	1	accessory
group_2446	hypothetical protein	1	accessory
group_2447	hypothetical protein	1	accessory
group_2448	hypothetical protein	1	accessory
group_2449	transporter	1	accessory
group_2450	hypothetical protein	1	accessory
group_2451	hypothetical protein	1	accessory
group_2452	hypothetical protein	1	accessory
group_2453	hypothetical protein	1	accessory
group_2454	hypothetical protein	1	accessory
group_2455	hypothetical protein	1	accessory
group_2456	hypothetical protein	1	accessory
group_2457	hypothetical protein	1	accessory
group_2458	hypothetical protein	1	accessory
group_2459	hypothetical protein	1	accessory
group_2460	hypothetical protein	1	accessory
group_2461	hypothetical protein	1	accessory
group_2463	hypothetical protein	1	accessory
group_2472	tryptophan--tRNA ligase	1	accessory
group_2473	hypothetical protein	1	accessory
group_2479	hypothetical protein	1	accessory
group_2481	hypothetical protein	1	accessory
group_2482	hypothetical protein	1	accessory
group_2485	hypothetical protein	1	accessory
group_2488	hypothetical protein	1	accessory
group_2489	hypothetical protein	1	accessory

group_2490	hypothetical protein	1	accessory
group_2492	death-on-curing protein	1	accessory
group_2493	hypothetical protein	1	accessory
group_2500	hypothetical protein	1	accessory
group_2501	hypothetical protein	1	accessory
group_2504	UPF0319 protein	1	accessory
group_2505	porin	1	accessory
group_2506	hypothetical protein	1	accessory
group_2507	hypothetical protein	1	accessory
group_2508	hypothetical protein	1	accessory
group_2509	hypothetical protein	1	accessory
group_2510	hypothetical protein	1	accessory
group_2511	hypothetical protein	1	accessory
group_2512	hypothetical protein	1	accessory
group_2513	hypothetical protein	1	accessory
group_2514	prepilin-type N-terminal cleavage/methylation domain-containing protein	1	accessory
group_2515	cytosine-specific methyltransferase	1	accessory
group_2517	hypothetical protein	1	accessory
group_2518	hypothetical protein	1	accessory
group_2519	hypothetical protein	1	accessory
group_2520	hypothetical protein	1	accessory
group_2521	hypothetical protein	1	accessory
rulB_1	DNA polymerase V subunit UmuC	1	accessory
umuD_1	DNA polymerase V	1	accessory
group_2524	hypothetical protein	1	accessory
group_2525	hypothetical protein	1	accessory
group_2526	hypothetical protein	1	accessory
group_2527	chemotaxis protein	1	accessory
cheV	chemotaxis protein CheW	1	accessory
group_2529	hypothetical protein	1	accessory
group_2530	methyl-accepting chemotaxis protein	1	accessory
group_2531	hypothetical protein	1	accessory
group_2532	transposase	1	accessory
group_2533	IS110 family transposase	1	accessory
group_2534	hypothetical protein	1	accessory
group_2535	hypothetical protein	1	accessory
group_2536	hypothetical protein	1	accessory
group_2537	hypothetical protein	1	accessory
group_2538	hypothetical protein	1	accessory

group_2539	hypothetical protein	1	accessory
group_2540	DNA helicase	1	accessory
group_2541	hypothetical protein	1	accessory
traH_1	conjugal transfer protein TraH	1	accessory
group_2543	hypothetical protein	1	accessory
group_2544	hypothetical protein	1	accessory
group_2545	hypothetical protein	1	accessory
group_2546	hypothetical protein	1	accessory
group_2547	lytic transglycosylase	1	accessory
group_2548	hypothetical protein	1	accessory
group_2549	hypothetical protein	1	accessory
group_2550	hypothetical protein	1	accessory
group_2551	hypothetical protein	1	accessory
group_2552	hypothetical protein	1	accessory
group_2553	hypothetical protein	1	accessory
group_2554	hypothetical protein	1	accessory
group_2555	hypothetical protein	1	accessory
group_2556	hypothetical protein	1	accessory
group_2557	hypothetical protein	1	accessory
group_2558	hypothetical protein	1	accessory
group_2559	hypothetical protein	1	accessory
group_2560	hypothetical protein	1	accessory
group_2561	hypothetical protein	1	accessory
group_2562	peptidase	1	accessory
group_2563	hypothetical protein	1	accessory
group_2564	hypothetical protein	1	accessory
group_2565	endonuclease	1	accessory
group_2566	hypothetical protein	1	accessory
group_2567	hypothetical protein	1	accessory
group_2568	hypothetical protein	1	accessory
group_2569	hypothetical protein	1	accessory
group_2570	hypothetical protein	1	accessory
group_2571	hypothetical protein	1	accessory
group_2572	hypothetical protein	1	accessory
group_2573	hypothetical protein	1	accessory
group_2574	hypothetical protein	1	accessory
group_2575	hypothetical protein	1	accessory
group_2576	hypothetical protein	1	accessory
group_2577	hypothetical protein	1	accessory

group_2578	hypothetical protein	1	accessory
group_2579	hypothetical protein	1	accessory
group_2580	hypothetical protein	1	accessory
group_2581	hypothetical protein	1	accessory
group_2582	hypothetical protein	1	accessory
group_2583	hypothetical protein	1	accessory
group_2584	hypothetical protein	1	accessory
group_2585	hypothetical protein	1	accessory
group_2586	hypothetical protein	1	accessory
group_2587	hypothetical protein	1	accessory
group_2588	hypothetical protein	1	accessory
group_2589	hypothetical protein	1	accessory
group_2590	hypothetical protein	1	accessory
group_2591	hypothetical protein	1	accessory
group_2592	hypothetical protein	1	accessory
group_2593	hypothetical protein	1	accessory
group_2594	hypothetical protein	1	accessory
group_2595	hypothetical protein	1	accessory
group_2596	hypothetical protein	1	accessory
group_2597	monooxygenase	1	accessory
group_2598	transcriptional regulator	1	accessory
group_2599	hypothetical protein	1	accessory
group_2600	hypothetical protein	1	accessory
group_2601	hypothetical protein	1	accessory
group_2602	hypothetical protein	1	accessory
group_2603	hypothetical protein	1	accessory
group_2604	phage integrase	1	accessory
group_2605	transcriptional regulator	1	accessory
group_2606	hypothetical protein	1	accessory
group_2607	restriction modification system DNA specificity domain-containing protein	1	accessory
prrC	anticodon nuclease	1	accessory
group_2609	DEAD/DEAH box helicase	1	accessory
group_2610	hypothetical protein	1	accessory
group_2611	chromosome partitioning protein ParA	1	accessory
group_2612	hypothetical protein	1	accessory
group_2613	hypothetical protein	1	accessory
group_2614	hypothetical protein	1	accessory
group_2615	hypothetical protein	1	accessory
group_2616	hypothetical protein	1	accessory

group_2617	hypothetical protein	1	accessory
group_2618	hypothetical protein	1	accessory
group_2619	hypothetical protein	1	accessory
group_2620	hypothetical protein	1	accessory
group_2621	hypothetical protein	1	accessory
group_2622	hypothetical protein	1	accessory
group_2623	hypothetical protein	1	accessory
group_2624	hypothetical protein	1	accessory
group_2625	IS5/IS1182 family transposase	1	accessory
group_2626	hypothetical protein	1	accessory
group_2627	hypothetical protein	1	accessory
group_2628	hypothetical protein	1	accessory
group_2629	hypothetical protein	1	accessory
group_2630	hypothetical protein	1	accessory
group_2631	DNA topoisomerase III	1	accessory
group_2632	hypothetical protein	1	accessory
group_2633	hypothetical protein	1	accessory
group_2634	hypothetical protein	1	accessory
group_2635	hypothetical protein	1	accessory
group_2636	conjugative coupling factor TraD, PFGI-1 class	1	accessory
group_2637	hypothetical protein	1	accessory
group_2638	hypothetical protein	1	accessory
group_2639	hypothetical protein	1	accessory
group_2640	hypothetical protein	1	accessory
group_2641	hypothetical protein	1	accessory
group_2642	hypothetical protein	1	accessory
group_2643	hypothetical protein	1	accessory
group_2644	hypothetical protein	1	accessory
group_2645	hypothetical protein	1	accessory
group_2646	hypothetical protein	1	accessory
group_2647	hypothetical protein	1	accessory
group_2648	hypothetical protein	1	accessory
group_2649	hypothetical protein	1	accessory
group_2650	plasmid transfer protein	1	accessory
group_2651	hypothetical protein	1	accessory
trhF	signal peptidase I	1	accessory
group_2653	hypothetical protein	1	accessory
group_2654	hypothetical protein	1	accessory
group_2655	hypothetical protein	1	accessory

group_2656	hypothetical protein	1	accessory
group_2657	hypothetical protein	1	accessory
group_2658	hypothetical protein	1	accessory
group_2659	hypothetical protein	1	accessory
group_2660	hypothetical protein	1	accessory
group_2661	hypothetical protein	1	accessory
group_2662	hypothetical protein	1	accessory
group_2663	hypothetical protein	1	accessory
group_2664	cobalamin synthase	1	accessory
group_2665	hypothetical protein	1	accessory
group_2666	hypothetical protein	1	accessory
group_2667	hypothetical protein	1	accessory
group_2668	hypothetical protein	1	accessory
group_2669	hypothetical protein	1	accessory
group_2670	hypothetical protein	1	accessory
group_2671	hypothetical protein	1	accessory
group_2672	hypothetical protein	1	accessory
group_2673	hypothetical protein	1	accessory
group_2674	hypothetical protein	1	accessory
group_2675	hypothetical protein	1	accessory
group_2676	(2Fe-2S) ferredoxin	1	accessory
group_2677	hypothetical protein	1	accessory
group_2678	hypothetical protein	1	accessory
group_2679	hypothetical protein	1	accessory
group_2680	hypothetical protein	1	accessory
group_2681	hypothetical protein	1	accessory
group_2682	hypothetical protein	1	accessory
tusA_1	sulfurtransferase TusA	1	accessory
group_2684	hypothetical protein	1	accessory
group_2685	hypothetical protein	1	accessory
group_2686	hypothetical protein	1	accessory
group_2687	hypothetical protein	1	accessory
group_2688	hypothetical protein	1	accessory
group_2689	hypothetical protein	1	accessory
group_2690	hypothetical protein	1	accessory
group_2691	hypothetical protein	1	accessory
group_2692	hypothetical protein	1	accessory
group_2693	hypothetical protein	1	accessory
group_2694	hypothetical protein	1	accessory

group_2695	hypothetical protein	1	accessory
group_2696	hypothetical protein	1	accessory
group_2697	hypothetical protein	1	accessory
group_2698	hypothetical protein	1	accessory
group_2699	hypothetical protein	1	accessory
group_2700	hypothetical protein	1	accessory
group_2701	hypothetical protein	1	accessory
group_2702	hypothetical protein	1	accessory
group_2703	hypothetical protein	1	accessory
group_2704	hypothetical protein	1	accessory
group_2705	hypothetical protein	1	accessory
group_2706	hypothetical protein	1	accessory
group_2707	hypothetical protein	1	accessory
group_2708	hypothetical protein	1	accessory
group_2709	hypothetical protein	1	accessory
group_2710	integrase	1	accessory
group_2711	hypothetical protein	1	accessory
group_2712	hypothetical protein	1	accessory
group_2714	hypothetical protein	1	accessory
group_2715	hypothetical protein	1	accessory
group_2716	UPF0213 protein	1	accessory
group_2718	hypothetical protein	1	accessory
group_2720	UDP-glucose 6-dehydrogenase	1	accessory
group_2721	NAD-dependent epimerase	1	accessory
group_2722	glycosyl transferase	1	accessory
group_2723	glycosyl transferase	1	accessory
group_2724	hypothetical protein	1	accessory
group_2725	polysaccharide biosynthesis protein	1	accessory
group_2726	glycosyl transferase	1	accessory
group_2727	glycosyl transferase family 2	1	accessory
group_2728	hypothetical protein	1	accessory
group_2729	hypothetical protein	1	accessory
group_2730	sugar ABC transporter ATP-binding protein	1	accessory
group_2731	transport permease protein	1	accessory
rfbC-1	dTDP-4-dehydrorhamnose 3,5-epimerase	1	accessory
rfbD	NAD(P)-dependent oxidoreductase	1	accessory
group_2734	hexapeptide transferase	1	accessory
group_2736	cell division protein ZipA	1	accessory
intA_1	integrase	1	accessory

group_2738	hypothetical protein	1	accessory
group_2739	hypothetical protein	1	accessory
group_2740	hypothetical protein	1	accessory
group_2741	type I restriction-modification protein subunit M	1	accessory
group_2742	restriction endonuclease subunit S	1	accessory
group_2743	type I deoxyribonuclease HsdR	1	accessory
group_2744	hypothetical protein	1	accessory
group_2748	hypothetical protein	1	accessory
group_2749	hypothetical protein	1	accessory
soxR	redox-sensitive transcriptional activator SoxR	1	accessory
group_2751	hypothetical protein	1	accessory
group_2752	hypothetical protein	1	accessory
group_2753	hypothetical protein	1	accessory
group_2754	hypothetical protein	1	accessory
group_2755	hypothetical protein	1	accessory
group_2756	hypothetical protein	1	accessory
group_2757	hypothetical protein	1	accessory
group_2758	serine recombinase	1	accessory
group_2759	MSHA biogenesis protein MshF	1	accessory
group_2760	hypothetical protein	1	accessory
group_2761	MSHA pilin protein MshC	1	accessory
group_2764	long-chain fatty acid transporter	1	accessory
group_2766	integrase	1	accessory
group_2767	hypothetical protein	1	accessory
group_2768	hypothetical protein	1	accessory
group_2769	hypothetical protein	1	accessory
intA_2	integrase	1	accessory
group_2771	undecaprenyl-diphosphatase 1	1	accessory
group_2772	hypothetical protein	1	accessory
group_2773	type I restriction-modification protein subunit S	1	accessory
group_2774	hypothetical protein	1	accessory
group_2775	hypothetical protein	1	accessory
group_2776	DUF1016 domain-containing protein	1	accessory
group_2777	type I restriction-modification system, restriction (R) subunit	1	accessory
group_2778	hypothetical protein	1	accessory
group_2779	hypothetical protein	1	accessory
group_2780	hypothetical protein	1	accessory
group_2781	hypothetical protein	1	accessory
group_2782	hypothetical protein	1	accessory

group_2783	DNA-binding protein	1	accessory
group_2784	hypothetical protein	1	accessory
group_2785	serine/threonine protein phosphatase	1	accessory
group_2786	hypothetical protein	1	accessory
group_2787	hypothetical protein	1	accessory
group_2788	transposase	1	accessory
group_2789	hypothetical protein	1	accessory
group_2790	hypothetical protein	1	accessory
group_2791	IS5 family transposase	1	accessory
group_2792	hypothetical protein	1	accessory
group_2793	hypothetical protein	1	accessory
creA	catabolite regulation protein CreA	1	accessory
group_2796	hypothetical protein	1	accessory
group_2797	hypothetical protein	1	accessory
group_2798	hypothetical protein	1	accessory
group_2799	hypothetical protein	1	accessory
group_2800	hypothetical protein	1	accessory
group_2801	hypothetical protein	1	accessory
group_2802	hypothetical protein	1	accessory
group_2803	hypothetical protein	1	accessory
group_2804	hypothetical protein	1	accessory
group_2805	hypothetical protein	1	accessory
group_2806	hypothetical protein	1	accessory
group_2807	hypothetical protein	1	accessory
group_2808	hypothetical protein	1	accessory
group_2809	hypothetical protein	1	accessory
group_2810	hypothetical protein	1	accessory
group_2811	hypothetical protein	1	accessory
group_2812	hypothetical protein	1	accessory
group_2813	hypothetical protein	1	accessory
group_2814	hypothetical protein	1	accessory
group_2815	hypothetical protein	1	accessory
group_2816	hypothetical protein	1	accessory
hisK	putative histidinol-phosphatase	1	accessory
group_2818	hypothetical protein	1	accessory
nrdG_1	anaerobic ribonucleoside-triphosphate reductase-activating protein	1	accessory
group_2820	chaperone	1	accessory
group_2821	hypothetical protein	1	accessory
group_2822	hypothetical protein	1	accessory

group_2823	hypothetical protein	1	accessory
group_2824	hypothetical protein	1	accessory
group_2825	hypothetical protein	1	accessory
group_2826	WYL domain-containing protein	1	accessory
group_2827	hypothetical protein	1	accessory
group_2828	hypothetical protein	1	accessory
group_2829	hypothetical protein	1	accessory
group_2830	hypothetical protein	1	accessory
group_2831	hypothetical protein	1	accessory
group_2832	hypothetical protein	1	accessory
group_2833	hypothetical protein	1	accessory
narL	nitrate/nitrite response regulator protein NarL	1	accessory
group_2835	putative iron-sulfur cluster repair protein	1	accessory
group_2836	hypothetical protein	1	accessory
tehB	tellurite resistance protein B	1	accessory
norW	nitric oxide reductase FlRd-NAD(+) reductase	1	accessory
norV	anaerobic nitric oxide reductase flavorubredoxin	1	accessory
norR	anaerobic nitric oxide reductase transcription regulator NorR	1	accessory
group_2841	azurin	1	accessory
group_2842	hypothetical protein	1	accessory
group_2843	cupin	1	accessory
group_2844	SAM-dependent methyltransferase	1	accessory
group_2845	hypothetical protein	1	accessory
group_2846	hypothetical protein	1	accessory
group_2847	cell filamentation protein Fic	1	accessory
group_2848	hypothetical protein	1	accessory
group_2849	WYL domain-containing protein	1	accessory
group_2850	IS1182 family transposase	1	accessory
group_2852	hypothetical protein	1	accessory
group_2853	hypothetical protein	1	accessory
group_2854	hypothetical protein	1	accessory
group_2855	hypothetical protein	1	accessory
group_2856	hypothetical protein	1	accessory
group_2857	hypothetical protein	1	accessory
group_2858	hypothetical protein	1	accessory
group_2859	hypothetical protein	1	accessory
group_2860	hypothetical protein	1	accessory
group_2861	hypothetical protein	1	accessory
group_2862	hypothetical protein	1	accessory

group_2863	hypothetical protein	1	accessory
group_2865	hypothetical protein	1	accessory
group_2866	hypothetical protein	1	accessory
group_2868	hypothetical protein	1	accessory
group_2869	acetyltransferase	1	accessory
group_2870	hypothetical protein	1	accessory
group_2871	hypothetical protein	1	accessory
glgC	glucose-1-phosphate adenylyltransferase	1	accessory
group_2873	hypothetical protein	1	accessory
group_2875	hypothetical protein	1	accessory
group_2876	hypothetical protein	1	accessory
group_2877	protein killer protein	1	accessory
group_2878	transcriptional regulator	1	accessory
yegD	molecular chaperone	1	accessory
group_2881	hypothetical protein	1	accessory
group_2882	hypothetical protein	1	accessory
group_2883	hypothetical protein	1	accessory
group_2884	hypothetical protein	1	accessory
group_2885	SAM-dependent methyltransferase	1	accessory
group_2886	transcriptional regulator	1	accessory
group_2887	hypothetical protein	1	accessory
group_2888	transposase	1	accessory
group_2889	transposase	1	accessory
group_2890	transposase	1	accessory
group_2891	hypothetical protein	1	accessory
group_3351	hypothetical protein	1	accessory
group_3477	prepilin-type N-terminal cleavage/methylation domain-containing protein	1	accessory
group_3478	hypothetical protein	1	accessory
group_3479	hypothetical protein	1	accessory
group_3480	hypothetical protein	1	accessory
group_3481	hypothetical protein	1	accessory
group_3482	hypothetical protein	1	accessory
group_3483	hypothetical protein	1	accessory
group_3485	peptide methionine sulfoxide reductase MsrA/MsrB	1	accessory
group_3486	hypothetical protein	1	accessory
group_3487	hypothetical protein	1	accessory
group_3488	hypothetical protein	1	accessory
group_3489	transposase	1	accessory
group_3490	transposase	1	accessory

group_3491	hypothetical protein	1	accessory
group_3492	N-acetyltransferase GCN5	1	accessory
group_3493	hypothetical protein	1	accessory
group_3494	hypothetical protein	1	accessory
group_3495	outer membrane protein U	1	accessory
group_3496	hypothetical protein	1	accessory
group_3497	hypothetical protein	1	accessory
group_3499	hypothetical protein	1	accessory
group_3500	hypothetical protein	1	accessory
wlbA	oxidoreductase	1	accessory
wbpD	N-acetyltransferase	1	accessory
degT	aminotransferase DegT	1	accessory
group_3504	acetyltransferase	1	accessory
group_3505	polysaccharide biosynthesis protein	1	accessory
group_3506	asparagine synthetase B	1	accessory
group_3507	hypothetical protein	1	accessory
group_3510	hypothetical protein	1	accessory
capI	glycosyltransferase	1	accessory
group_3512	imidazole glycerol phosphate synthase subunit HisH 2	1	accessory
group_3513	putative imidazole glycerol phosphate synthase subunit hisF2	1	accessory
group_3514	LPS biosynthesis protein WbpG	1	accessory
group_3515	hypothetical protein	1	accessory
wbpH	glycosyltransferase WbpH	1	accessory
wbpI	UDP-2,3-diacetamido-2,3-dideoxy-D-glucuronate 2-epimerase	1	accessory
group_3518	glycosyltransferase WbuB	1	accessory
group_3519	UDP-glucose 4-epimerase	1	accessory
group_3520	WYL domain-containing protein	1	accessory
group_3522	porin	1	accessory
group_3525	porin	1	accessory
group_3526	transposase	1	accessory
group_3527	deoxycytidylate deaminase	1	accessory
group_3528	hypothetical protein	1	accessory
group_3529	hypothetical protein	1	accessory
vspR	transcriptional regulator VspR	1	accessory
group_3531	ATPase AAA	1	accessory
group_3532	hypothetical protein	1	accessory
group_3533	hypothetical protein	1	accessory
group_3534	hypothetical protein	1	accessory
ymfE	hypothetical protein	1	accessory

ymfD	hypothetical protein	1	accessory
group_3537	transposase	1	accessory
group_3538	transposase	1	accessory
group_3539	hypothetical protein	1	accessory
group_3540	hypothetical protein	1	accessory
group_3541	hypothetical protein	1	accessory
group_3542	hypothetical protein	1	accessory
group_3543	hypothetical protein	1	accessory
group_3546	hypothetical protein	1	accessory
group_3547	hypothetical protein	1	accessory
group_3548	hypothetical protein	1	accessory
cspG	cold-shock protein	1	accessory
acnA	aconitate hydratase	1	accessory
group_3551	Na(+)/H(+) antiporter NhaA	1	accessory
group_3552	protein HflC	1	accessory
group_3553	HflK protein	1	accessory
group_3554	cold-shock protein	1	accessory
group_3555	hypothetical protein	1	accessory
group_3556	hypothetical protein	1	accessory
group_3557	hypothetical protein	1	accessory
group_3558	hypothetical protein	1	accessory
group_3559	hypothetical protein	1	accessory
group_3560	cold-shock protein	1	accessory
group_3561	chromosome segregation ATPase	1	accessory
group_3562	phage integrase	1	accessory
group_3563	hypothetical protein	1	accessory
group_3564	ATP-dependent helicase	1	accessory
group_3565	hypothetical protein	1	accessory
group_3566	hypothetical protein	1	accessory
group_3567	hypothetical protein	1	accessory
group_3568	hypothetical protein	1	accessory
group_3569	hypothetical protein	1	accessory
group_3570	hypothetical protein	1	accessory
group_3571	hypothetical protein	1	accessory
group_3572	hypothetical protein	1	accessory
group_3573	hypothetical protein	1	accessory
group_3574	hypothetical protein	1	accessory
group_3575	hypothetical protein	1	accessory
group_3576	hypothetical protein	1	accessory

group_3577	hypothetical protein	1	accessory
traD	conjugal transfer protein TraD	1	accessory
trwC	conjugative relaxase	1	accessory
group_3580	hypothetical protein	1	accessory
group_3581	hypothetical protein	1	accessory
group_3582	hypothetical protein	1	accessory
group_3583	hypothetical protein	1	accessory
group_3584	hypothetical protein	1	accessory
group_3585	hypothetical protein	1	accessory
group_3586	hypothetical protein	1	accessory
group_3587	hypothetical protein	1	accessory
group_3588	hypothetical protein	1	accessory
group_3589	hypothetical protein	1	accessory
group_3590	hypothetical protein	1	accessory
group_3591	hypothetical protein	1	accessory
group_3592	integrase	1	accessory
group_3593	hypothetical protein	1	accessory
group_3594	hypothetical protein	1	accessory
group_3599	hypothetical protein	1	accessory
group_3600	prepilin-type N-terminal cleavage/methylation domain-containing protein	1	accessory
group_3601	hypothetical protein	1	accessory
group_3602	hypothetical protein	1	accessory
group_3603	hypothetical protein	1	accessory
group_3604	hypothetical protein	1	accessory
group_3605	hypothetical protein	1	accessory
group_3606	hypothetical protein	1	accessory
group_3607	hypothetical protein	1	accessory
group_3608	hypothetical protein	1	accessory
group_3609	hypothetical protein	1	accessory
group_3610	hypothetical protein	1	accessory
group_3611	hypothetical protein	1	accessory
group_3612	hypothetical protein	1	accessory
group_3613	membrane protein	1	accessory
group_3614	hypothetical protein	1	accessory
group_3615	hypothetical protein	1	accessory
group_3616	hypothetical protein	1	accessory
group_3617	hypothetical protein	1	accessory
group_3618	hypothetical protein	1	accessory
group_3619	hypothetical protein	1	accessory

group_3620	hypothetical protein	1	accessory
group_3621	hypothetical protein	1	accessory
group_3622	hypothetical protein	1	accessory
group_3623	hypothetical protein	1	accessory
group_3624	hypothetical protein	1	accessory
group_3625	hypothetical protein	1	accessory
group_3626	hypothetical protein	1	accessory
group_3627	hypothetical protein	1	accessory
group_3628	glycosyl transferase	1	accessory
group_3629	acyltransferase	1	accessory
group_3630	hypothetical protein	1	accessory
group_3631	glycosyl transferase	1	accessory
group_3632	Lsg locus putative protein 4	1	accessory
group_3633	hypothetical protein	1	accessory
group_3634	hypothetical protein	1	accessory
group_3636	(R)-specific enoyl-CoA hydratase	1	accessory
perB	GDP-perosamine N-acetyltransferase	1	accessory
group_3638	sugar transferase	1	accessory
group_3639	glycosyltransferase WbuB	1	accessory
group_3640	hypothetical protein	1	accessory
group_3641	hypothetical protein	1	accessory
group_3642	hypothetical protein	1	accessory
group_3643	hypothetical protein	1	accessory
group_3644	hypothetical protein	1	accessory
group_3645	asparagine synthase	1	accessory
group_3646	polysaccharide biosynthesis protein	1	accessory
group_3647	acetyltransferase	1	accessory
group_3648	aminotransferase DegT	1	accessory
group_3649	N-acetyltransferase	1	accessory
group_3650	UDP-N-acetyl-d-glucosamine 6-dehydrogenase WbpA	1	accessory
group_3651	oxidoreductase	1	accessory
fepE	LPS O-antigen length regulator	1	accessory
group_3653	hypothetical protein	1	accessory
group_3654	hypothetical protein	1	accessory
group_3655	hypothetical protein	1	accessory
group_3656	hypothetical protein	1	accessory
group_3657	hypothetical protein	1	accessory
group_3658	5'-nucleotidase	1	accessory
group_3659	hypothetical protein	1	accessory

group_3660	hypothetical protein	1	accessory
group_3661	hypothetical protein	1	accessory
group_3662	DNA methyltransferase	1	accessory
group_3663	restriction endonuclease subunit R	1	accessory
group_3664	hypothetical protein	1	accessory
group_3665	hypothetical protein	1	accessory
group_3666	type I-F CRISPR-associated protein Csy3	1	accessory
group_3667	type I-F CRISPR-associated endoribonuclease Cas6/Csy4	1	accessory
group_3668	transcriptional regulator	1	accessory
group_3669	recombinase	1	accessory
group_3670	hypothetical protein	1	accessory
group_3671	hypothetical protein	1	accessory
group_3672	hypothetical protein	1	accessory
group_3673	hypothetical protein	1	accessory
group_3674	hypothetical protein	1	accessory
group_3675	hypothetical protein	1	accessory
group_3676	hypothetical protein	1	accessory
group_3678	hypothetical protein	1	accessory
group_3679	hypothetical protein	1	accessory
group_3680	transposase	1	accessory
group_3681	diguanylate cyclase	1	accessory
group_3682	hypothetical protein	1	accessory
group_3683	hypothetical protein	1	accessory
tnpR	resolvase	1	accessory
group_3685	hypothetical protein	1	accessory
group_3686	hypothetical protein	1	accessory
group_3687	SAM-dependent methyltransferase	1	accessory
group_3688	hypothetical protein	1	accessory
group_3689	hypothetical protein	1	accessory
group_3690	hypothetical protein	1	accessory
group_3691	membrane protein	1	accessory
group_3692	long-chain fatty acid transporter	1	accessory
group_3693	hypothetical protein	1	accessory
group_3694	hypothetical protein	1	accessory
group_3695	hypothetical protein	1	accessory
group_3696	hypothetical protein	1	accessory
group_3697	hypothetical protein	1	accessory
group_3698	hypothetical protein	1	accessory
group_3699	hypothetical protein	1	accessory

group_3700	hypothetical protein	1	accessory
group_3701	hypothetical protein	1	accessory
group_3702	hypothetical protein	1	accessory
group_3703	hypothetical protein	1	accessory
group_3704	hypothetical protein	1	accessory
trhN	conjugal transfer protein TraN	1	accessory
group_3706	hypothetical protein	1	accessory
group_3707	hypothetical protein	1	accessory
group_3708	hypothetical protein	1	accessory
group_3709	hypothetical protein	1	accessory
group_3710	hypothetical protein	1	accessory
group_3711	hypothetical protein	1	accessory
group_3712	hypothetical protein	1	accessory
group_3713	hypothetical protein	1	accessory
group_3714	hypothetical protein	1	accessory
group_3715	hypothetical protein	1	accessory
group_3716	hypothetical protein	1	accessory
group_3717	hypothetical protein	1	accessory
group_3718	hypothetical protein	1	accessory
group_3719	hypothetical protein	1	accessory
group_3720	hypothetical protein	1	accessory
group_3721	hypothetical protein	1	accessory
group_3722	hypothetical protein	1	accessory
group_3723	hypothetical protein	1	accessory
group_3724	hypothetical protein	1	accessory
group_3725	hypothetical protein	1	accessory
group_3726	hypothetical protein	1	accessory
group_3727	hypothetical protein	1	accessory
group_3728	hypothetical protein	1	accessory
group_3729	hypothetical protein	1	accessory
group_3730	hypothetical protein	1	accessory
group_3731	hypothetical protein	1	accessory
group_3735	hypothetical protein	1	accessory
group_3736	hypothetical protein	1	accessory
group_3737	hypothetical protein	1	accessory
group_3739	hypothetical protein	1	accessory
group_3740	hypothetical protein	1	accessory
group_3741	hypothetical protein	1	accessory
group_3742	hypothetical protein	1	accessory

group_3743	porin	1	accessory
group_3744	UPF0319 protein	1	accessory
group_3745	pilus assembly protein PilA	1	accessory
group_3746	putative HTH-type transcriptional regulator	1	accessory
group_3747	hypothetical protein	1	accessory
group_3748	integrase	1	accessory
group_3749	type I restriction-modification system subunit M	1	accessory
group_3750	anticodon nuclease	1	accessory
group_3751	type I site-specific deoxyribonuclease specificity subunit	1	accessory
group_3752	DEAD/DEAH box helicase	1	accessory
group_3753	hypothetical protein	1	accessory
group_3754	transcriptional regulator	1	accessory
group_3755	UPF0758 protein	1	accessory
group_3756	hypothetical protein	1	accessory
group_3757	hypothetical protein	1	accessory
group_3758	hypothetical protein	1	accessory
group_3759	hypothetical protein	1	accessory
group_3760	lipoprotein	1	accessory
group_3761	hypothetical protein	1	accessory
group_3762	hypothetical protein	1	accessory
group_3763	ribonuclease H	1	accessory
group_3764	hypothetical protein	1	accessory
group_3765	hypothetical protein	1	accessory
group_3766	hypothetical protein	1	accessory
group_3767	hypothetical protein	1	accessory
group_3768	phosphoglucosamine mutase	1	accessory
group_3769	hypothetical protein	1	accessory
group_3770	hypothetical protein	1	accessory
group_3771	hypothetical protein	1	accessory
group_3772	hypothetical protein	1	accessory
group_3773	hypothetical protein	1	accessory
group_3774	hypothetical protein	1	accessory
group_3775	P-type conjugative transfer protein TrbL	1	accessory
group_3776	hypothetical protein	1	accessory
group_3777	conjugal transfer protein TrbJ	1	accessory
group_3778	stabilization protein	1	accessory
group_3779	hypothetical protein	1	accessory
group_3780	hypothetical protein	1	accessory
group_3781	transcriptional regulator	1	accessory

group_3782	phage-related integrase	1	accessory
group_3783	hypothetical protein	1	accessory
group_3785	hypothetical protein	1	accessory
group_3786	hypothetical protein	1	accessory
group_3787	hypothetical protein	1	accessory
group_3788	hypothetical protein	1	accessory
mazF	endoribonuclease MazF	1	accessory
group_3790	hypothetical protein	1	accessory
group_3791	toxin HipA	1	accessory
group_3792	hypothetical protein	1	accessory
group_3793	hypothetical protein	1	accessory
group_3794	membrane protein	1	accessory
group_3795	hypothetical protein	1	accessory
group_3796	hypothetical protein	1	accessory
group_3797	hypothetical protein	1	accessory
group_3798	hypothetical protein	1	accessory
group_3799	toxin RelE	1	accessory
group_3800	hypothetical protein	1	accessory
group_3801	hypothetical protein	1	accessory
group_3802	DEAD/DEAH box helicase	1	accessory
group_3803	DNA-binding protein	1	accessory
group_3804	toxin RelE	1	accessory
group_3805	hypothetical protein	1	accessory
group_3806	integrase	1	accessory
group_3807	Lex2B	1	accessory
group_3808	hypothetical protein	1	accessory
group_3809	hypothetical protein	1	accessory
group_3810	glucose-1-phosphate thymidylyltransferase	1	accessory
group_3811	dTDP-6-deoxy-3,4-keto-hexulose isomerase	1	accessory
group_3812	transferase	1	accessory
phaJ	(R)-specific enoyl-CoA hydratase	1	accessory
group_3814	aminotransferase	1	accessory
group_3815	dTDP-4-dehydrorhamnose 3,5-epimerase	1	accessory
group_3816	dTDP-glucose 4,6-dehydratase	1	accessory
wzxE	lipid III flippase	1	accessory
group_3818	hypothetical protein	1	accessory
group_3819	hypothetical protein	1	accessory
group_3820	hypothetical protein	1	accessory
group_3821	hypothetical protein	1	accessory

exoZ	acyltransferase	1	accessory
group_3823	epimerase	1	accessory
group_3824	undecaprenyl-phosphate beta-N-acetyl-D-fucosaminephosphotransferase	1	accessory
group_3825	glycosyl transferase	1	accessory
group_3826	hypothetical protein	1	accessory
group_3827	LPS-assembly lipoprotein LptE	1	accessory
group_3828	membrane protein	1	accessory
group_3829	restriction modification system DNA specificity domain-containing protein	1	accessory
group_3830	iron(III) ABC transporter substrate-binding protein	1	accessory
group_3831	iron(III) ABC transporter permease	1	accessory
group_3832	iron(III) ABC transporter permease	1	accessory
group_3833	iron(III) ABC transporter ATP-binding protein	1	accessory
group_3834	AraC family transcriptional regulator	1	accessory
group_3835	hypothetical protein	1	accessory
group_3836	hypothetical protein	1	accessory
group_3837	long-chain fatty acid transporter	1	accessory
group_3838	hypothetical protein	1	accessory
group_3839	hypothetical protein	1	accessory
group_3840	hypothetical protein	1	accessory
group_3841	DNA methyltransferase	1	accessory
group_3842	type I restriction-modification system endonuclease	1	accessory
group_3843	hypothetical protein	1	accessory
group_3844	hypothetical protein	1	accessory
group_3845	hypothetical protein	1	accessory
group_3846	transcriptional regulator	1	accessory
group_3847	PTS sugar transporter subunit IIABC	1	accessory
group_3848	putative N-acetylmannosamine-6-phosphate 2-epimerase	1	accessory
group_3849	N-acetylglucosamine-6-phosphate deacetylase	1	accessory
group_3850	ribonuclease H	1	accessory
group_3851	hypothetical protein	1	accessory
group_3852	hypothetical protein	1	accessory
group_3854	hypothetical protein	1	accessory
group_3855	LPS-assembly protein LptD	1	accessory
group_3856	hypothetical protein	1	accessory
group_3857	hypothetical protein	1	accessory
group_3858	transcriptional regulator	1	accessory
group_3859	hypothetical protein	1	accessory
group_3861	DNA polymerase V	1	accessory
group_3862	DNA polymerase V subunit UmuC	1	accessory

group_3863	hypothetical protein	1	accessory
group_3864	hypothetical protein	1	accessory
group_3865	hypothetical protein	1	accessory
group_3866	hypothetical protein	1	accessory
group_3867	hypothetical protein	1	accessory
group_3868	hypothetical protein	1	accessory
group_3869	hypothetical protein	1	accessory
group_3870	MSHA pilin protein MshD	1	accessory
group_3871	hypothetical protein	1	accessory
group_3872	hypothetical protein	1	accessory
group_3873	integrase	1	accessory
group_3874	hypothetical protein	1	accessory
group_3875	antitoxin	1	accessory
group_3876	N-acetyltransferase	1	accessory
group_3877	hypothetical protein	1	accessory
mipA	scaffold protein	1	accessory
group_3879	hypothetical protein	1	accessory
group_3880	hypothetical protein	1	accessory
group_3881	hypothetical protein	1	accessory
group_3882	hypothetical protein	1	accessory
group_3883	acylphosphatase	1	accessory
group_3884	hypothetical protein	1	accessory
group_3885	hypothetical protein	1	accessory
group_3886	hypothetical protein	1	accessory
group_3887	hypothetical protein	1	accessory
group_3888	hypothetical protein	1	accessory
group_3889	hypothetical protein	1	accessory
group_3890	hypothetical protein	1	accessory
group_3891	hypothetical protein	1	accessory
group_3892	hypothetical protein	1	accessory
group_3893	transcriptional regulator	1	accessory
group_3894	hypothetical protein	1	accessory
group_3895	hypothetical protein	1	accessory
group_3896	hypothetical protein	1	accessory
group_3897	ADP-ribose-binding domain-containing protein	1	accessory
group_3898	phosphohydrolase	1	accessory
group_3899	protein kinase	1	accessory
group_3900	hypothetical protein	1	accessory
group_3901	hypothetical protein	1	accessory

group_3902	hypothetical protein	1	accessory
group_3903	hypothetical protein	1	accessory
group_3904	hypothetical protein	1	accessory
group_3905	hypothetical protein	1	accessory
group_3906	hypothetical protein	1	accessory
group_3907	resolvase	1	accessory
group_3908	pyrroline-5-carboxylate reductase	1	accessory
group_3909	tRNA-Asn	1	accessory
group_3910	MSHA pilin protein MshA	1	accessory
group_3911	hypothetical protein	1	accessory
group_3912	phage integrase	1	accessory
group_3913	hypothetical protein	1	accessory
group_3914	hypothetical protein	1	accessory
group_3915	hypothetical protein	1	accessory
group_3916	hypothetical protein	1	accessory
group_3917	hypothetical protein	1	accessory
group_3918	hypothetical protein	1	accessory
group_3919	hypothetical protein	1	accessory
group_3920	hypothetical protein	1	accessory
group_3921	hypothetical protein	1	accessory
group_3922	hypothetical protein	1	accessory
group_3923	conjugative relaxase	1	accessory
group_3924	hypothetical protein	1	accessory
group_3925	hypothetical protein	1	accessory
group_3926	hypothetical protein	1	accessory
group_3927	hypothetical protein	1	accessory
group_3928	hypothetical protein	1	accessory
group_3929	hypothetical protein	1	accessory
group_3930	hypothetical protein	1	accessory
group_3931	hypothetical protein	1	accessory
group_3932	hypothetical protein	1	accessory
group_3933	hypothetical protein	1	accessory
group_3934	hypothetical protein	1	accessory
group_3935	hypothetical protein	1	accessory
group_3936	hypothetical protein	1	accessory
group_3937	hypothetical protein	1	accessory
group_3938	hypothetical protein	1	accessory
group_3939	hypothetical protein	1	accessory
group_3940	hypothetical protein	1	accessory

group_3941	hypothetical protein	1	accessory
group_3942	hypothetical protein	1	accessory
group_3943	hypothetical protein	1	accessory
group_3945	hypothetical protein	1	accessory
group_3946	DNA methylase	1	accessory
group_3947	type I restriction endonuclease subunit S	1	accessory
group_3948	hypothetical protein	1	accessory
group_3949	hypothetical protein	1	accessory
group_3950	DEAD/DEAH box helicase	1	accessory
group_3951	hypothetical protein	1	accessory
group_3952	hypothetical protein	1	accessory
group_3953	chemotaxis protein MotB	1	accessory
group_3954	transcriptional regulator	1	accessory
group_3955	hypothetical protein	1	accessory
group_3956	hypothetical protein	1	accessory
group_3957	hypothetical protein	1	accessory
group_3958	hypothetical protein	1	accessory
group_3959	hypothetical protein	1	accessory
group_3960	hypothetical protein	1	accessory
group_3961	phage integrase	1	accessory
group_3962	hypothetical protein	1	accessory
group_3963	deoxyguanosinetriphosphate triphosphohydrolase-like protein	1	accessory
group_3964	hypothetical protein	1	accessory
group_3965	cytosine-specific methyltransferase	1	accessory
group_3966	cytosine-specific methyltransferase	1	accessory
group_3967	hypothetical protein	1	accessory
group_3968	hypothetical protein	1	accessory
group_3969	hypothetical protein	1	accessory
group_3970	colicin immunity protein E2	1	accessory
group_3971	hypothetical protein	1	accessory
group_3972	hypothetical protein	1	accessory
group_3973	hypothetical protein	1	accessory
group_3974	hypothetical protein	1	accessory
group_3975	hypothetical protein	1	accessory
group_3976	hypothetical protein	1	accessory
group_3977	hypothetical protein	1	accessory
group_3978	hypothetical protein	1	accessory
group_3979	hypothetical protein	1	accessory
group_3980	N-acetyltransferase	1	accessory

group_3981	stress protein	1	accessory
group_3982	hypothetical protein	1	accessory
group_3983	hypothetical protein	1	accessory

Table S3. List of CDSs specific for O144 strains

Annotation	Result of NCBI blastp search	Locus tag of V130003
hypothetical protein	hypothetical protein	V130003_01380
hypothetical protein	Qat anti-phage system ATPase QatA	V130003_01410
hypothetical protein	Qat anti-phage system associated protein QatB	V130003_01430
hypothetical protein	Qat anti-phage system QueC-like protein QatC	V130003_01440
TatD family hydrolase	Qat anti-phage system TatD family nuclease QatD	V130003_01450
hypothetical protein	Panacea domain-containing protein	V130003_01460
hypothetical protein	RelA/SpoT domain-containing protein	V130003_01470
ATPase	AAA family ATPase	V130003_01620
hypothetical protein	hypothetical protein	V130003_02470
hypothetical protein	hypothetical protein	V130003_02490
prepilin-type N-terminal cleavage/methylation domain-containing protein	prepilin-type N-terminal cleavage/methylation domain-containing protein	V130003_04820
hypothetical protein	1-deoxy-D-xylulose 5-phosphate reductoisomerase domain protein	V130003_05130
hypothetical protein	DUF262 domain-containing protein	V130003_05170
hypothetical protein	DUF262 domain-containing protein	V130003_05200
6-phosphogluconolactonase	lactonase family protein	V130003_07410
beta-N-acetylhexosaminidase	family 20 glycosylhydrolase	V130003_07420
ketohydroxyglutarate aldolase	bifunctional 4-hydroxy-2-oxoglutarate aldolase/2-dehydro-3-deoxy-phosphogluconate aldolase	V130003_07430
2-dehydro-3-deoxygluconokinase	suger kinase	V130003_07440
sodium:proline symporter	sodium:solute symporter family protein	V130003_07450
reactive intermediate/imine deaminase	Rad family protein	V130003_07460
D-aminoacylase	D-aminoacylase	V130003_07470
aldolase	amino acid deaminase	V130003_07480
transcriptional regulator	MurR/RpiR family transcriptional regulator	V130003_07490
terminase	terminase large subunit	V130003_09400
hypothetical protein	P27 family phage terminase small subunit	V130003_09410
hypothetical protein	HNH endonuclease	V130003_09420
hypothetical protein	phage portal protein	V130003_09430
peptidase	HK97 family phage prohead protease	V130003_09440
hypothetical protein	phage major capsid protein	V130003_09450
hypothetical protein	hypothetical protein	V130003_09460
hypothetical protein	PriCT-2 domain-containing protein	V130003_09470
hypothetical protein	hypothetical protein	V130003_09480
hypothetical protein	helix-turn-helix domain-containing protein	V130003_09490
hypothetical protein	hypothetical protein	V130003_09500
integrase	site-specific integrase	V130003_09510
porin	porin	V130003_10490
UPF0319 protein	DUF2057 family protein	V130003_10500
hypothetical protein	Ig-like domain-containing protein	V130003_10880

hypothetical protein	hypothetical protein	V130003_10930
hypothetical protein	alpha/beta hydrolase fold family protein	V130003_10940
thermostable direct hemolysin 2	thermostable direct hemolysin-family toxin	V130003_10960
hypothetical protein	hypothetical protein	V130003_10990
hypothetical protein	hypothetical protein	V130003_11020
hypothetical protein	VopF	V130003_11040
hypothetical protein	hypothetical protein	V130003_11050
hypothetical protein	EscI/YscI/HrpB family type III secretion system inner rod protein	V130003_11060
type III secretion protein	type III secretion protein	V130003_11070
hypothetical protein	hypothetical protein	V130003_11080
hypothetical protein	regulator	V130003_11090
hypothetical protein	hypothetical protein	V130003_11110
hypothetical protein	type III secretion system translocator protein VopB2	V130003_11120
hypothetical protein	type III secretion system protein	V130003_11130
hypothetical protein	ATP-dependent exonuclease	V130003_11140
hypothetical protein	hypothetical protein	V130003_11150
dimethyladenosine transferase	dimethyladenosine transferase	V130003_11160
hypothetical protein	hypothetical protein	V130003_11170
hypothetical protein	hypothetical protein	V130003_11180
hypothetical protein	VopM	V130003_11190
hypothetical protein	hypothetical protein	V130003_11200
type III secretion protein	FHIPEP family type III secretion protein	V130003_11210
type III secretion protein	EscU/YscU/HrcU family type III secretion system export apparatus switch protein	V130003_11220
outer membrane protein	OmpA family protein	V130003_11230
hypothetical protein	VPA1352 family putative T3SS effector	V130003_11240
hypothetical protein	VPA1351 family putative T3SS effector	V130003_11250
hypothetical protein	VPA1350 family putative T3SS effector	V130003_11260
type III secretion protein	FliM/FliN family flagellar motor switch protein	V130003_11270
hypothetical protein	winged helix-turn-helix domain-containing protein	V130003_11280
hypothetical protein	hypothetical protein	V130003_11290
type III secretion system protein	EscR/YscR/HrcR family type III secretion system export apparatus protein	V130003_11300
hypothetical protein	type III secretion system apparatus protein VscT2	V130003_11310
hypothetical protein	VPA1340 family putative T3SS effector	V130003_11320
secretin	VcsC2	V130003_11330
type III secretion system ATPase	type III secretion system ATPase	V130003_11340
hypothetical protein	VPA1337 family putative T3SS effector	V130003_11350
type III secretion protein	flagellar biosynthetic protein FliQ	V130003_11370
accessory colonization factor AcfA	AcfA family outer membrane beta-barrel protein	V130003_11440
hypothetical protein	IS481 family transposase	V130003_11450
methyl-accepting chemotaxis protein	methyl-accepting chemotaxis protein	V130003_13560

hypothetical protein	hypothetical protein	V130003_13970
hypothetical protein	DUF3466 family protein	V130003_14010
hypothetical protein	bacteriophage f237 ORF9	V130003_14080
hypothetical protein	hypothetical protein	V130003_14100
hypothetical protein	DUF3018 family protein	V130003_14110
toxin	zonular occludens toxin family protein	V130003_14150
hypothetical protein	DUF2523 domain-containing protein	V130003_14160
hypothetical protein	hypothetical protein	V130003_14170
hypothetical protein	hypothetical protein	V130003_14180
hypothetical protein	hypothetical protein	V130003_14190
hypothetical protein	DUF1293 domain-containing protein	V130003_14200
hypothetical protein	replication initiation factor family protein	V130003_14210
hypothetical protein	hypothetical protein	V130003_14220
hypothetical protein	hypothetical protein	V130003_14230
antirepressor	regulator	V130003_14240
biotin synthesis protein BioC	malonyl-ACP O-methyltransferase BioC	V130003_17330
8-amino-7-oxononanoate synthase	8-amino-7-oxononanoate synthase	V130003_17340
integrase	tyrosine-type recombinase/integrase	V130003_19790
hypothetical protein	glycosyltransferase	V130003_19840
hypothetical protein	hypothetical protein	V130003_19850
hypothetical protein	sigma-70 family RNA polymerase sigma factor	V130003_19860
flagellar biosynthetic protein FliP	flagellar type III secretion system pore protein FliP	V130003_19870
hypothetical protein	hypothetical protein	V130003_19880
hypothetical protein	FliM/FliN family flagellar motor C-terminal domain-containing protein	V130003_19890
hypothetical protein	FliM/FliN family flagellar motor switch protein	V130003_19900
flagellar hook protein FlgE	flagellar hook-basal body complex protein	V130003_19910
flagellar hook capping protein	hypothetical protein	V130003_19920
hypothetical protein	hypothetical protein	V130003_19930
hypothetical protein	hypothetical protein	V130003_19940
hypothetical protein	FliI/YscN family ATPase	V130003_19950
hypothetical protein	hypothetical protein	V130003_19960
hypothetical protein	hypothetical protein	V130003_19970
hypothetical protein	flagellar M-ring protein FliF	V130003_19980
hypothetical protein	flagellar hook-basal body complex protein FliE	V130003_19990
hypothetical protein	hypothetical protein	V130003_20000
hypothetical protein	hypothetical protein	V130003_20010
flagellar P-ring protein	flagellar basal body P-ring protein FlgI	V130003_20020
hypothetical protein	flagellar basal body L-ring protein FlgH	V130003_20030
hypothetical protein	flagellar basal body P-ring formation chaperone FlgA	V130003_20040
flagellar basal body rod protein FlgG	flagellar hook-basal body protein	V130003_20050

flagellar basal-body rod protein FlgF	flagellar hook basal-body protein	V130003_20060
flagellar biosynthesis protein FlhA	flagellar biosynthesis protein FlhA	V130003_20070
flagellar biosynthesis protein FlhA	flagellar type III secretion system protein FlhA	V130003_20080
hypothetical protein	EscU/YscU/HrcU family type III secretion system export apparatus switch protein	V130003_20090
hypothetical protein	flagellar biosynthetic protein FliR	V130003_20100
flagellar export apparatus protein FliQ	flagellar biosynthesis protein FliQ	V130003_20110
hypothetical protein	tetratricopeptide repeat protein	V130003_20120
hypothetical protein	tetratricopeptide repeat protein	V130003_20130
hypothetical protein	radical SAM protein	V130003_20140
hypothetical protein	hypothetical protein	V130003_20150
hypothetical protein	SPASM domain-containing protein	V130003_20160
hypothetical protein	histidine kinase domain protein	V130003_20170
hypothetical protein	LysM peptidoglycan-binding domain-containing protein	V130003_20180
hypothetical protein	inovirus Gp2 family protein	V130003_20240
hypothetical protein	aminoglycoside 3'-phosphotransferase	V130003_20700
aminoglycoside O-phosphotransferase APH(6)-Id	streptomycin phosphotransferase B	V130003_20710
hypothetical protein	LysR family transcriptional regulator	V130003_20720
chloramphenicol efflux MFS transporter	chloramphenicol/florfenicol efflux MFS transporter FloR	V130003_20730
hypothetical protein	DUF3363 domain-containing protein	V130003_20740
hypothetical protein	hypothetical protein	V130003_20760
hypothetical protein	hypothetical protein	V130003_20770
hypothetical protein	XRE family transcriptional regulator	V130003_20780
hypothetical protein	hypothetical protein	V130003_20790
hypothetical protein	hypothetical protein	V130003_20800
hypothetical protein	hypothetical protein	V130003_20830
conjugal transfer protein TrbJ	P-type conjugative transfer protein TrbJ	V130003_20840
hypothetical protein	conjugal transfer transcriptional regulator TraJ	V130003_20860
LuxR family transcriptional regulator	transcriptional regulator	V130003_20890
outer membrane protein U	porin	V130003_22210
hypothetical protein	hypothetical protein	V130003_23360
site-specific DNA-methyltransferase	site-specific DNA-methyltransferase	V130003_23390
hypothetical protein	hypothetical protein	V130003_23400
hypothetical protein	hypothetical protein	V130003_23410
hypothetical protein	ABC transporter substrate-binding protein	V130003_23430
hypothetical protein	MSHA biogenesis protein MshQ	V130003_24220
MSHA pilin protein MshA	type II secretion system protein	V130003_24280
putative teichuronic acid biosynthesis glycosyltransferase TuaG	glycosyltransferase family 2 protein	V130003_25640
UDP-glucose 6-dehydrogenase	nucleotide sugar dehydrogenase	V130003_25650
galactosyl transferase	glycosyltransferase family 4 protein	V130003_25850
transport permease protein	ABC transporter permease	V130003_25920

spore coat protein	DegT/DnrJ/EryC1/StrS family aminotransferase	V130003_25930
dTDP-4-dehydrorhamnose 3,5-epimerase	dTDP-4-dehydrorhamnose 3,5-epimerase	V130003_25940
phage-related integrase	site-specific integrase	V130003_26290
hypothetical protein	hypothetical protein	V130003_26300
hypothetical protein	hypothetical protein	V130003_26310
hypothetical protein	hypothetical protein	V130003_26320
hypothetical protein	hypothetical protein	V130003_26330
hypothetical protein	hypothetical protein	V130003_26340
hypothetical protein	hypothetical protein	V130003_26350
hypothetical protein	DUF2787 domain-containing protein	V130003_26380
hypothetical protein	hypothetical protein	V130003_26400
twin-arginine translocation pathway signal	NAD(P)/FAD-dependent oxidoreductase	V130003_26410
sodium-independent anion transporter	sulfate permease	V130003_26420
TIGR01244 family protein	TIGR01244 family phosphatase	V130003_26430
hypothetical protein	MBL fold metallo-hydrolase	V130003_26440
peroxiredoxin	peroxiredoxin	V130003_26450
ribonuclease H	ribonuclease HI	V130003_26460
transcriptional regulator	AlpA family transcriptional regulator	V130003_26470
hypothetical protein	inovirus Gp2 family protein	V130003_26480
hypothetical protein	inovirus Gp2 family protein	V130003_26490
hypothetical protein	hypothetical protein	V130003_26510
hypothetical protein	hypothetical protein	V130003_27130
hypothetical protein	hypothetical protein	V130003_27140
hypothetical protein	hypothetical protein	V130003_27150
hypothetical protein	restriction endonuclease subunit S	V130003_27200
hypothetical protein	DUF4123 domain-containing protein	V130003_27530
hypothetical protein	LysM peptidoglycan-binding domain-containing protein	V130003_27540
hypothetical protein	sel1 repeat family protein	V130003_27550
fucose 4-O-acetylase	acyltransferase	V130003_27580
hypothetical protein	hypothetical protein	V130003_28500
hypothetical protein	DUF1911 domain-containing protein	V130003_28510
hypothetical protein	nicotinamide riboside transporter PnuC	V130003_28590
hypothetical protein	hypothetical protein	V130003_29950
hypothetical protein	hypothetical protein	V130003_29980
hypothetical protein	hypothetical protein	V130003_30150
oxidoreductase	SDR family oxidoreductase	V130003_30380
hypothetical protein	hypothetical protein	V130003_30400
hypothetical protein	hypothetical protein	V130003_30490
glyoxalase	VOC family protein	V130003_30530
hypothetical protein	DUF6232 family protein	V130003_30650

protein phosphatase	dual specificity protein phosphatase family protein	V130003_30760
hypothetical protein	hypothetical protein	V130003_30790
hypothetical protein	helix-turn-helix domain-containing protein	V130003_31420
hypothetical protein	single-stranded DNA-binding protein	V130003_31440
hypothetical protein	helix-turn-helix transcriptional regulator	V130003_31470
hypothetical protein	hypothetical protein	V130003_31490
hypothetical protein	hypothetical protein	V130003_32020
hypothetical protein	hypothetical protein	V130003_32180
hypothetical protein	AAA family ATPase	V130003_32190
hypothetical protein	SIR2 family protein	V130003_33100
hypothetical protein	DUF2235 domain-containing protein	V130003_34410
hypothetical protein	hypothetical protein	V130003_34420
hypothetical protein	hypothetical protein	V130003_34430
hypothetical protein	hypothetical protein	V130003_34630
hypothetical protein	hypothetical protein	V130003_34660
protein-tyrosine-phosphatase	low molecular weight phosphatase family protein	V130003_34700
arsenical-resistance protein	ACR3 family arsenite efflux transporter	V130003_34710
hypothetical protein	metalloregulator ArsR/SmtB family transcription factor	V130003_34720
hypothetical protein	peptidase S24	V130003_34730
hypothetical protein	DUF1611 domain-containing protein	V130003_34740
cold shock protein CspV	cold-shock protein	V130003_34770
transcriptional regulator	helix-turn-helix transcriptional regulator	V130003_34840
peptidase S24	helix-turn-helix domain-containing protein	V130003_34850
hypothetical protein	helix-turn-helix domain-containing protein	V130003_34870
cytosine-specific methyltransferase	DNA cytosine methyltransferase	V130003_34890
cytosine-specific methyltransferase	DNA cytosine methyltransferase	V130003_34900
hypothetical protein	AAA family ATPase	V130003_34910
hypothetical protein	DUF2357 domain-containing protein	V130003_34920
sensor domain-containing diguanylate cyclase	diguanylate cyclase domain protein	V130003_36300
hypothetical protein	tetratricopeptide repeat protein	V130003_36980

Table S4. SNVs detected among O144 strains sequenced in this study

Position on V130003, chromosome 1	V130003	V130004	V130007	V130014	V130021	V130027	V130030	V130055	V130059	V130064	Locus tag (product)	Amino acid substitution
335683	T	C	C	C	C	C	C	C	C	C	V130003_03010 (transcriptional regulator Crp)	Thr145Ala
644631	A	A	A	A	A	A	A	G	A	A	V130003_05780 (C4-dicarboxylate ABC transporter)	Tyr308Cys
1750640	G	G	G	G	G	G	G	G	G	A	V130003_15680 (glucans biosynthesis protein G)	Synonymous mutation
2460305	C	C	C	C	C	C	T	C	C	C	V130003_22110 (translation initiation factor IF-2)	Ala567Thr
2598615	A	G	A	A	A	A	A	A	A	A	non-coding region	-
